# Synthesis and Properties of Bis-Porphyrin Molecular Tweezers: Effects of Spacer Flexibility on Binding and Supramolecular Chirogenesis

**DOI:** 10.3390/molecules21010016

**Published:** 2015-12-23

**Authors:** Magnus Blom, Sara Norrehed, Claes-Henrik Andersson, Hao Huang, Mark E. Light, Jonas Bergquist, Helena Grennberg, Adolf Gogoll

**Affiliations:** 1 Department of Chemistry-BMC, Uppsala University, Uppsala S-75123, Sweden; magnus.blom@kemi.uu.se (M.B.); sara.norrehed@raa.se (S.N.); claes.henrik.andersson@gmail.com (C.-H.A.); hao.huang@angstrom.uu.se (H.H.); jonas.bergquist@kemi.uu.se (J.B.); helena.grennberg@kemi.uu.se (H.G.); 2 Department of Chemistry, University of Southampton, Highfield, Southampton SO17 1BJ, UK; light@soton.ac.uk

**Keywords:** bisporphyrin tweezers, metalloporphyrins, porphyrinoids, host-guest chemistry, supramolecular chemistry, chirogenesis, chirality transfer, exciton coupled circular dichroism, conformational analysis

## Abstract

Ditopic binding of various dinitrogen compounds to three bisporphyrin molecular tweezers with spacers of varying conformational rigidity, incorporating the planar enediyne (**1**), the helical stiff stilbene (**2**), or the semi-rigid glycoluril motif fused to the porphyrins (**3**), are compared. Binding constants K_a_ = 10^4^–10^6^ M^−1^ reveal subtle differences between these tweezers, that are discussed in terms of porphyrin dislocation modes. Exciton coupled circular dichroism (ECCD) of complexes with chiral dinitrogen guests provides experimental evidence for the conformational properties of the tweezers. The results are further supported and rationalized by conformational analysis.

## 1. Introduction

**Figure 1:**
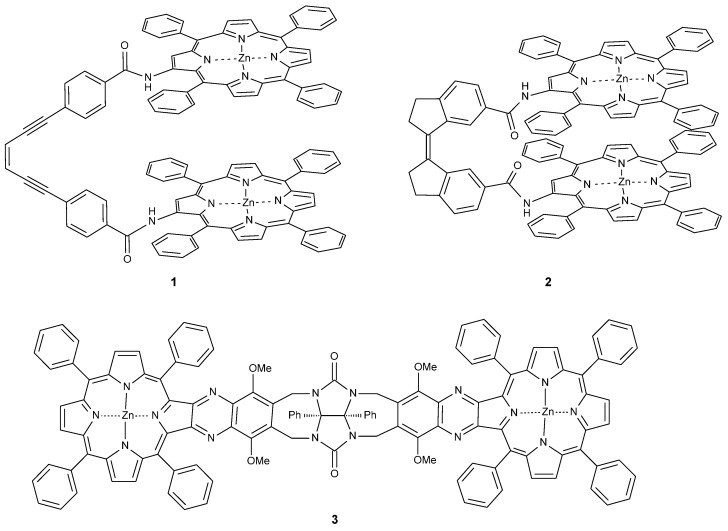
Bisporphyrin molecular tweezers with enediyne (**1**) and stiff stilbene (**2**) spacers, and the previously reported semi-rigid (**3**) with glycoluril spacer.

Bisporphyrin molecular clips and tweezers are well studied systems for ditopic host–guest interactions [1,2]. In the majority of these compounds, two porphyrin chromophores are attached by a single bond to a usually conformationally flexible spacer. They have been used extensively to determine the absolute configuration of guests with a single stereogenic center, or to distinguish enantiomers [3,4,5,6,7,8,9,10,11,12,13,14,15]. We recently have shown that bisporphyrin tweezers also can be utilized for determination of the relative stereochemistry in molecules with several stereocenters, employing a semi-rigid bisporphyrin tweezer **3** incorporating a glycoluril spacer [16,17]. For further investigations, we required alternative tweezers with altered conformational flexibility, a key factor for guest affinity. Also, substitutes for **3** requiring a less demanding synthetic protocol are desirable. Therefore, we decided to replace the glycoluril spacer with enediyne and stiff stilbene spacers, respectively (Figure 1). These tweezers are expected to have restricted conformational flexibility, comparable to other tweezers with spacers composed of aromatic rings and ethyne segments [1]. Since several parameters are involved in host–guest binding, accurate predictions of ligand affinity are not always possible. However, bisporphyrins with flexible spacers are capable of strong binding to dinitrogen ligands. In typical examples, small aliphatic diamines were found to bind with K_a_ = 10^3^–10^5^ M^−1^ to a bisporphyrin with diphenylether spacer, with weaker binding for bulkier guests [5]. Much higher binding constants have been reported for the structurally more rigid DABCO (diazabicyclo[2.2.2]octane) with K_a_ = 10^7^–10^9^ M^−1^ [18,19]. The variation of binding constants with spacer length and flexibility has been explained by preorganization effects when binding rigid guests [20].

Bisporphyrin tweezers have been classified as belonging to three distinct categories regarding the conformational properties of their spacers: spacers with high conformational flexibility, spacers with conformational restrictions, and conformationally rigid spacers [1]. Rigid spacers may favor guest binding due to a preorganization effect, but also prevent binding of guests that cannot be accommodated by bitopic binding [21]. Flexible spacers allow more diverse binding via an induced fit [22,23,24]. The three bisporphyrins **1**–**3** discussed here present a more subtle conformational behavior.

**Figure 2:**
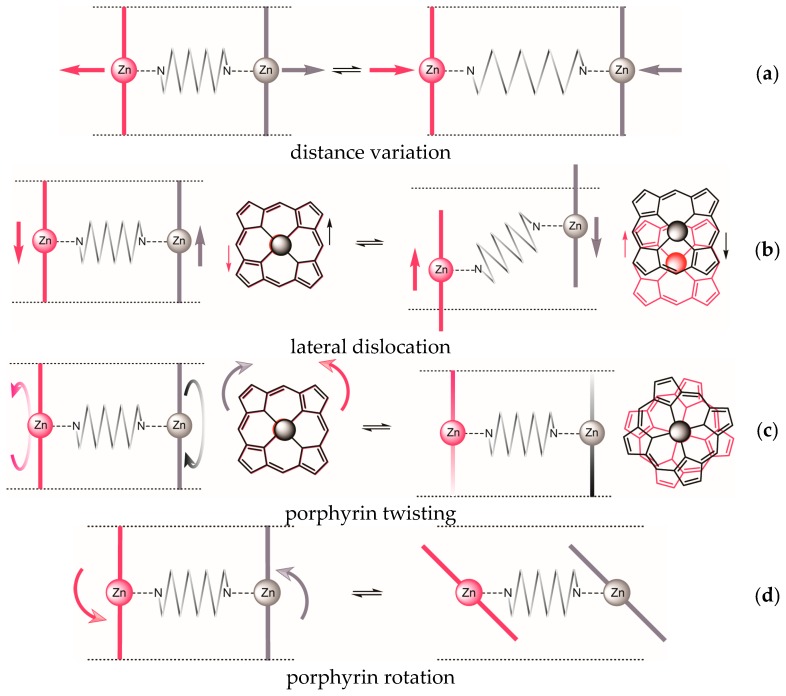
Possible dislocations in bisporphyrin tweezers: (**a**) Variation of interporphyrin distance; (**b**) lateral displacement; (**c**) porphyrin twisting; (**d**) porphyrin rotation.

In principle, four different types of dislocation of the two porphyrin units, some of which might be interdependent, may be distinguished (Figure 2).

For the previously studied glycoluril bisporphyrin tweezer **3**, we have observed conformational flexibility in terms of interporphyrin distance variation (Figure 2a), due to the conformational properties of the glycoluril spacer [16]. This manifested itself experimentally in variation of the hydrodynamic radius of the tweezer upon binding of various guests, as monitored by the diffusion coefficient of the host-guest complex. However, since the porphyrin units are attached to the spacer via two covalent bonds, lateral dislocation (Figure 2b) appears to be unlikely, and porphyrin rotation (Figure 2d) is impossible. There is, however, an option of porphyrin twisting (Figure 2c) via conformational changes of the seven-membered rings in the glycoluril backbone (vide infra). In contrast, both **1** and **2** might allow for lateral dislocation as well as twisting as the result of spacer bond distortion, in combination with porphyrin rotation around single bonds.

It occurred to us that detection of the induced circular dichroism (*i.e.*, exciton coupled circular dichroism, ECCD) [3,4,5,6,7,8,9,10,11,12,13,14,15] that results from binding a chiral diamine guest might provide a simple experimental verification of the distortion modes available to our tweezers (Table 1). Tweezer flexibility and guest geometry determine the sign and amplitude of the detected ECCD, and suitable mnemonics for reliable prediction of the effect are still under development [8,9]. The effect of rigidity variation on CD performance has been addressed for chiral bisporphyrin tweezers without bound guests [25], whereas we here investigate achiral bisporphyrin tweezers. Recently, Rath and co-workers have shown that chirogenesis in a bisporphyrin tweezers requires porphyrin twisting [26].

**Table 1:** Porphyrin dislocation modes (Figure 2) in bisporphyrin tweezers **1**–**3** ^a^.

	Dislocation	1	2	3
a	distance variation	+	(+)	+
b	lateral dislocation	+	inherent	−
c	porphyrin twisting	+	inherent	+
d	porphyrin rotation	+	+	−
helical complex achievable?		+	inherent	−

^a^ Dislocation mode capability is indicated as “+”(possible), “(+)” (requires high energy), “−“ (impossible), “inherent” (present in the lowest energy conformation).

## 2. Results and Discussion

### 2.1. Synthesis

#### 2.1.1. Synthesis of Porphyrin Units and Spacers

**Scheme 1:**
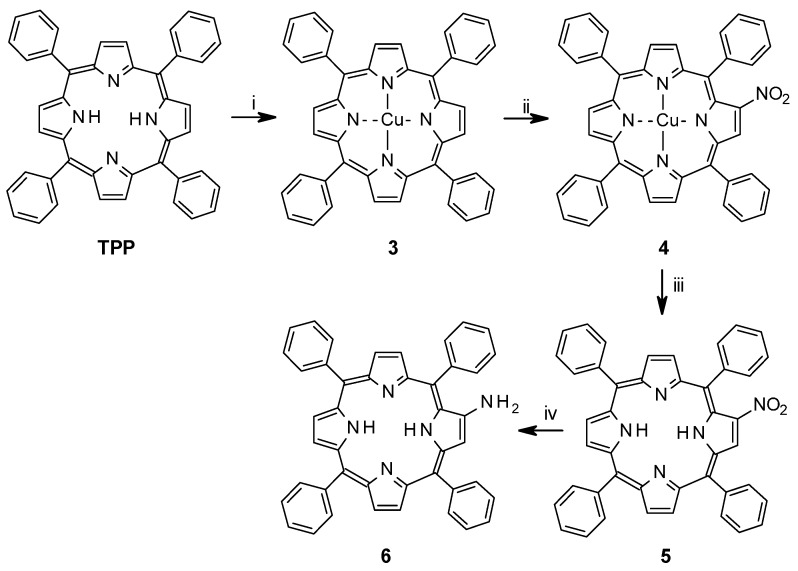
Functionalization of tetraphenyl porphyrin. (i) Cu(OAc)_2_**^.^**H_2_O, CH_2_Cl_2_, MeOH, reflux, 2.5 h; (ii) Cu(NO_3_)_2_**^.^**3H_2_O, acetic acid, acetic anhydride, CHCl_3_, 35 °C, 5 h; (iii) H_2_SO_4_, CH_2_Cl_2_, 20 min r.t.; (iv) SnCl_2_**^.^**2H_2_O, HCl, CHCl_3_ N_2_-atm, dark, r.t., 3–4 days.

The synthesis of bisporphyrin **3** involved six steps from TPP (*meso*-tetraphenylporphyrin), and five steps for the glycoluril spacer, with a total yield of 4% from TPP [16]. For tweezers **1** and **2**, a synthesis scheme was devised involving eight steps (**1**) and six steps (**2**), respectively. Overall yields from TPP were 18% for *Z*-**1** and 19% for *Z*-**2**. Thus, the β-aminoporphyrin (**6**) [27] was prepared starting from the free-base *meso*-tetraphenylpophyrin (TPP) as shown in Scheme 1. Cu(II)TPP (**3**) was obtained in quantitative yield by metallation of TPP via refluxing with a solution of copper(II) acetate in dichloromethane-methanol. Mono-nitration with copper nitrate and acetic anhydride/acetic acid in chloroform [28,29,30] to afford CuTPPNO_2_ (**4**, 80%) was followed by demetallation with sulphuric acid to give TPPNO_2_ (**5**, 95%). Reduction of the nitro group with tin(II)chloride and hydrochloric acid yielded the target compound free-base aminoporphyrin **6**. The initial metallation with copper was performed because β-nitration of a copper metallated porphyrin results in vastly higher yields than nitration of free-base or zinc-metallated porphyrins [31]. Reduction of TPPNO_2_ to TPPNH_2_ with sodium borohydride in the presence of 10% palladium on activated carbon has been suggested as a faster alternative to tin(II)chloride [32], but in our hands it produced a complex mixture of products that was difficult to purify. The aminoporphyrin **6** is highly sensitive towards air and light, and therefore it was prepared immediately before use.

**Scheme 2:**
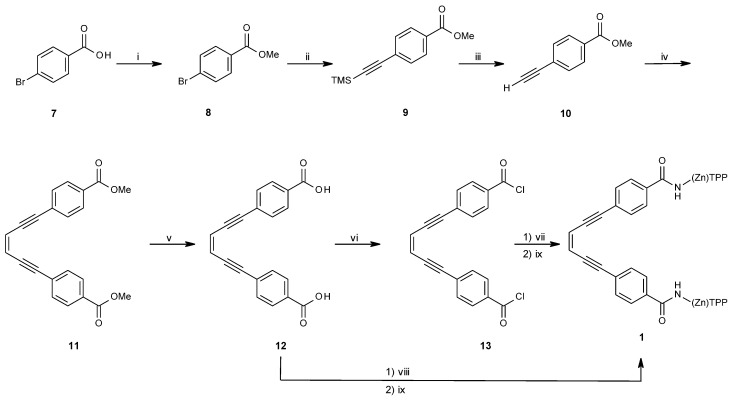
Synthetic route towards enediyne spacer of **1**. (i) Trimethyl orthoacetate, Microwave 110 °C, 1 h; (ii) trimethyl silyl acetylene, Pd(PPh_3_)_2_Cl_2_, CuI, Et_2_NH, DMF, microwave 120 °C, 25 min; (iii) THF, TBAF, −20 °C, 3 h; (iv) 1,2-*Z*-dichloroethylene, Pd(PPh_3_)_2_Cl_2_, CuI, Et_2_NH, toluene, 0 °C, N_2_-atm, 2 days; (v) NaOH, EtOH, reflux, 5 h; (vi) oxalyl chloride, CH_2_Cl_2_/THF, 0 °C, 1 h; (vii) TPPNH_2_, CH_2_Cl_2_, r.t., 12 h; (viii) TPPNH_2_, DCC, CH_2_Cl_2_, r.t., o.n.; (ix) Zn(OAc)_2_**^.^**H_2_O, CH_2_Cl_2_, MeOH, reflux, 30 min.

The synthetic route towards the enediyne spacer of **1** is described in Scheme 2. Microwave assisted esterification of 4-bromobenzoic acid (**7**) afforded 4-bromobenzoic acid methylester (**8**) in 72% yield. A microwave assisted Sonogashira coupling of **8** with trimethylsilyl acetylene yielded the 4-trimethyl silyl protected ethynylbenzoic acid methylester (**9**) almost quantitatively (98%) [33]. Deprotection of the TMS group was carried out with tetrabutylammonium fluoride, which after purification gave 4-ethynylbenzoic acid methylester in 90% yield (**10**) [34]. Methyl-4-[(*Z*)-6-(4-methoxycarbonylphenyl)hex-3-en-1,5-diynyl]benzoate (**11**) was obtained in 70% yield after a second Sonogashira coupling with *Z*-1,2-dichloroethylene [35]. Hydrolysis to dicarboxylic acid **12** (90% yield) was followed by quantitative conversion to its acid chloride **13** by oxalyl chloride, giving the enediyne spacer to be coupled to TPP-NH_2_ **6**.

**Scheme 3:**
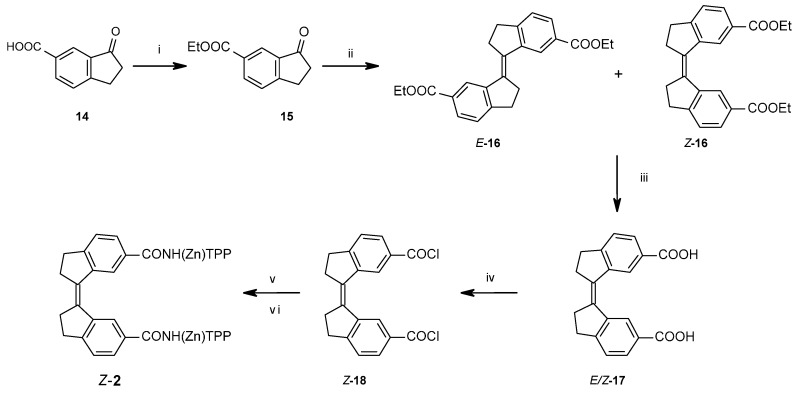
Synthetic route towards stiff stilbene spacer of **2**. (i) EtOH, HCl, reflux, o.n.; (ii) TiCl_4_, THF, Zn(s), reflux, 2 h, add **15**, reflux 12 h; (iii) NaOH, EtOH, reflux, 6 h; (iv) oxalyl chloride, CH_2_Cl_2_, r.t., 2 h; (*v*) TPPNH_2_, CH_2_Cl_2_, r.t., o.n.; (vi) Zn(OAc)_2_**^.^**H_2_O, CH_2_Cl_2_, MeOH, reflux, 30 min.

To obtain the stiff stilbene spacer of **2** (Scheme 3), 3-oxoindane-5-carboxylic acid (**14**) was converted to ethyl 3-oxoindane-5-carboxylate (**15**) via reflux in ethanol in the presence of hydrochloric acid (yield 95%), followed by a reductive McMurry coupling [36] to afford the usual mixture of the *E* and *Z* isomers of ethyl-3-(6-ethoxycarbonylindan-1-ylidene)indane-5-carboxylate **16** (*E*:*Z* = 3:1). Separation of *E*-**16** and enrichment of *Z*-**16** by recrystallization from ethanol, followed by chromatographic purification of *Z*-**16,** afforded pure isomers. Photoisomerization of *E*-**16** was used to produce more of *Z*-**16** [37,38]. Hydrolysis with sodium hydroxide in ethanol afforded dicarboxylic acid *Z*-**17** (94% yield). *Z*-**17** was quantitatively converted to the acid chloride (*Z*-**18**) with oxalyl chloride in dichloromethane. *E*- and *Z*-isomers can be distinguished via the ^3^*J*_HH_ coupling between the olefinic protons, measured on their ^13^C satellites (*E*-isomer: 11 Hz, *Z*-isomer: 5 Hz).

Attempts to use 3-oxoindane-5-carboxylic acid **14** directly as substrate in the reductive coupling were unsuccessful. McMurry has previously discussed functional group compatibility in reductive carbonyl couplings using low-valent titanium reagents in a review [36]. Carboxylic acids were classified as “semi-compatible” for reductive coupling due to their propensity to slowly react with low valent titanium reagents (*i.e.*, TiCl_3_/LiAlH_4_). Consequently, their compatibility is largely limited to conditions where shorter reaction times are employed. Therefore, for the sterically crowded alkene **16**, where longer reaction time is required, a carboxylic acid substrate is less well suited.

#### 2.1.2. Coupling of Spacers to β-Monoaminotetraphenylporphyrin

Coupling of the β-monoaminoporphyrin (**6**) to the enediyne spacer was attempted via the dicarboxylic acid (**12**) using DCC coupling reagent. Although successful, low yields were obtained (up to 14%). Other approaches with DCC/HOBT- and HATU-mediated coupling did not produce any isolable product. An alternative route via the acid chloride (**13**) afforded bisporphyrin tweezer **1** in 25% isolated yield after purification. This route was then also used for the coupling of **6** to acid chloride **18**, producing bisporphyrin tweezer **2** in 24% yield. Low yields in amide formation from aminoporphyrins have been reported previously [39] and are most likely due to the reduced nucleophilicity of the amino group, probably in combination with sterical effects. As metallation of the porphyrin moiety provides stabilization and simplified purification over silica, the crude coupling products were metallated prior to column chromatography. Still, cumbersome purification and solubility problems add to the low yields of these coupling reactions.

### 2.2. Conformational Analysis of Spacer and Tweezer Geometry

**Figure 3:**
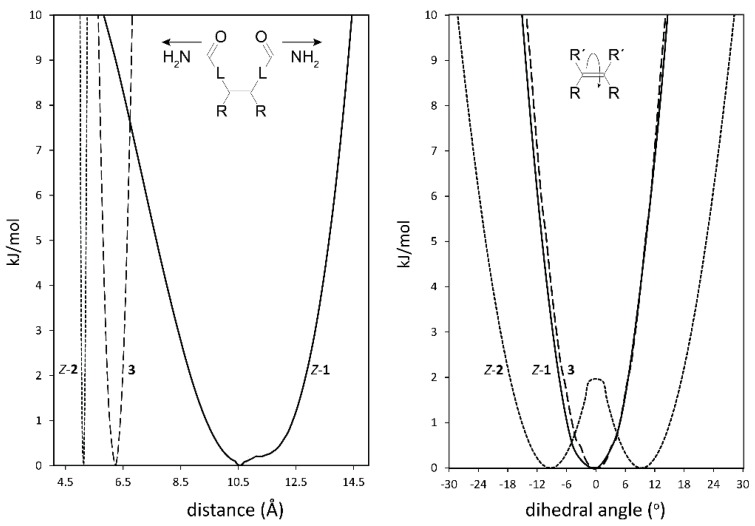
Energy profiles for spacer dihedral angle variation (**right**) and for spacer C=O–C=O distance variation (**left**) in bisporphyrin tweezers *Z*-**1** (**-**), *Z*-**2** (^…^) and **3** (---). L represents the spacer between the double bond and the amide group, e.g., −Ph−C≡C- in **1**. The two minima for tweezer **2** are at ±9°. For **3**, the Zn−Zn distance (**left**), and the dihedral angle between the two phenyl rings attached to the glycoluril unit (**right**, *cf.* Figure 4) in the complete tweezer are shown.

**Figure 4:**
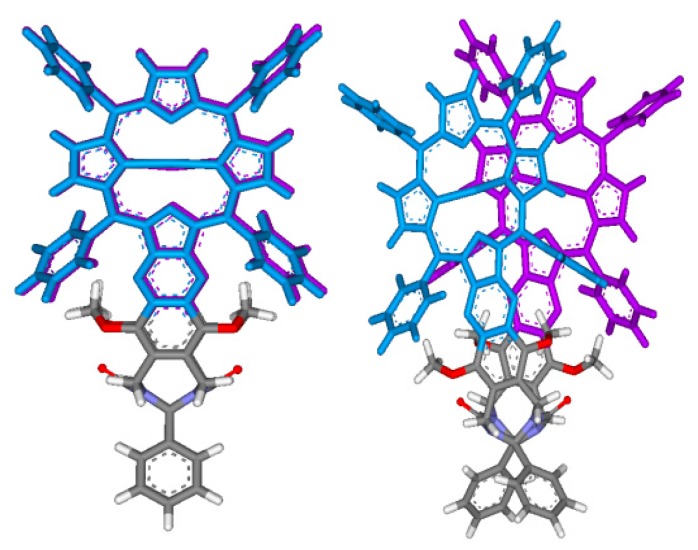
Effect of glycoluril spacer twisting in tweezer **3**, monitored via the Ph-C-C-Ph dihedral angle (viewed along the Ph-C-C-Ph bond, *cf.* Figure 3). **Left**: dihedral angle = 0°, **right**: dihedral angle = 30°.

Indications for the envisioned different steric properties of the spacers in tweezers **1**–**3** were obtained from conformational analysis. Here, we focus on the spacer distortions required to generate the dislocation modes indicated in Figure 2. As expected, there are considerable differences between the three spacers. Regarding the lateral dislocation, *i.e.*, twisting of the two spacers attached to the double bond via hindered double bond rotation (Figure 3, right), tweezer **1** has a narrower profile than tweezer **2**, the latter also having a built-in twisting (*i.e.*, energy minima for a twisted alignment of the two fused ring units attached to the double bond with a 9° dihedral angle), separated by a small local energy maximum at 0° of 1.7 kJ/mol. For tweezer **3**, the energy profile for twisting was monitored by changing the dihedral angle between the two phenyl rings attached to the glycoluril unit (Figure 4). The resulting energy profile resembled closely that of *Z*-**1**.

Regarding the distance variation, relative conformational energies were calculated depending on the distance between the C=O carbons (Figure 3, left). Tweezer **1** has a very broad profile (*ca.* 10.2 Å wide, between 7.7 Å and 13.7 Å within a 5 kJ/mol span) and, thus, should be able to accommodate a variety of guests. In contrast, tweezer **2** shows a much narrower profile (*ca.* 0.2 Å wide, between 5.0 Å and 5.2 Å within a 5 kJ/mol span.

Attachment of the porphyrin units to the spacers results in a less clear cut picture, since interactions between the two porphyrins modulate the energy profiles. However, for tweezer **1** we again obtain a dihedral angle of 0° for the lowest energy conformation, with a Zn–Zn distance of 4.9 Å. For tweezer **2**, the lowest energy conformation has a dihedral angle of 6.5°, with a Zn–Zn distance of 6.2 Å. Tweezer **3** has previously been shown to have a comparatively shallow energy profile upon Zn–Zn distance variation, covering at least ≈10 Å, with a minimum for a Zn–Zn distance of 6.25 Å [16].

Regarding these energy profiles, it should be kept in mind that, according to Berova and co-workers [11], only host–guest conformers within an energy span of 10 kJ/mol above the minimum were considered as relevant contributors to the CD spectrum (*vide infra*).

We can summarize the tweezer conformational properties as follows. Tweezer **1** is capable of all four distortions indicated in Figure 2. This should enable binding of a large variety of diamine guests with similar binding constants. Porphyrin rotation is not necessary in order to accommodate sterically demanding guests. In tweezer **2**, distance variation is limited, and therefore larger guests can be accommodated only by increased spacer twisting and/or porphyrin rotation. Both of these introduce helicity. Tweezer **3** can apparently vary the interporphyrin distance and achieve porphyrin twisting via glycoluril conformational changes, which also might induce helicity.

### 2.3. Conformational Analysis of Host-Guest Complex Geometry

**Scheme 4:**
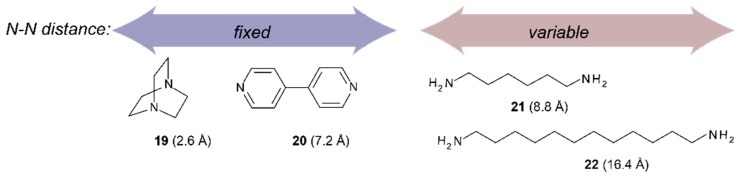
Maximal distance between nitrogen atoms for the guest molecules used in this study.

**Scheme 5:**
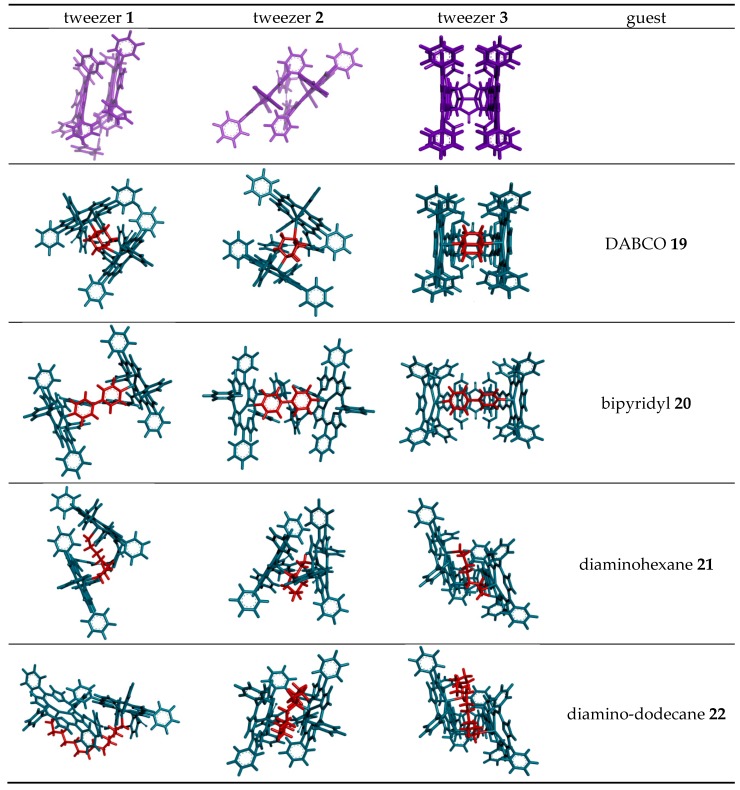
Porphyrin dislocations in complexes of tweezers **1**–**3** (green color) with various dinitrogen guests (red color). Shown are the structures corresponding to the global minimum obtained in conformational search with the OPLS 2005 force field.

Conformational analysis results of tweezer complexes with dinitrogen guests (Scheme 4) are summarized in Scheme 5 and Table 2. They show a considerable variation of Zn–Zn distances in complexes involving tweezers **1** and **3**, and less in those with tweezer **3**. The latter accommodates larger but flexible guests by twisting (complexes with **21** and **22**), whereas tweezers **1** and **2** both show porphyrin rotation, or lateral dislocation (only **1**). Higher energy conformers did not exhibit any substantial deviations from these geometries. These structures should be interpreted with some caution, though. For example, the complex of 1,12-diaminododecane **22** with tweezer **1** gives the lowest energy for an in-out binding mode (Scheme 5), which is not in agreement with experimental evidence, such as ^1^H-NMR data (*vide infra*).

**Table 2:** Dihedral angles, spacer C=O–C=O distances, and Zn**–**Zn distances (Å) from conformational analysis of tweezers **1**–**3** with bound guests.

Guest	Host						
	Z-1			Z-2			3
	Dihedral Angle ^a^	CO–CO Distance	Zn–Zn Distance	Dihedral Angle ^a^	CO–CO Distance	Zn–Zn Distance	Zn–Zn Distance
(free tweezer)	0°	8.5 Å	4.9 Å	6.5°	4.9 Å	6.1 Å	6.3 Å ^b^
DABCO **19**	0.2°	7.9 Å	7.0 Å	11.2°	5.4 Å	7.3 Å	7.3 Å
4,4′-bipyridyl **20**	0.1°	10.6 Å	11.3 Å	11.5°	5.9 Å	11.6 Å	11.5 Å
1,6-diaminohexane **21**	0.5°	7.1 Å	8.4 Å	10.4°	5.7 Å	5.8 Å	8.5 Å
1,12-diaminododecane **22**	^c^	^c^	^c^	10.5°	4.8 Å	6.1 Å	8.2 Å
Lys methylester **23**	0.3°	6.9 Å	9.9 Å	8.5°	5.3 Å	8.5 Å	11.3 Å

^a^ Dihedral angle over spacer double bond; ^b^ Ref. [16]; ^c^ Minimizes to in-out complex (*cf.* Scheme 5).

### 2.4. X-ray Crystallography of the Stiff Stilbene Spacer

**Figure 5:**
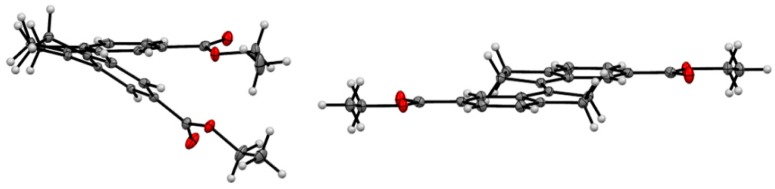
ORTEP view of *Z*-**16** (**left**) and *E*-**16** (**right**). Thermal ellipsoids are drawn at the 35% probability level.

X-ray crystallographic analysis of diester **16**, a congener of the spacer of tweezer **2**, supports the results from conformational analysis, namely the effect of sterical interaction between the double bond substituents in the *Z*-isomer. Whereas the *E-*isomer is completely planar (dihedral angle between the aromatic carbons attached to the double bond = 180.0(1)°), the corresponding angle in the *Z*-isomer is 9.1(2)°. Transannular interaction between the aromatic rings results in an even larger angle when measured on the two carbonyl carbons, at 27.30(8)° (Figure 5).

This is in keeping with other 1,1′-biindanylidene derivatives. The geometry of the stiff stilbene unit appears to be predominantly dependent on steric interactions. All structures reported in the CCDC database are symmetrical with respect to the central double bond. In the *E*-isomers, the torsional angle between the two indane subunits (measured as the torsional angle involving the double bond and the two ortho positions of the fused phenyl rings) varies between 180.00(4)° for unsubstituted indanyl units [40] and 138.6(2)° for 2,2,2′,2′-tetramethyl-indanyl rings [41]. Also, ortho substituents on the phenyl rings result in deviations from the 180° angle, such as 154.39(7)° in (*E*)-7,7′-dimethyl-1,1′-biindanylidene [41]. For *Z*-isomers, compounds devoid of indane substituents with steric interactions show torsional angles in the range of approximately 24.90(9)° for (*Z*)-(1,1′)biindanylidene [42] and 18.7(1)° for (*Z*)-6,6′-dimethyl-1,1′-biindanylidene [43]. Substituents on the cyclopentyl ring, in particular dimethyl substitution such as in *Z*-2,2,2′,2′-tetramethyl-1,1′-biindanylidene (41.2(1)°) [43] or on the phenyl ring, such as ortho-methyl substituents in (*Z*)-4,4′,7,7′-tetra-methyl-1,1′-biindanylidene (41.4(1)°) [44], result in the largest deviations from this value. The diester **16** shows an undistorted geometry, with a torsional angle for *E*-**16** at 180.00(7)°, and for *Z*-**16** at 24.1(1)°, which is close to the values measured for unsubstituted stiff stilbenes (*i.e.*, approximately 20°) [37].

### 2.5. Binding of Dinitrogen Guests

#### 2.5.1. UV-Vis Spectroscopy

**Figure 6:**
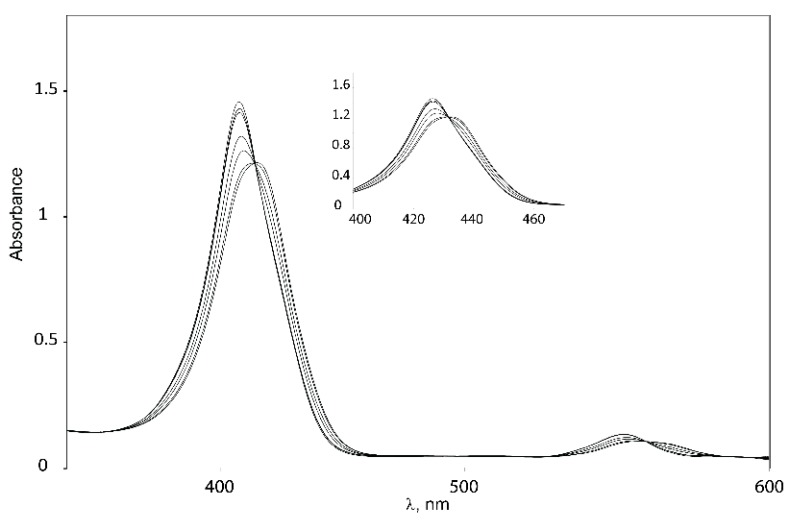
Isosbestic points for binding of 1,6-diaminohexane **21** to Z-**1**.

**Figure 7:**
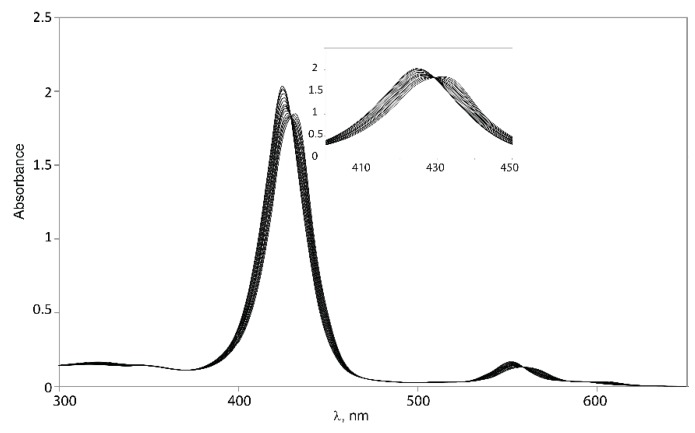
Isosbestic points for binding of 1,6-diaminohexane **21** to Z-**2**.

Binding studies with tweezers **1**–**3** and a series of dinitrogen guests (Scheme 4), with both variable and fixed N−N distances, were performed to probe the impact of conformational flexibility on binding affinity. If we compare their maximal N−N distances with those obtained from the calculated Zn−Zn distances of their complexes with tweezer **3**, subtraction of twice the assumed Zn−N bond length (2.2 Å) [26] gives the N−N distance of bound guest, which is 2.62 Å (**19**), 6.76 Å (**20**), 7.84 Å (**21**) and 8.12 Å (**22**), respectively, indicating coiling of the flexible guests **21** and, in particular, **22**. As shown by pronounced isosbestic points (Figure 6 and Figure 7), accompanied by red shifts of the Soret- and Q-bands, formation of a single, well defined complex is indicated (Table 3) [10,45,46] which, according to NMR data (*vide infra*), is a 1:1 complex (see Supplementary Materials).

The consistent increase of the red shifts observed with larger N−N distances of the guests indicates the formation of 1:1 host–guest complexes [47]. Red shifts due to amine binding may be counteracted by the blue shift resulting from close proximity of two porphyrin rings [8,48]. Thus, the two guests with the shortest N−N distance (DABCO **19** and 4,4′-bipyridyl **20**) produce the smallest red shifts for their complexes. For **20**, the smaller red shift is also caused by the different electron density at its nitrogens, as indicated by the smaller red shift for the complex with Zn-TPP.

Complexes with glycoluril tweezer **3** show the smallest red shifts, which would indicate a closer proximity between the two porphyrin units than for the complexes with **1** and **2**. Another factor that could account for the smaller red shifts exhibited in the complexes with tweezer **3** is the degree of porphyrin rotation. Since tweezer **3** has no possibility of porphyrin rotation, the porphyrin rings should preserve their coplanarity with respect to the glycoluril backbone, and thus the blue shifts caused by π–π interactions between the porphyrin moieties should be larger than for **1** and **2** at the same Zn−Zn distances.

Binding constants of the investigated host–guest complexes (Table 4) show the expected effect of cooperativity when compared to the reference Zn-TPP, with K_a_ being up to three orders of magnitude larger for the tweezers. Amongst these, the largest binding constants are observed for **1**, followed by **3**, and with **2** showing the weakest binding. We rationalize this as follows. Tweezer **2** has the least flexible backbone, and therefore can accommodate guests only at the expense of distortion, primarily backbone twisting. The backbone of tweezer **1** is more flexible, and the cost of distortion upon guest accommodation is therefore low, resulting in larger binding constants. For the previously reported tweezer **3**, backbone distortion via conformational variation of the glycoluril rings also requires relatively low energies, giving high binding constants. Here, the possible distortions appear to be variation of interporphyrin distance and porphyrin twisting. Both **1** and **2** have an additional distortion available, *viz.* porphyrin rotation, which is not available to tweezer **3**. It should be emphasized that the larger flexibility of **1**, as compared to both **2** and **3**, which might result in a lesser degree of preorganization, is not manifested in the binding constants. Instead, it appears that tweezer **1** allows for an induced fit of guest molecules.

**Table 3:** UV-vis red shift of Soret bands upon binding (Δλ = λ_complex_ − λ_tweezer_) for molecular tweezers *Z*-**1**, *Z*-**2** and **3** (CH_2_Cl_2_ solution, r.t.).

Guest	(Δλ = λ_complex_ – λ_tweezer_) Host			
	Z-1	Z-2	3	Zn-TPP
DABCO **19** ^a^	5	5	4 ^b^	10
4,4′-bipyridyl **20**	7	7	5	8
1,6-diaminohexane **21**	9	9	10 ^b^	10
1,12-diaminododecane **22**	10	10	10	10

^a^ 1,4-Diazabicyclo[2.2.2]octane; ^b^ CHCl_3_ solution.

**Table 4:** Binding constants (K_a_, M^−1^) (CH_2_Cl_2_ solution, r.t.) for molecular tweezers *Z*-**1**, *Z*-**2** and **3** with selected dinitrogen compounds, determined by UV-vis titration.

Guest	Host K_a_/M^−1^			
	Z-1	Z-2	3	Zn-TPP ^a^
DABCO **19** ^b^	2 × 10^4^	4 × 10^5^	3 × 10^6 c^	8 × 10^3^
4,4′-bipyridyl **20**	4 × 10^6^	2 × 10^5^	2 × 10^6^	-
1,6-diaminohexane **21**	6 × 10^6^	5 × 10^5^	2 × 10^6 c^	5 × 10^4^
1,12-diaminododecane **22**	8 × 10^5^	2 × 10^5^	2 × 10^5^	-

^a^ Zn-meso-Tetraphenylporphyrin; ^b^ 1,4-Diazabicyclo[2.2.2]octane; ^c^ CHCl_3_ solution.

It is interesting to note that both **1** and **3** show a 10 times lower binding constant for 1,12-diaminododecane **22** than for diaminohexane **21**, whereas **2** shows more similar binding constants for both guests. Obviously, the large guest **22** does not fit well into the binding cavity of any tweezer, despite coiling, and tweezer **2**, in particular, cannot vary its interporphyrin distance to improve binding, as is the case for **1** and **3**. For the smallest guest, DABCO **19**, tweezer **1** has the lowest binding constant, which is probably due to the difference between interporphyrin distance in the free tweezer and in the complex, resulting in only a small preorganization effect. In tweezer **1**, the Zn−Zn distance is 4.9 Å, as compared to 7.0 Å in the complex (Table 2). For tweezer **2**, the Zn−Zn distance increases only slightly from 6.2 Å (tweezer) to 7.3 Å (complex). However, these numbers have to be interpreted with some caution. Guest binding requires distortion of tweezer geometry (the source of the ECCD observed in CD spectroscopy, *vide infra*). As illustrated in Scheme 5 for binding of tweezers **1** and **2** to DABCO (**19**), for a rigid guest that itself cannot contribute to complex stability by conformational distortion and that has been used to test binding modes for bisporphyrins [49,50], geometries of free and complexed tweezers differ substantially.

#### 2.5.2. NMR Spectroscopy

**Figure 8:**
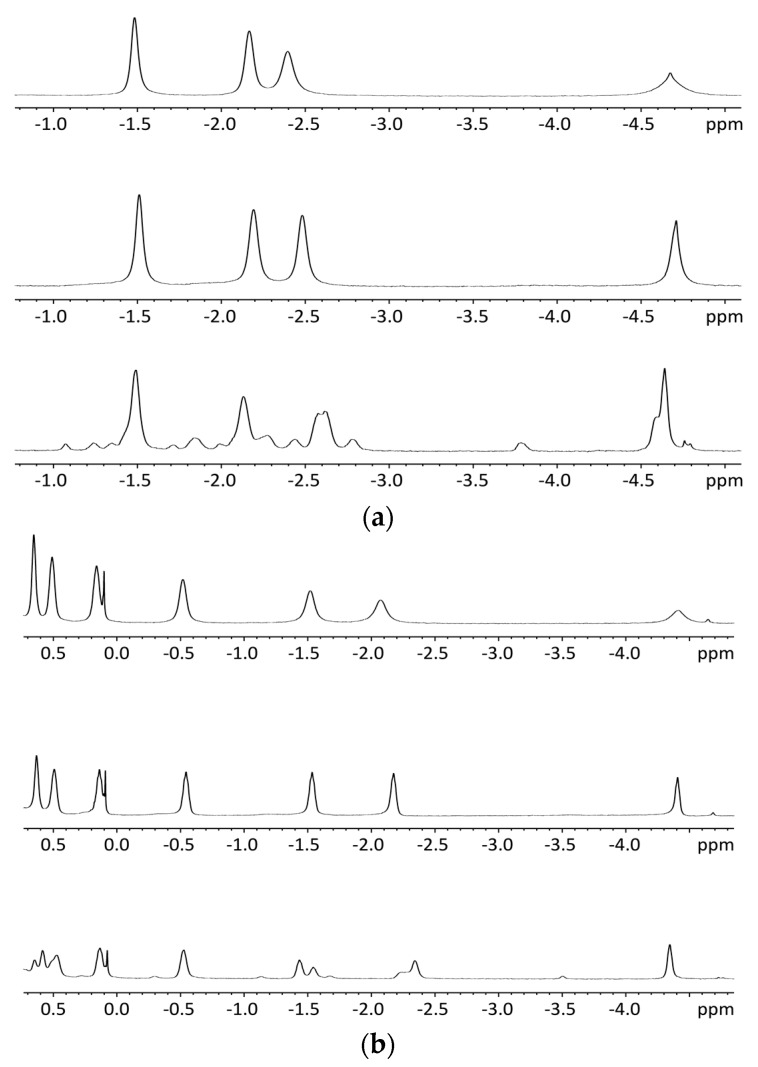
Variable temperature ^1^H-NMR spectra (500 MHz), (**a**): 1:1 complex of enediyne tweezer *Z*-**1** and 1,6-diaminohexane **21** in CDCl_3_. Top: 25 °C Middle: 0 °C Bottom: −55 °C; (**b**): 1:1 complex of enediyne tweezer *Z*-**1** and 1,12-diaminododecane **22** in CDCl_3_. Top: 25 °C, Middle: 0 °C, Bottom: −55 °C.

**Figure 9:**
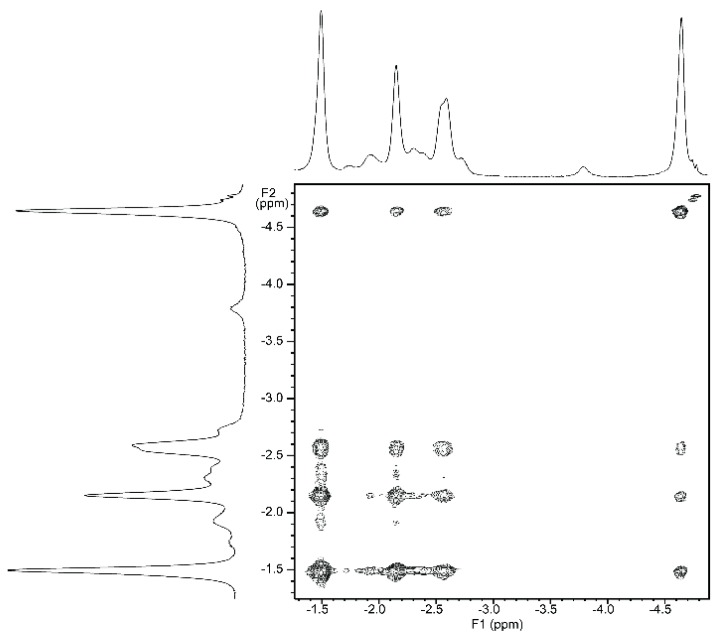
Expansion from TOCSY spectrum of a ≈1:1 complex of enediyne tweezer Z-**1** and 1,6-diaminohexane **21** (500 MHz, CDCl_3_. solution, −40 °C).

**Figure 10:**
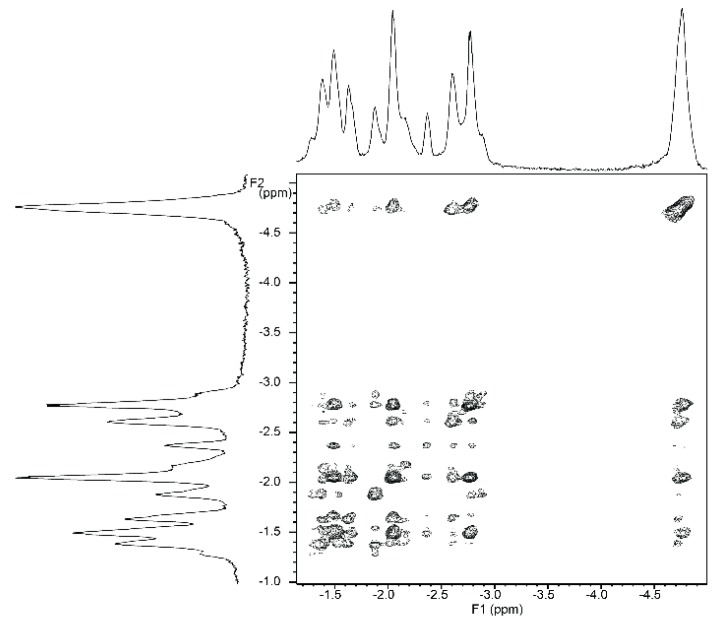
Expansion from TOCSY spectrum of a ≈1:1 complex of tweezer Z-**2** and 1,6-diaminohexane **21** (500 MHz, CDCl_3_. solution, −40 °C).

NMR titrations of tweezer **1** with 1,ω-diamino-n-alkanes were also consistent with ditopic binding, showing (n + 2)/2 signals at low chemical shifts (Figure 8). For 1:1 complexes, low temperature ^1^H-NMR spectra showed an increase in signal number. While the broadening at 25 °C indicates an onset of dynamics, possibly guest dissociation, the observation of an increased signal number at lower temperatures is likely due to the presence of several conformers of the host–guest complex that are no longer exchanging. At reduced temperature, most CH_2_ signals show a slightly decreased chemical shift (Figure 8), and TOCSY spectra show the presence of several isolated spin systems, each with the signal number expected for one ditopically bound diamine guest molecule (Figure 9 and Figure 10).

A possible explanation for the larger number of guest signals for 1,6-diaminohexane **21** in the complex with stiff stilbene tweezer *Z*-**2** as compared to that with the enediyne tweezer *Z*-**1** might be the reduced inter-porphyrin distance flexibility imposed by the stiff stilbene spacer. In order for guest **21** to fit into the cavity, the tweezer sidewalls of Z-**2** have to twist and rotate in relation to each other, resulting in two non-equivalent porphyrin faces in contact with the guest. This type of signal splitting has been observed previously [49].

#### 2.5.3. Circular Dichroism

**Figure 11:**
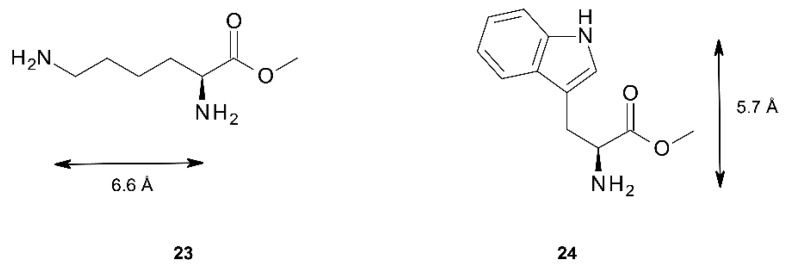
Chiral diamines **23** and **24** used in CD studies. The maximal N–N distances are indicated.

Further information on the binding properties of the three tweezers should be accessible by binding studies involving chiral diamino compounds, *i.e.*, by utilizing the chirogenesis that is the foundation of exciton coupled circular dichroism (ECCD). Parameters involved in this phenomenon have been extensively studied, and models to rationalize the effect have been proposed [3,4,5,6,7,8,9,10,11,12,13,14,15]. We found it tempting to test this effect by exposing the sterically flexible lysine methylester **23**, and the more rigid tryptophan methylester **24** (Figure 11) to tweezers **1**–**3**. Both esters have previously been shown to be capable of chirogenesis with bisporphyrin hosts [9,13,51]. While **23** constitutes a diamino ligand, **24** binds monotopically, but may give a strong CD response with suitable bisporphyrin tweezers.

**Figure 12:**
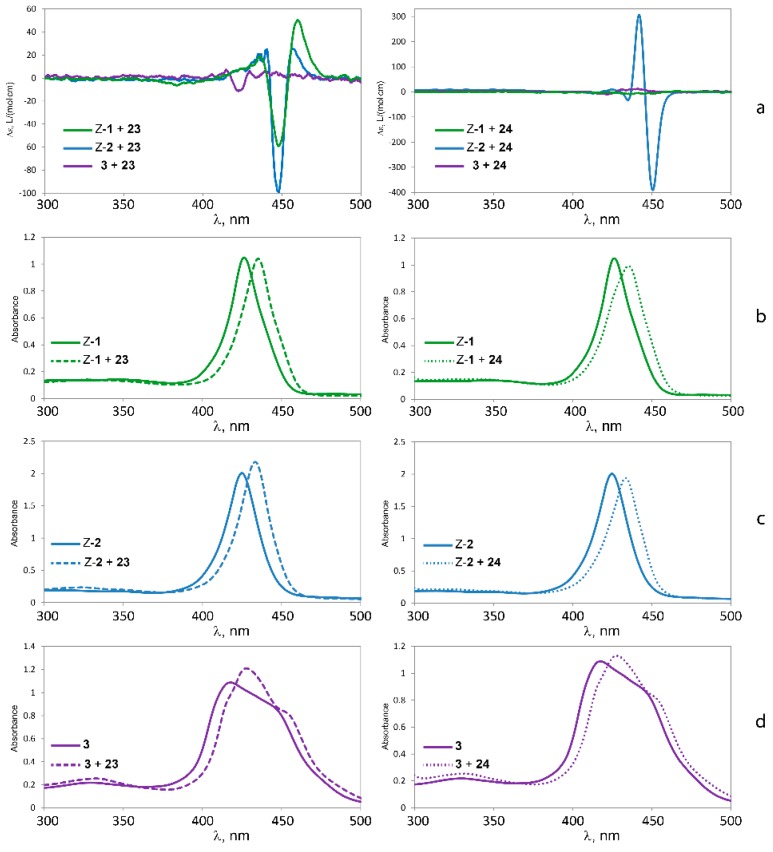
CD spectra (**a**) and UV-Vis spectra of l-lysine methylester **23** (**left**) and l-tryptophan methylester **24** (**right**) bound to tweezers **1** (**b**); **2** (**c**) and **3** (**d**).

As illustrated in Figure 12, there is indeed a relationship between the rigidity of the bisporphyrin tweezer and the observed CD signal. Tweezer **3** shows no chirality transfer from both **23** and **24**. In contrast, strong CD-signals for both **23** and **24** when binding to *Z*-**2** demonstrate a high degree of induced conformational helicity (Table 5). Tweezer *Z***-1** on the other hand produces a slightly weaker ECCD than *Z***-2** for the complex with the flexible chiral lysine methylester **23**, but none at all for tryptophan methylester **24**, despite succesful binding. The reason for this might be the already twisted spacer in *Z*-**2**, which favors unidirectional porphyrin twisting in the presence of a chiral guest, which is to some extent reminescent of bisporphyrins with Tröger’s base as chiral spacer, which also have produced very strong CD signals [25]. The enediyne spacer, on the other hand, shows no twisting in the absence of a guest, and might bind a chiral guest without preference for porphyrin twisting in a particular direction, which would result in cancellation of the CD signal. Such a situation has been described by Rath and co-workers, based on X-ray crystallography [26]. However, there are also examples where tryptophan methylester **24** bound to bisporphyrin tweezers fails to give a CD signal at all [9]. Monotopic binding of **24** via its α-amino group is a likely reason for absence of chirality transfer to *Z*-**1**, which leaves the second (non-binding) porphyrin ring free to assume random orientations. This is supported by the absence of complexation between indole and Zn-TPP (see Supplementary Materials). Furthermore, similar observations for **24** bound to bisporphyrin tweezers with flexible pentanediol as opposed to tweezers with rigid melamine spacer have been reported [9].

**Table 5:** CD signal amplitudes (L × mol^−1^ × cm^−1^) for the complexes of tweezers Z-**1** and Z-**2** with chiral guests **23** and **24** at 20-fold excess. Measurements were performed at 25 °C for CH_2_Cl_2_ solutions at tweezer concentration of 33 µM.

Guest	Host					
	*Z*-1		*Z*-2		3	
	λ/Δε	A_CD_	λ/Δε	A_CD_	λ/Δε	A_CD_
**23**	460 nm (+50)	+109	457 nm (+26)	+125	-	
	448 nm (−59)		448 nm (−99)			
**24**	-		451 nm (−391)	−697	-	
			442 nm (+306)			

**Figure 13:**
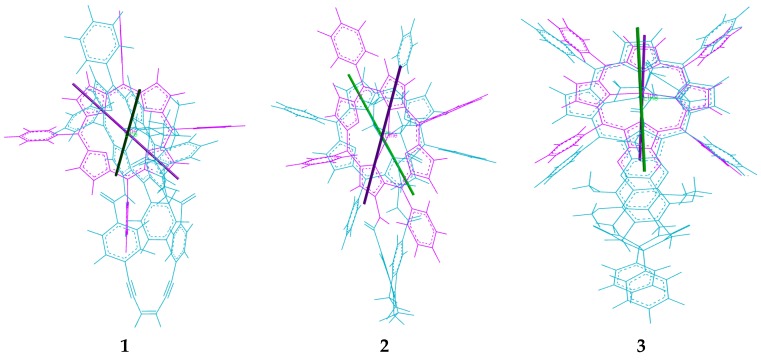
Alignment of porphyrin axes in the complexes with chiral guest lysine methylester **23**. Line positions are indicated for **23** with tweezers **1**, **2** and **3**, respectively.

The absence of chirality transfer from both **23** and **24** to tweezer **3** is surprising, since twisting of the porphyrin units is possible (*cf.* Figure 4). However, if we inspect the calculated structures of the complexes between the three tweezers and these guests, there is a striking difference: If we compare the angle between lines bisecting the porphyrin units from the amide-attached pyrrol ring to the one on the opposite side (seen along the connecting line between their centers), this angle is close to 0° for the complexes of tweezer **3**. For complexes with tweezer **1**, the angle is 60°–70°, for those with tweezer **2** it is 35°–45° (Figure 13). This is reminiscent of rationalization of the ECCD produced by bisporphyrins by the effective transition moment in porphyrin derivatives [52].

Thus, for the purpose of chiral transfer, it seems to be beneficial with a tweezer exhibiting a more restricted inter-poprhyrin distance since this forces the porphyrins to translocate and rotate in order to accommodate the guest. It is interesting to note that tryptophan methylester with tweezer **2** produces a strong CD signal in dichloromethane solution, while previously typical bisporphyrins with flexible spacer used for this purpose have been reported to generate no CD signals in this solvent [9], requiring the use of less polar solvents such as methyl cyclohexane. Furthermore, tweezer **2** shows a CD signal already at low guest concentrations (≥1:1 host:guest ratio), while typical systems require an appreciable excess of ligand (≥6:1).

## 3. Experimental Section

### 3.1. General

Commercially available compounds were used without purification. *meso*-Tetraphenylporphyrin was purchased from Porphyrin Systems GbR, Germany. Microwave heating was carried out in a Biotage Initiator microwave instrument using 10–20 mL Biotech microwave vials, applying microwave irradiation at 2.4 GHz, with a power setting up to 400 W, and an average pressure of 3–4 bar. Analytical TLC was performed using Merck precoated silica gel 60 F_254_ plates and for column chromatography Matrex silica gel (60 Å, 35–70 μm) was used. Melting points were determined using a Stuart Scientific melting point apparatus SMP10 and are uncorrected. Molecular structures for tweezers **1**–**3**, as well as their spacers, were calculated in MacroModel 9.9 with the OPLS-2005 force field [53] and a dielectric constant of 9.1. The coordinate scan option was used to obtain energy profiles for dihedral angle scan about the double bond of the alkene unit, or for alteration of distances between the two halves of the tweezer or spacer (Figure 3). Conformational analysis was performed with the lowest energy conformation from these scans as the starting structure. For bisporphyrins and host–guest complexes, Zn–N distances were initially constrained to 2.0 Å, resulting in final Zn–N distances of 2.1–2.3 Å [9,11]. In complexes of **24**, the ligand was constrained to ditopical binding (involving both nitrogens) to keep it within the bisporphyrin cleft.

^1^H- and ^13^C-NMR spectra were recorded on Varian Mercury Plus (^1^H at 300.03 MHz, ^13^C at 75.45 MHz), Varian Unity (^1^H at 399.98 MHz, ^13^C at 100.58 MHz), or Varian Unity Inova (^1^H at 499.94 MHz, ^13^C at 125.7 MHz) spectrometers at 25 °C unless noted otherwise. Chemical shifts are reported referenced to tetramethylsilane via the residual solvent signal (CDCl_3_, ^1^H at 7.26 and ^13^C at 77 ppm; DMSO-*d*_6_, ^1^H at 2.50 and ^13^C at 39.5 ppm). Signal assignments were derived from COSY [54,55], P.E.COSY [56], gHSQC [57], gHMBC [58], gNOESY [59], ROESY [60], and TOCSY [61] spectra.

Mass spectra were recorded on a GCQ/Polaris MS spectrometer using direct inlet interface (EI-MS), on an Advion Expression-L CMS with APCI interface, or on an Ultraflex II MALDI TOF/TOF (Bruker, Rheinstetten, Germany) mass spectrometer equipped with a gridless delayed extraction ion source, a 337-nm nitrogen laser, and a gridless ion reflector, using an α-cyano-4-hydroxycinnamic acid matrix (MALDI-MS). HR-MS were acquired using a Thermo Scientific LTQ Orbitrap Velos apparatus in direct nanospray infusion mode.

Circular dichroism spectra were recorded for solutions in CH_2_Cl_2_ on a JASCO J-810 spectropolarimeter from 300–500 nm using a 0.1 cm path length quartz cell. CD-spectra were measured in millidegrees and normalized into Δε_max_ (L·mol^−1^)/λ (nm) units. For comparison with literature data, molar ellipticities θ were converted to A_CD_ values with A_CD_ = θ/32.982.

UV-Vis spectra were recorded on a Varian Cary 3 Bio spectrophotometer using 5 mm or 10 mm quartz cuvettes. K_a_ for diamines were determined by UV-Vis titration utilizing the iterative fitting program (in Matlab R2012b) recently published by P. Thordarsson [62]. A 1:1 complexation model and global fitting to several datasets was applied. K_a_ is calculated by (Equation (1)). The standard error (SEy) is estimated by (Equation (2)). For NMR titrations, aliquots of freshly prepared guest solutions (CDCl_3_, AlOx-filtered) were added to a solution of tweezer **1** or **2** in an NMR tube.

(1) $$\left[HG\right]=\frac{1}{2}\left\{\left({\left[G\right]}_{0}+\;{\left[H\right]}_{0}+\;\frac{1}{K_{a}}\right)-\sqrt{{\left({\left[G\right]}_{0}+\;{\left[H\right]}_{0}+\;\frac{1}{K_{a}}\right)}^{2}-4{\left[H\right]}_{0}{\left[G\right]}_{0}}\right\}$$

(2) $$SE_{y\;}=\;\sqrt{\frac{\sum^{\text{​}}{\left(ydata-ycalc\right)}^{2}}{N-k}}$$

CCDC 1437031 and 1437032 contain the supplementary crystallographic data for this paper. These data can be obtained free of charge via http://www.ccdc.cam.ac.uk/conts/retrieving.html (or from the CCDC, 12 Union Road, Cambridge CB2 1EZ, UK; Fax: +44 1223 336033; E-Mail: deposit@ccdc.cam.ac.uk).

Synthesis of compounds **3**, **4**, **5**, **6**, **8**, **9**, **10**, **1**5, **23** and **24**: See Supplementary Materials.

### 3.2. Syntheses

*Methyl 4-[(Z)-6-(4-methoxycarbonylphenyl)hex-3-en-1,5-diynyl]benzoate* (*Z*-**11**). This protocol is a modification of one previously reported for *Z*-enediyne oligomers [35]. Pd(PPh_3_)_2_Cl_2_ (51 mg, 0.064 mmol), *n*-butylamine (450 mg) and dry benzene (6 mL) were added to a 25 mL round bottomed flask. The stirred mixture was cooled to 0 °C on an ice bath, before addition of methyl 4-(ethynyl)benzoate **10** (320 mg, 2.0 mmol) and (*Z*)-1,2-dichloroethylene (96 mg, 1.0 mmol). Subsequently, copper(I)iodide (34 mg, 0.18 mmol) was added and the mixture was allowed to stir at 0 °C for two hours and then at room temperature for two days. The dark reaction mixture was washed with 1 M HCl, (sat.) NaHCO_3_ solution and brine before separating the phases. The aqueous phase was then extracted three times with diethyl ether, and the combined organic phases were dried over Na_2_SO_4_ before removal of solvent by reduced pressure. The resulting black solid was purified by column chromatography on silica using pentane as eluent giving the product *Z*-**11** as dark solid (240 mg, yield 70%). R_f_ = 0.42 (*n*-pentane/EtOAc = 5:1). ^1^H-NMR (400 MHz, CDCl_3_) δ = 8.02 (AA′BB′, 4H), 7.56 (AA′BB′, 4H), 6.16 (s, 2H, CH=CH), 3.93 (s, 6H, OCH_3_). ^13^C-NMR (100.6 MHz, DMSO-*d*_6_) δ = 165.5, 131.7, 129.7, 129.6, 126.6, 120.7, 96.7, 90.1, 52.4. MS (EI): *m/z* = 344 ([M + H]^+^), 313 ([M–CH_3_O]^+^). UV-vis: (CH_2_Cl_2_) λ_max_: 345, 280 nm.

*Methyl 4-[(E)-6-(4-methoxycarbonylphenyl)hex-3-en-1,5-diynyl]benzoate* (*E*-**11**). ^1^H-NMR (500 MHz, CDCl_3_) δ = 8.01 (AA′BB′, 4H, Ar-H), 7.52 (AA′BB′, 4H, Ar-H), 6.33 (s, 2H, CH=CH), 3.92 (6H, OCH_3_). ^13^C-NMR (100.6 MHz, CDCl_3_) δ = 166.4, 129.9, 129.6, 129.5, 127.3, 120.1, 94.5, 89.8, 52.3.

*4-[(Z)-6-(4-carboxyphenyl)hex-3-en-1,5-diynyl]benzoic acid* (*Z*-**12**). Dimethylester *Z*-**11** (0.5 g, 1.45 mmol) was dissolved in ethanol (100 mL) and NaOH (0.23 g, 5.8 mmol) was added. The mixture was refluxed for 1.5 h and followed by TLC. Water (150 mL) was added to the mixture, followed by a dropwise addition of HCl (12 M) upon which a fine-particle precipitate formed. The mixture was filtrated several times and the product *Z*-**12** was obtained as beige solid (0.409 g, 1.3 mmol, 90%). ^1^H-NMR (400 MHz, *d*_6_-DMSO) δ = 13.12 (br s, 2H, OH), 7.98 (AA′BB′, 4H, Ar-H) 7.63 (AA′BB′, 4H, Ar-H), 6.45 (s, 2H, CH=CH, ^3^*J*_HH_ = 11 Hz, measured on the ^13^C satellites). ^13^C-NMR (100.6 MHz, DMSO-*d*_6_) δ = 166.6, 131.6, 131.0, 129.7, 126.2, 120.6, 96.8, 89.8. IR (KBr): 3433 cm^−1^ (broad), 1689 cm^−1^. HRMS, calcd. for C_20_H_12_O_4_: *m*/*z* = 317.0808 [M + H]^+^, found 317.0805.

*4-[(Z)-6-(4-chlorocarbonylphenyl)hex-3-en-1,5-diynyl]benzoyl chloride* (*Z*-**13**). Dicarboxylic acid *Z*-**12** (50 mg, 0.16 mmol) was dissolved in THF (10 mL) and 2 drops of DMF was added. The mixture was cooled to 0 °C and oxalyl chloride (0.1 mL, 1.1 mmol) added upon which gas formation was observed. The mixture was allowed to stir for 1 h after which solvents were evaporated to yield an orange solid (*Z*-**13**) that was used without further purification (57 mg, quant.) ^1^H-NMR (400 MHz, CDCl_3_) δ = 8.11 (AA′BB′, 4H), 7.60 (AA′BB′, 4H), 6.22 (s, 2H, CH=CH). MS (EI) *m*/*z*: 352 [M]^+^, 317 [M − Cl]^+^.

*Z-1,6-Bis-[4-(5,10,15,20-tetraphenylporphyrin-2-yl-ato)Zn(II)carbamoylphenyl]-hexa-1,5-diyn-3-ene (Z-***1***)*

#### Alternative Route 1

Dicarboxylic acid *Z*-**12** (25 mg, 0.08 mmol) was dissolved in dry and degassed THF (5 mL). DCC (40 mg, 0.19 mmol) was added and the mixture was allowed to stir for 5 min. The mixture was then added via syringe to a solution of TPPNH_2_ (100 mg, 0.16 mmol) in dry and degassed THF (20 mL) under N_2_-atm, dark, at 0 °C. The mixture was left to warm to r.t. and stir over night. Urea precipitate was filtered off and solvents evaporated to give a dark purple-greenish crude solid The crude solid was dissolved in CH_2_Cl_2_ (50 mL), MeOH (5 mL) and Zn(OAc)_2_·H_2_O (0.5 g) was added. The mixture was refluxed for 20 min and allowed to cool to r.t. and solvents evaporated. The reaction residue was purified by column chromatography (eluent CH_2_Cl_2_) from which the pink fraction showed to contain product. Solvents were evaporated to give a purple solid of crude precursor to *Z*-**1** that was used directly for metallation (19 mg, 14%). MS (MALDI-TOF), calculated for C_108_H_70_N_10_O_2_, [M + H]^+^, *m*/*z* = 1540. found: *m*/*z* = 1540.

#### Alternative Route 2

Dicarboxylic acid chloride *Z-***13** (31 mg, 0.08 mmol) was dissolved in dry and degassed THF (3 mL), cooled to 0 °C and added via syringe to a solution of TPPNH_2_ (141 mg, 0.22 mmol) in dry and degassed THF (20 mL) under N_2_-atm in the dark. The mixture was left to warm to r.t. and stir over night. MeOH (10 mL) and Zn(OAc)_2_·H_2_O (0.7 g) was added and left to stir at 50 °C for 3 h. Solvents were evaporated to give a crude dark purple solid that was purified by column chromatography (eluent CH_2_Cl_2_) to give a purple solid of *Z*-**1** (34 mg, 0.02 mmol, 25%). R_f_ = 0.1 (dichloromethane). ^1^H-NMR (400 MHz, CDCl_3_) δ = 9.76 (s, 2H, H-3′), 9.22 (br s, 2H, NH), 8.94 (d, *J* = 4.7 Hz, 2H, H-7′/8′), 8.91–8.89 (m, 6H, H-7′/8′, H-12′, H-13′), 8.85 (d, *J* = 4.7 Hz, 2H, H-17′), 8.47 (d, *J* = 4.7 Hz, 2H, H-18′), 8.33–8.29 (m, 3H, Ph-20), 8.24–8.15 (m, 12H, Ph), 7.87–7.84 (m, 5H, Ph-20) 7.78–7.68 (m, 20H, Ph), 7.64 (AA′BB′, 4H, H-8), 7.45 (AA′BB′, 4H, H-9), 6.27 (s, 2H, CH=CH). ^13^C-NMR (100.6 MHz, CDCl_3_) δ = 163.7 (CO), 152.0, 150.8, 150.6, 150.1, 150.0, 149.6, 149.1, 142.6, 141.7, 141.4, 140.5, 139.8, 134.2, 134.19, 134.17, 133.7, 132.8, 132.7, 132.3, 132.2, 131.8, 131.0, 129.0, 128.5, 127.6, 127.1, 126.7, 126.6, 126.2, 124.8, 122.0, 121.5, 121.0, 120.6 (C-3′), 120.2 (C-3), 117.2, 97.4 (C-1), 89.9 (C-2). UV-Vis (CH_2_Cl_2_) λ_max_: 427 nm (ϵ = 6.4 × 10^5^ M^−1^·cm^−1^), 552 nm (ϵ = 5.6 × 10^4^ M^−1^·cm^−1^). MS (MALDI-TOF): *m*/*z* = 1667 [M + H]^+^, 1689 [M + Na]^+^. HRMS: *m*/*z* calculated for C_108_H_70_N_10_O_2_, [M + 2H]^2+^: 834.2206, found 834.2032.


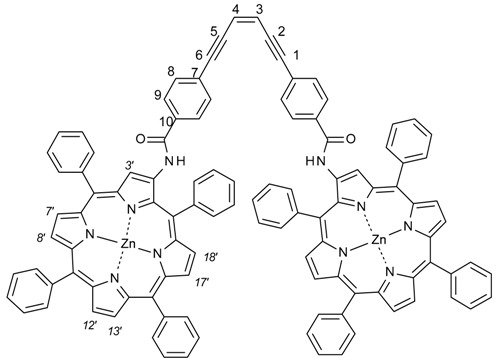


*Ethyl 3-(6-ethoxycarbonylindan-1-ylidene)indane-5-carboxylate* (**16**). To a suspension of TiCl_4_ (1.394 g, 7.35 mmol) in dry THF (30 mL) solvent was slowly added Zn powder (0.96 g, 14.7 mmol) under an Argon atmosphere. The resultant deep green slurry was heated at reflux for 2 h. A THF solution (10 mL) of ethyl-3-oxoindane-5-carboxylate (**15**) (500 mg, 2.45 mmol) was added to the mixture in one portion and refluxed for 12 h. After all starting material was consumed (TLC), the mixture was quenched with saturated aqueous NH_4_Cl and extracted with diethyl ether. The organic phase was filtered through MgSO_4_ and solvents evaporated. The yellow crude product was purified by column chromatography (eluent CH_2_Cl_2_:pentane 2:3) from which *E*-**16** (pale yellow solid) and *Z*-**16** (yellow oil) were isolated (324 mg, 0.86 mmol, 70% (*E*:*Z* = 3:1).

*Ethyl (3E)-3-(6-ethoxycarbonylindan-1-ylidene)indane-5-carboxylate* (*E*-**16**). R_f_ = 0.73 (dichloromethane). ^1^H-NMR (500 MHz, CDCl_3_) δ = 8.26 (2H, d, *J* = 1.5 Hz, H-4), 7.91 (2H, dd, *J =* 1.5, 7.8 Hz, H-6), 7.37 (2H, dm, *J =* 7.8 Hz, H-7), 4.41 (4H, q, *J =* 7.1 Hz, OC*H_2_*), 3.27 (4H, m, CH_2_-2), 3.19 (4H, m, CH_2_-1), 1.42 (6H, t, *J =* 7.1, OCH_2_C*H_3_*). *E*-configuration indicated by NOE between H-4 and H-2′. ^13^C-NMR (100.6 MHz, CDCl_3_) δ = 167.0 (CO), 152.5 (C-7a), 143.2 (C-3a), 135.4 (C-3), 129.0 (C-5), 128.5 (C-6), 125.5 (C-4), 124.8 (C-7), 60.9 (O***C***H_2_), 31.9 (CH_2_-2), 31.2 (CH_2_-1), 14.4 (CH_2_***C***H_3_). IR 1703 [ν(C=O)] cm^−1^. MS *m*/*z* (EI): 376 [M + H]^+^.


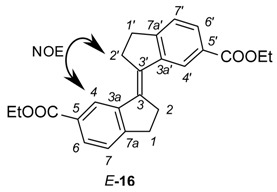


*Ethyl (3Z)-3-(6-ethoxycarbonylindan-1-ylidene)indane-5-carboxylate* (*Z*-**16**). R_f_ = 0.43 (dichloromethane). ^1^H-NMR (500 MHz, CDCl_3_) δ = 8.76 (2H, d, *J =* 1.6 Hz, H-4), 7.89 (2H, dd, *J =* 1.6, 7.9 Hz, H-6), 7.35 (2H, dm, *J =* 7.9, H-7), 4.32 (4H, q, *J =* 7.1 Hz, OC*H_2_*), 3.04 (4H, m, CH_2_-1), 2.85 (4H, m, CH_2_-2), 1.31 (6H, t, *J =* 7.1 Hz, OCH_2_C*H_3_*). ^13^C-NMR (100.6 MHz, CDCl_3_) δ = 166.6 (CO), 153.3 (C-7a), 140.4 (C-3a), 135.0 (C-3), 128.9 (C-6), 128.3 (C-5), 125.0 (C-7), 124.2 (C-4), 60.7 (CO***C***H_2_), 34.7 (CH_2_-2), 30.7 (CH_2_-1), 14.2 (CH_2_***C***H_3_); MS *m/z* (EI) 376 [M + H]^+^.


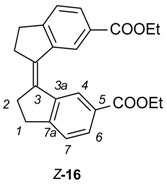


Preparative isomerizations of *E*-**16** followed by chromatographic purification were used to get access to larger quantities of *Z*-**16**.

#### Photoisomerizations

Photoisomerizations were conducted for CDCl_3_ solutions at 25 °C using an Oriel 1000 W Xe ARC light source equipped with a band pass filter 20BPF10-340 (for *Z*-**1** and *Z*-**11**) or 10BPF10-300 (for *Z*-**2** and *Z*-**16**) (Newport). Solutions were degassed by argon bubbling for 15 min prior to irradiation. As reaction vessels, 5 mm NMR-tubes, Type 5Hp, 178 mm were used. The course of the irradiation was followed by NMR-spectroscopy (Varian Unity Inova 500 MHz NMR spectrometer, ^1^H at 499.9 MHz).

*General procedure for hydrolysis of Z/E stiff stilbene diethyl ester 16 to 3-(6-carboxy-indan-1-ylidene)indane-5-carboxylic acid* (**17**). Z- or E- stiff stilbene diethylester **16** (120 mg, 0.32 mmol), NaOH (150 mg, 3.75 mmol) and ethanol (20 mL) was added to a round-bottomed flask and the mixture was refluxed at 80 °C for 6 h. The solvent was removed in vacuo and the residue was dissolved in H_2_O. The aqueous solution was acidified by addition of HCl (6 M) and the formed a yellow precipitate was collected by filtration (using a grade 3 filter paper). The solid product (**17**) was washed with H_2_O and dried overnight.

*Z*-Dicarboxylic acid *Z*-**17** (96 mg, 94%). ^1^H-NMR (500 MHz, dmso-*d*_6_, 25 °C) δ = 8.54 (2H, d, *J* = 1.5 Hz, H-4), 7.79 (2H, dd, *J* = 1.5, 7.8 Hz, H-6), 7.46 (2H, dm, *J* = 7.8 Hz), 3.02 (4H, m), 2.83 (4H, m). ^13^C-NMR (100.6 MHz, dmso-*d*_6_) δ = 167.6, 153.7, 140.33, 135.3, 129.2, 129.1, 125.9, 123.8, 34.7, 30.6.

*E*-Dicarboxylic acid *E*-**17** (83 mg, 82%). ^1^H-NMR (500 MHz, dmso-d_6_, 25 °C) δ = 8.17 (2H, d, *J* = 1.5 Hz, H-4), 7.84 (2H, dd, *J* = 1.5, 7.8 Hz, H-6), 7.48 (2H, d, *J* = 7.8 Hz, H-7), 3.18 (8H, m, CH_2_CH_2_). ^13^C-NMR (75.5 MHz, dmso-*d*_6_, 25 °C) δ = 167.9, 152.7, 143.1, 135.5, 129.7, 129.0, 125.6, 125.5, 31.8, 31.0. HRMS, calc. for C_20_H_16_O_4_: *m*/*z* = 321.1121 [M + H]^+^, found 321.1118.

*(3Z)-3-(6-Chlorocarbonylindan-1-ylidene)indane-5-carbonyl chloride* (**18**). Dicarboxylic acid *Z*-**17** (55 mg, 0.17 mmol) was added to a dried 25 mL round-bottomed flask together with dry CH_2_Cl_2_ (5 mL), oxalyl chloride (0.9 mL) and DMF (3 drops). The mixture was stirred at RT under nitrogen atmosphere for two hours. Volatile components were removed *in vacuo* and the residue was washed several times with dry Et_2_O in which the product was soluble. The heterogeneous Et_2_O solution was filtrated (using a grade 3 filter paper) to remove by-products and the filtrate was evaporated to leave the acid chloride *Z*-**18** as a pale yellow solid (yield: 45 mg, 74%). ^1^H-NMR (500 MHz, CDCl_3_, 25 °C) δ = 8.80 (2H, d, *J* = 1.8 Hz, H-4), 7.96 (2H, dd, *J* = 1.8, 8.0 Hz, H-6), 7.43 (2H, dm, *J* = 8.0 Hz, H-7), 3.10 (4H, m, CH_2_), 2.90 (4H, m, CH_2_).

*(3Z)-N-(5,10,15,20-tetraphenylporphyrin-2-yl-ato)Zn(II)-3-[6-(5,10,15,20-tetraphenylporphyrin-2-yl-ato)Zn(II)-carbamoyl)indan-1-ylidene]indane-5-carboxamide* (2). TPP-NH_2_ 6 (210 mg, 0.33 mmol) was added to a dried round-bottomed flask and *Z*-stif-stilbene dicarboxylic acid chloride *Z*-18 (42 mg, 0.13 mmol) dissolved in dry CH_2_Cl_2_ (20 mL) was added via syringe. Dry pyridine (0.2 mL) was added and the mixture was allowed to stir for three days under nitrogen atmosphere and with protection from light. The solvent was evaporated and the residue redissolved in CH_2_Cl_2_. The solution was washed with HCl (1 M, 50 mL) and brine (5%, 50 mL). The organic phase was then dried over Na_2_SO_4_ and the solvent evaporated. The crude compound was added to a flask together with Zn(OAc)_2_·2H_2_O (1 g), methanol (20 mL), CH_2_Cl_2_ (20 mL) and the mixture was stirred at 50 °C for one hour. The solvents were removed by evaporation and the crude metallated compound was purified by column chromatography (silica, CH_2_Cl_2_) followed by a subsequent silica column (CH_2_Cl_2_:methanol:acetic acid 96:4:1). The compound was further purified by washing several times with methanol to yield the target compound *Z*-2 as a light/air sensitive purple solid (yield: 53 mg, 24%). R_f_ = 0.13 (dichloromethane). ^1^H-NMR (500 MHz, CDCl_3_, 25 °C) δ = 9.25 (2H, s, H-3′), 8.81 (2H, br s, N-H), 8.78 (2H, d, *J* = 4.5 Hz, β-pyrrole), 8.78 (2H, d, *J* = 4.5 Hz, β-pyrrole), 8.76 (2H, d, *J* = 4.5 Hz, β-pyrrole), 8.72 (2H, d, *J* = 4.6 Hz, β-pyrrole), 8.43 (2H, d, *J* = 4.6 Hz, β-pyrrole), 8.38 (2H, d, *J* = 4.6 Hz, β-pyrrole), 8.11 (2H, m, H-4), 8.11–8.07 (8H, m, Ph), 8.01 (4H, m, H-d), 7.77–7.66 (12H, m, Ph), 7.63 (2H, m, H-f), 7.46 (4H, m, H-e), 7.43 (4H, m, H-a), 7.38 (2H, d, *J* = 7.4 Hz, H-7), 7.09 (2H, m, H-6), 7.08 (2H, m, H-c), 6.90 (4H, m, H-b), 3.22 (4H, m, CH_2_-2), 3.05 (4H, m, CH_2_-1). ^13^C-NMR (125 MHz, CDCl_3_, 25 °C) δ = 164.8, 152.0, 150.7, 150.6, 150.2, 149.9, 149.6, 149.5, 149.0, 142.7, 142.6, 142.1, 141.4, 141.1, 140.0, 139.8, 135.3, 134.4, 134.3, 134.2, 133.8, 133.3, 133.0, 132.0, 131.8, 131.7, 131.5, 131.4, 130.9, 128.8, 128.1, 127.43, 127.40, 127.0, 126.7, 126.6, 126.50, 126.46, 126.1, 124.9, 121.9, 121.6, 121.0, 120.23, 120.19, 117.4, 35.3, 31.0. Z-2 UV-Vis (CH_2_Cl_2_) λ_max_: 425 nm (ϵ = 6.0 × 10^5^ M^−1^·cm^−1^), 552 nm (ϵ = 5.1 × 10^4^ M^−1^·cm^−1^). MS (MALDI-MS, dithranol, positive mode) *m/z*: 1669 [M + H]^+^). HRMS: *m/z* calcd. for C_108_H_70_N_10_O_2_Zn_2_, [M + H]^+^: 1669.4269; found: 1669.4193.


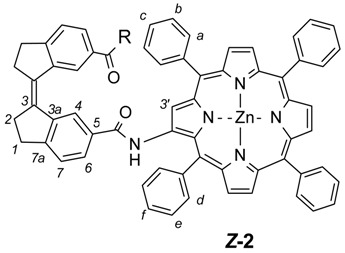


## 4. Conclusions

The Zn-porphyrin tweezers **1**, **2**, and **3**, with differing conformational flexibility, have been investigated. Differences in spacer flexibility affect their possibilities for interporphyrin distance variation, porphyrin rotation, translocation and twisting upon ditopic binding to dinitrogen guest molecules. These differences result in a variation of binding constants, and are further detected via NMR spectra of host–guest complexes. CD spectroscopy offers a further possibility to monitor distortions of the tweezer geometry in the complexes. Flexibility regarding interporphyrin distance variation is the single most important factor that contributes to stronger binding. However, a higher degree of preorganization owing to limited porphyrin rotation as in **3** does not result in higher binding constants when compared to tweezer **1** with the most flexible spacer. Tweezer **2** has the least flexible spacer, hence weaker binding, but due to “built-in” helicity, it shows the highest CD signal amplitudes.

## Supplementary Materials

Supplementary materials can be accessed at: http://www.mdpi.com/1420-3049/21/1/16/s1.
