# Supplementary material for: Synthesis and Properties of Bis-Porphyrin Molecular Tweezers: Effects of Spacer Flexibility on Binding and Supramolecular Chirogenesis
Source: Molecules. 2015 Dec 23;21(1):16. doi: 10.3390/molecules21010016 (PMC6274253; doi:10.3390/molecules21010016)
Supplement: Supplementary file 1 [file molecules-21-00016-s001.pdf]

Supplementary Materials: Synthesis and Properties of Bis-Porphyrin Molecular Tweezers: Effects of Spacer Flexibility on Binding and Supramolecular Chirogenesis

Magnus Blom, Sara Norrehed, Claes-Henrik Andersson, Hao Huang, Mark E. Light, Jonas Bergquist, Helena Grennberg [1] and Adolf Gogoll

1. Syntheses

(5,10,15,20-Tetraphenylporphyrinato)copper(II), Cu(II)TPP (**3**) [1]. *meso*-Tetraphenylporphyrin (TPP) (2 g, 3.25 mmol) was dissolved in CH_2Cl_2 (160 mL) and methanol (50 mL). $Cu(OAc)_2{\cdot}H_2O$ (1.2 g, 5.85 mmol) was added and the mixture was heated to reflux for 2 h until all starting material was consumed (TLC, UV-vis). Solvents were evaporated to give a red-purple residue that was filtered through a short plug of silica. After filtration, the product **3** was obtained as a dark purple sparkling solid (2.2 g, 3.25 mmol, 99%) UV-vis: (CH_2Cl_2) λ_{max}: 415, 539 nm.

(2-Nitro-5,10,15,20-tetraphenylporphyrinato)copper(II), Cu(II)TPPNO₂ (**4**) [2]. Cu(II)TPP (**3**) (0.7 g, 1.0 mmol) was dissolved in chloroform (700 mL) and acetic acid (15 mL) was added. $Cu(NO_3)_2{\cdot}3H_2O$ (0.63 g, 2.6 mmol) was dissolved in acetic anhydride (70 mL) and added to the reaction mixture. The mixture was heated to 35 °C and let to stir for 5 h. The reaction mixture was washed with water (3 × 700 mL), saturated K_2CO_3 solution (2 × 700 mL) and again with water (2 × 700 mL), dried over anhydrous Na_2SO_4 and solvents removed by azeotropic evaporation with methanol. The residue was purified by column chromatography using CH_2Cl_2:pentane 1:1 as eluent resulting in Cu(II)$TPPNO_2$ (**4**) as a dark purple solid (0.65 g, 0.89 mmol, 89%) UV-vis: (CH_2Cl_2) λ_{max}: 415, 544, 589.

2-Nitro-5,10,15,20-tetraphenylporphyrin, $TPPNO_2$ (**5**) [1]. Cu(II)$TPPNO_2$ (**4**) (1 g, 1.4 mmol) was dissolved in dichloromethane (200 mL) and konc. H_2SO_4 (6 mL) was added. The reaction mixture was let to stir for 2 h until all starting material was consumed (TLC) and then poured into an ice-water bath (100 mL). The phases were separated and the water phase extracted with CH_2Cl_2 (3 × 100 mL). The combined organic phases were washed with water (2 × 300 mL), 10% $NaHCO_3$ solution (2 × 300 mL) again with water (2 × 300 mL), dried over anhydrous Na_2SO_4. The residue was filtered through silica using CH_2Cl_2 as eluent to give $TPPO_2$ (**5**) as a dark purple solid (0.74 g, 1.1 mmol, 80%). ^{1}H-NMR (400 MHz, $CDCl_3$) δ = 9.05 (s, 1H), 9.02 (m, 1H), 8.95 (m, 1H), 8.90 (m, 1H), 8.89 (m, 1H), 8.72 (m, 1H), 8.71 (m, 1H), 8.25 (m, 2H), 8.22–8.18 (m, 6H), 7.82–7.71 (m, 12H), −2.61 (br s, 2H). UV-vis: (CH_2Cl_2) λ_{max}: 426, 526, 666.

2-Amino-5,10,15,20-tetraphenylporpyrin, $TPPNH_2$ (**6**) [1]. $TPPNO_2$ (**5**) (0.293 g, 0.44 mmol) was dissolved in dry degassed dichloromethane (25 mL), $Sn(II)Cl_2{\cdot}2H_2O$ (1.0 g, 4.4 mmol) and conc. HCl (3 mL) was added. The reaction was kept under N_2-atmand left to stir in the dark at room temperature for 3.5 days. Dichloromethane (60 mL) and water (60 mL) was added and the phases separated. The organic phase was washed with water (2 × 100 mL), 5% $NaHCO_3$ solution (2 × 100 mL) again with water (2 × 100 mL), dried over anhydrous Na_2SO_4. Evaporation of solvents gave crude $TPPNH_2$ (**6**) as a purple solid (0.285 g) which was used as such. ^{1}H-NMR (400 MHz, $CDCl_3$) δ = 8.80–8.76 (m, 4H), 8.73 (d, *J* = 4.7 Hz, 1H), 8.68 (d, *J* = 4.7 Hz, 1H), 8.51 (d, *J* = 4.7 Hz, 1H), 8.21–8.12 (m, 7H), 7.82–7.69 (m, 13H), 4.43 (br s, 2H, NH_2), −2.68 (br s, 2H, NH).

Methyl 4-bromobenzoate (**8**) [3]. Trimethyl orthoacetate (16 mL, 0.12 mol) was added to 4-bromobenzoic acid **7** (3 g, 14.9 mmol) in a 10–20 mL Biotech microwave vial and heated with stirring for 1 h at 110 °C. The mixture was diluted with ethyl acetate and washed with water (3 × 30 mL). The combined aqueous phases were extracted with ethyl acetate and the combined organic phases were dried over

Na_2SO_4. Evaporation of solvents gave a crude product that was purified by column chromatography (pentane/EtOAc 5:1) yielding ester **8** as light beige solid (4.6 g, 21.4 mmol, 72%). R_f = 0.83 (*n*-pentane/EtOAc = 5:1). ^{1}H-NMR (400 MHz, $CDCl_3$) δ = 7.92 (AA′BB′, 2H, Ar-H), 7.60 (AA′BB′, 2H, Ar-H), 3.93 (s, 3H, OCH_3).

Methyl 4-(2-trimethylsilylethynyl)benzoate (**9**) [4]. Methyl 4-bromobenzoate (**8**) (1.5 g, 7 mmol), PPh_3 (0.37 g, 1.4 mmol), $Pd(PPh_3)_2Cl_2$ (0.3 g, 0.42 mmol), CuI (0.08 g, 0.42 mmol), trimethylsilyl acetylene (1.1 mL, 7.7 mmol), Et_2NH (10 mL, 98 mmol) and DMF (4 mL) was added to a 10–20 mL Biotech microwave vial. The mixture was heated by microwave irradiation to 120 °C for 25 min under N_2 atm. and stirring. The reaction mixture was diluted with diethyl ether and treated with 1 M HCl. The phases were separated and the aqueous phase washed with diethyl ether (3 × 30 mL). The combined organic phases were washed with saturated $NaHCO_3$ solution, water and brine. Solvents were evaporated to give crude black oil that was treated with pentane and filtered through celite. Evaporation of the filtrate gave ester **9** with high purity as bright orange solid (1.47 g, 6.33 mmol, 98%). R_f = 0.71 (n-pentane/EtOAc = 5:1). ^{1}H-NMR (400 MHz, $CDCl_3$) δ = 7.99 (AA′BB′, 2H, Ar-H), 7.54 (AA′BB′, 2H, Ar-H), 3.93 (s, 3H, OCH_3), 0.28 (s, 9H, $Si(CH_3)_3$).

Methyl 4-(ethynyl)benzoate (**10**) [5]. Methyl 4-(2-trimethylsilylethynyl)benzoate (**9**) (3 g, 12.9 mmol) was dissolved in 20 mL THF and cooled to −20 °C with acetone/dry ice. TBAF (1 M solution, 15 mL) was added during stirring. After 3 h, water (50 mL) was added and the phases were separated. The aqueous phase was washed with diethyl ether (3 × 50 mL) and the organic phases combined, washed with brine and dried over $MgSO_4$. Solvents were evaporated and the crude product was purified by column chromatography (pentane/EtOAc 5:1) to give ester **10** as dark yellow solid (1.9 g, 11.9 mmol, 90%). R_f = 0.90 (n-pentane/EtOAc = 4:1). ^{1}H-NMR (400 MHz, $CDCl_3$) δ = 8.01 (AA′BB′, 2H, Ar-H), 7.57 (AA′BB′, 2H, Ar-H), 3.93 (s, 3H, OCH_3), 3.24 (s, 1H, CH).

Ethyl 3-oxoindane-5-carboxylate (**15**) [6]. A solution of 1 g (5.68 mmol) of 3-oxoindane-5-carboxylic acid **14** in 100 mL ethanol and concentrated 12 M HCl (0.47 mL) was added and the mixture was stirred overnight under reflux. The solvent was evaporated which yielded **15** as a white solid. Yield: 1.10 g (5.39 mmol, 95%). R_f = 0.6 (n-pentane/dichloromethane/EtOAc = 3:1:1). ^{1}H-NMR (500 MHz, $CDCl_3$) δ = 8.43 (1H, m, Ar-H), 8.28 (1H, dd, *J* = 1.7, 8.0 Hz, Ar-H), 7.56 (1H, ddd, *J* = 0.9, 1.7, 8.0 Hz, Ar-H), 4.40 (2H, q, *J* = 7.1 Hz, OCH_2), 3.21 (2H, m, CH_2), 2.76 (2H, m, CH_2), 1.41 (3H, t, *J* = 7.1 Hz, OCH_2CH_3). ^{13}C-NMR (100.6 MHz, $CDCl_3$) δ = 206.1, 165.9, 159.5, 137.4, 135.5, 130.3, 126.9, 125.3, 61.5, 36.6, 26.2, 14.4; IR 1710 [ν (C=O)] cm^{-1}; *m/z* (EI-MS) 204 $[M + H]^+$.

L-Lysine methyl ester (**23**) [7,8]. Lysine hydrochloride (2 g, 10.95 mmol) was weighed into a round bottom flask together with 80 mL methanol. The resulting suspension was then cooled to 0 °C on ice, before dropwise addition of thionyl chloride 8 mL (110 mmol). Upon addition the reaction mixture turned homogenous. Subsequently, the flask was fitted with a reflux condenser and heated to reflux overnight. After allowing the mixture to reach room temperature, the solvent was removed under reduced pressure. The crude solid was then placed under high vacuum overnight to afford lysine methyl ester dihydrochloride as a white solid (2.3 g, 9.83 mmol, 96%). ^{1}H-NMR (300 MHz, CD_3OD) δ = 4.08 (t, *J* = 6.5 Hz, 1H), 3.85 (s, 3H), 2.96 (m, 2H), 2.07–1.87 (m, 2H), 1.78–1.68 (m, 2H), 1.65–1.45 (m, 2H). ^{13}C-NMR (100.6 MHz, DMSO-d6) δ = 169.9, 52.8, 51.6, 38.2, 29.3, 26.2, 21.2. APCI-MS: *m/z* calcd. for $C_7H_{16}N_2O_2$, $[M + H]^+$: 161.1; found: 161.1.

Compound **23** in free amine form was afforded from its corresponding dihydrochloride through treatment with Amblerite IRA-400 resin (chloride) [9,10]. The resin (1.5 cm^3) was rinsed with water (20 mL), NaOH (20 mL) and again with water (20 mL) before testing the flow through for residual Cl^- with $AgNO_3$. Once complete conversion to the OH^- form was established, the resin was washed with methanol (10 mL) before dissolving **23**·$(HCl)_2$ (15 mg, 0.064 mmol) in methanol (1 mL) and adding it to the resin. The mixture was left to stir for 30 min before filtration and removal of solvent which yielded **23** as a colorless solid (10 mg, 0.062 mmol, 97%). ^{1}H-NMR (300 MHz, CD_3OD) δ = 3.71 (s, 3H), 3.44 (dd, *J* = 7.0, 5. 9 Hz, 1H), 2.64 (m, 2H), 1.78–1.53 (m, 2H), 1.53–1.32 (m, 4H).

L-Tryptophan methyl ester (**24**) [7]. Following the same procedure as for **23**, *L*-tryptophan methyl ester hydrochloride (2.7 g, 9.27 mmol, 95%) was obtained from *L*-tryptophan (2 g, 9.79 mmol). ^{1}H-NMR (300 MHz, CD_3OD- d_6) δ = 7.53 (m, 1H), 7.37 (m, 1H), 7.19 (m 1H), 7.14 (m, 1H), 7.07 (m, 1H), 4.32 (m, 1H), 3.80 (s, 3H), 3.41–2.92 (m, 2H). ^{13}C-NMR (100.6 MHz, DMSO- d_6) δ = 169.8, 136.2, 126.9, 125.0, 121.2, 118.6, 118.0, 111.6, 106.3, 52.7, 26.1 APCI-MS: *m/z* calcd. for $C_{12}H_{14}N_2O_2$, $[M + H]^+$: 219.1; found: 219.1.

Compound **24** in free amine form was prepared from its corresponding hydrochloride salt (17 mg, 0.059 mmol) according to the procedure described above for **23** as colorless solid (10 mg, 0.046 mmol, 78%). ^{1}H-NMR (300 MHz, CD_3OD-d_6) δ = 7.51 (m, 1H), 7.33 (m, 1H), 7.09 (m 1H), 7.08 (m, 1H), 7.00 (m, 1H), 3.77 (m, 1H), 3.64 (s, 3H), 3.22–3.07 (m, 2H).

2. X-ray Crystallography

*2.1. Z-**16***

Table S1. Crystal data and structure refinement details.

Identification code	**2012acc0104** (HH012C)	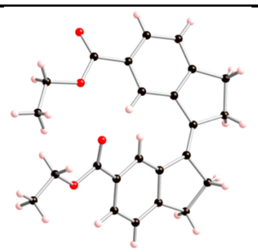
Empirical formula	$C_{24}H_{24}O_4$	
Formula weight	376.43	
Temperature	100(2) K	
Wavelength	0.71075 Å	
Crystal system	Monoclinic	
Space group	$P2_1/c$	
Unit cell dimensions	a = 12.074(3) Å	
	b = 21.114(4) Å	β = 93.953(3)°
	c = 7.6388(16) Å	
Volume	1942.8(7) Å^3	
Z	4	
Density (calculated)	1.287 Mg/m^3	
Absorption coefficient	0.087 mm^{-1}	
F(000)	800	
Crystal	Slab; Yellow	
Crystal size	0.29 × 0.09 × 0.03 mm^3	
θ range for data collection	3.21–27.48°	
Index ranges	$-15 \le h \le 12$, $-27 \le k \le 24$, $-9 \le l \le 9$	
Reflections collected	12601	
Independent reflections	4432 [R_{int} = 0.0251]	
Completeness to θ = 27.48°	99.5%	
Absorption correction	Semi–empirical from equivalents	
Max. and min. transmission	0.9974 and 0.9753	
Refinement method	Full-matrix least-squares on F^2	
Data/restraints/parameters	4432/0/255	
Goodness-of-fit on F^2	1.058	
Final R indices [$F^2 > 2\sigma(F^2)$]	$R1$ = 0.0443, $wR2$ = 0.1086	
R indices (all data)	$R1$ = 0.0520, $wR2$ = 0.1133	
Largest diff. peak and hole	0.296 and −0.184 e·Å^{-3}	

Diffractometer: *Rigaku AFC12* goniometer equipped with an enhanced sensitivity (HG) *Saturn724* + detector mounted at the window of an *FR-E+ SuperBright* molybdenum rotating anode generator with HF *Varimax* optics (100 μm focus). **Cell determination, Data collection, Data reduction and cell refinement & Absorption correction**: CrystalClear-SM Expert 2.0 r7 (Rigaku, 2011), **Structure solution**: SHELXS97 (G.M. Sheldrick, Acta Cryst. (1990) A**46** 467–473). **Structure refinement**: SHELXL97 (G.M. Sheldrick (1997), University of Göttingen, Germany). **Graphics:** CrystalMaker: a crystal and molecular structures program for Mac and Windows. CrystalMaker Software Ltd, Oxford, England (www.crystalmaker.com).

Special details: All hydrogen atoms were placed in ideal positions and refined using a riding model, methyl torsion angles were allowed to refine.

Table S2. Atomic coordinates [×10⁴], equivalent isotropic displacement parameters [Å^2 × 10^3] and site occupancy factors. U_{eq} is defined as one third of the trace of the orthogonalized U^{ij} tensor.

Atom	*x*	*y*	*z*	U_{eq}	*S.o.f.*
O1	706(1)	2994(1)	2374(1)	26(1)	1
O2	1610(1)	3923(1)	2390(2)	36(1)	1
O3	1593(1)	4626(1)	−2470(1)	26(1)	1
O4	1710(1)	5522(1)	−4041(1)	30(1)	1
C1	−1007(1)	2821(1)	3675(2)	38(1)	1
C2	−181(1)	3310(1)	3217(2)	36(1)	1
C3	1577(1)	3364(1)	2055(2)	22(1)	1
C4	2487(1)	3009(1)	1279(2)	18(1)	1
C5	2506(1)	2348(1)	1223(2)	20(1)	1
C6	3437(1)	2040(1)	683(2)	21(1)	1
C7	4331(1)	2391(1)	177(2)	19(1)	1
C8	4288(1)	3055(1)	101(2)	17(1)	1
C9	3369(1)	3362(1)	706(2)	17(1)	1
C10	5450(1)	2154(1)	−292(2)	22(1)	1
C11	6173(1)	2756(1)	−227(2)	22(1)	1
C12	5350(1)	3303(1)	−465(2)	18(1)	1
C13	5640(1)	3861(1)	−1149(2)	19(1)	1
C14	6842(1)	4018(1)	−1482(2)	24(1)	1
C15	6865(1)	4726(1)	−1970(2)	23(1)	1
C16	5659(1)	4896(1)	−2319(2)	20(1)	1
C17	4965(1)	4406(1)	−1800(2)	18(1)	1
C18	5212(1)	5444(1)	−3087(2)	22(1)	1
C19	4069(1)	5506(1)	−3344(2)	22(1)	1
C20	3374(1)	5018(1)	−2856(2)	19(1)	1
C21	3818(1)	4467(1)	−2096(2)	17(1)	1
C22	2154(1)	5093(1)	−3200(2)	22(1)	1
C23	394(1)	4624(1)	−2823(2)	32(1)	1
C24	−42(1)	4089(1)	−1776(3)	50(1)	1

Table S3. Bond lengths (Å) and angles (°).

O1–C3	1.3459(15)	C17–C21	1.3943(17)	C12–C11–C10	104.89(10)
O1–C2	1.4500(15)	C18–C19	1.3858(19)	C13–C12–C8	132.71(11)
O2–C3	1.2085(16)	C19–C20	1.3954(17)	C13–C12–C11	121.64(11)
O3–C22	1.3410(15)	C20–C21	1.3904(17)	C8–C12–C11	105.57(10)
O3–C23	1.4536(16)	C20–C22	1.4876(18)	C12–C13–C17	131.42(11)
O4–C22	1.2126(16)	C23–C24	1.500(2)	C12–C13–C14	122.06(11)
C1–C2	1.4943(19)	C3–O1–C2	115.01(10)	C17–C13–C14	106.44(10)
C3–C4	1.4864(17)	C22–O3–C23	116.73(10)	C13–C14–C15	106.54(10)
C4–C9	1.3957(16)	O1–C2–C1	108.05(12)	C16–C15–C14	103.95(10)
C4–C5	1.3963(18)	O2–C3–O1	122.96(12)	C18–C16–C17	120.68(12)
C5–C6	1.3868(18)	O2–C3–C4	124.24(11)	C18–C16–C15	128.08(11)
C6–C7	1.3869(18)	O1–C3–C4	112.80(11)	C17–C16–C15	111.22(11)
C7–C8	1.4034(17)	C9–C4–C5	120.64(11)	C21–C17–C16	119.42(12)
C7–C10	1.5065(17)	C9–C4–C3	117.19(11)	C21–C17–C13	130.22(11)
C8–C9	1.3914(16)	C5–C4–C3	122.03(11)	C16–C17–C13	110.07(11)
C8–C12	1.4772(16)	C6–C5–C4	119.64(11)	C19–C18–C16	119.47(11)
C10–C11	1.5409(18)	C5–C6–C7	119.59(12)	C18–C19–C20	120.26(12)
C11–C12	1.5260(17)	C6–C7–C8	121.24(11)	C21–C20–C19	120.52(12)
C12–C13	1.3453(18)	C6–C7–C10	128.03(11)	C21–C20–C22	120.76(11)
C13–C17	1.4763(17)	C8–C7–C10	110.70(11)	C19–C20–C22	118.71(12)
C13–C14	1.5274(16)	C9–C8–C7	118.71(11)	C20–C21–C17	119.63(11)
C14–C15	1.5402(19)	C9–C8–C12	131.31(11)	O4–C22–O3	123.52(12)
C15–C16	1.5061(18)	C7–C8–C12	109.58(10)	O4–C22–C20	124.74(12)
C16–C18	1.3892(18)	C8–C9–C4	119.89(11)	O3–C22–C20	111.74(11)
C16–C17	1.4059(17)	C7–C10–C11	103.51(10)	O3–C23–C24	106.52(11)

Table S4. Anisotropic displacement parameters [Å^2 × 10^3]. The anisotropic displacement factor exponent takes the form: $-2\pi^2[h^2a^{*2}U^{11} + \cdots + 2\,h\,k\,a^*\,b^*\,U^{12}]$.

Atom	U^{11}	U^{22}	U^{33}	U^{23}	U^{13}	U^{12}
O1	18(1)	24(1)	35(1)	−1(1)	9(1)	−2(1)
O2	29(1)	21(1)	60(1)	−6(1)	22(1)	−2(1)
O3	15(1)	29(1)	35(1)	10(1)	1(1)	1(1)
O4	27(1)	25(1)	38(1)	7(1)	3(1)	7(1)
C1	27(1)	44(1)	45(1)	1(1)	16(1)	−6(1)
C2	24(1)	34(1)	52(1)	−1(1)	19(1)	2(1)
C3	19(1)	22(1)	26(1)	2(1)	5(1)	−1(1)
C4	17(1)	20(1)	18(1)	1(1)	0(1)	1(1)
C5	23(1)	20(1)	18(1)	1(1)	2(1)	−4(1)
C6	29(1)	15(1)	19(1)	1(1)	1(1)	1(1)
C7	22(1)	20(1)	14(1)	0(1)	−1(1)	4(1)
C8	17(1)	19(1)	14(1)	0(1)	−2(1)	1(1)
C9	17(1)	16(1)	17(1)	0(1)	0(1)	0(1)
C10	25(1)	22(1)	20(1)	1(1)	3(1)	9(1)
C11	18(1)	27(1)	22(1)	−2(1)	0(1)	6(1)
C12	14(1)	22(1)	17(1)	−4(1)	0(1)	3(1)
C13	13(1)	24(1)	18(1)	−5(1)	2(1)	−1(1)
C14	14(1)	30(1)	27(1)	−6(1)	4(1)	−2(1)
C15	18(1)	31(1)	22(1)	−4(1)	4(1)	−7(1)
C16	19(1)	24(1)	17(1)	−6(1)	3(1)	−6(1)
C17	17(1)	19(1)	16(1)	−4(1)	4(1)	−2(1)
C18	27(1)	21(1)	19(1)	−3(1)	3(1)	−8(1)
C19	28(1)	17(1)	20(1)	−1(1)	2(1)	−2(1)
C20	20(1)	18(1)	19(1)	−4(1)	3(1)	0(1)
C21	17(1)	17(1)	18(1)	−2(1)	4(1)	−2(1)
C22	23(1)	20(1)	23(1)	−1(1)	4(1)	3(1)
C23	15(1)	37(1)	45(1)	10(1)	−2(1)	3(1)
C24	17(1)	60(1)	72(1)	29(1)	4(1)	−1(1)

Table S5. Hydrogen coordinates [×10[4]] and isotropic displacement parameters [Å[2] × 10[3]].

Atom	*x*	*y*	*z*	U_{eq}	*S.o.f.*
H1A	−1295	2604	2605	57	1
H1B	−1620	3025	4234	57	1
H1C	−646	2512	4486	57	1
H2A	−541	3629	2413	43	1
H2B	120	3530	4291	43	1
H5	1885	2112	1554	24	1
H6	3462	1590	659	25	1
H9	3343	3812	728	20	1
H10A	5405	1964	−1479	27	1
H10B	5748	1836	567	27	1
H11A	6611	2790	914	27	1
H11B	6689	2753	−1179	27	1
H14A	7095	3757	−2452	28	1
H14B	7334	3936	−415	28	1
H15A	7212	4981	−991	28	1
H15B	7280	4794	−3027	28	1
H18	5685	5774	−3432	27	1
H19	3758	5882	−3854	26	1
H21	3341	4134	−1779	21	1
H23A	199	4559	−4090	39	1
H23B	74	5031	−2464	39	1
H24A	321	3693	−2087	74	1
H24B	−845	4051	−2035	74	1
H24C	114	4173	−521	74	1

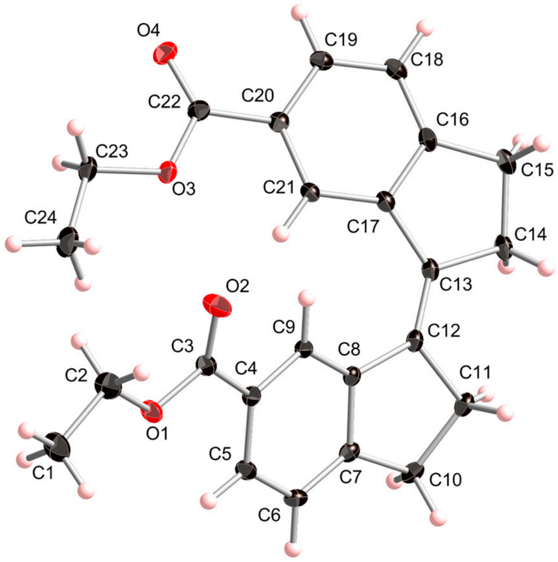

Figure S1. Thermal ellipsoids drawn at the 35% probability level.

2.2. *E*-**16**

Table S6. Crystal data and structure refinement details.

Identification code	**2012acc0103** (HH012T)	
Empirical formula	$C_{24}H_{24}O_4$	
Formula weight	376.43	
Temperature	100(2) K	
Wavelength	0.71075 Å	
Crystal system	Triclinic	
Space group	*P*–1	
Unit cell dimensions	a = 5.385(4) Å	α = 87.66(4)°
	b = 7.045(5) Å	β = 78.58(3)°
	c = 12.653(9) Å	γ = 78.56(3)°
Volume	461.2(6) $Å^3$	
Z	1 (Z′ = 0.5)	
Density (calculated)	1.355 Mg/m^3	
Absorption coefficient	0.091 mm^{-1}	
F(000)	200	
Crystal	Needle; Colourless	
Crystal size	0.13 × 0.08 × 0.03 mm^3	
θ range for data collection	2.95–25.02°	
Index ranges	$-6 \le h \le 6$, $-8 \le k \le 7$, $-15 \le l \le 15$	
Reflections collected	3499	
Independent reflections	1631 [R_{int} = 0.0260]	
Completeness to θ = 25.02°	99.5%	
Absorption correction	Semi–empirical from equivalents	
Max. and min. transmission	0.9973 and 0.9882	
Refinement method	Full-matrix least-squares on F^2	
Data/restraints/parameters	1631/0/128	
Goodness-of-fit on F^2	1.050	
Final R indices [$F^2 > 2\sigma(F^2)$]	$R1$ = 0.0413, $wR2$ = 0.0966	
R indices (all data)	$R1$ = 0.0479, $wR2$ = 0.1009	
Largest diff. peak and hole	0.233 and −0.158 e·$Å^{-3}$	

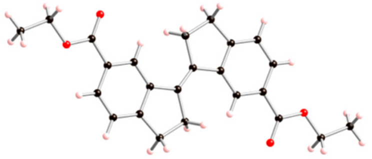

Diffractometer: *Rigaku AFC12* goniometer equipped with an enhanced sensitivity (HG) *Saturn724+* detector mounted at the window of an *FR-E+ SuperBright* molybdenum rotating anode generator with HF *Varimax* optics (100 µm focus). **Cell determination, Data collection, Data reduction and cell refinement & Absorption correction**: CrystalClear-SM Expert 2.0 r7 (Rigaku, 2011), **Structure solution**: SHELXS97 (G.M. Sheldrick, Acta Cryst. (1990) A**46** 467–473). S**tructure refinement**: SHELXL97 (G.M. Sheldrick (1997), University of Göttingen, Germany). **Graphics:** CrystalMaker: a crystal and molecular structures program for Mac and Windows. CrystalMaker Software Ltd, Oxford, England (www.crystalmaker.com).

Special details: All hydrogen atoms were placed in ideal positions and refined using a riding model, the methyl torsion angle was allowed to refine.

Table S7. Atomic coordinates [×10^4], equivalent isotropic displacement parameters [Å^2 × 10^3] and site occupancy factors. U_{eq} is defined as one third of the trace of the orthogonalized U^{ij} tensor.

Atom	*x*	*y*	*z*	U_{eq}	*S.o.f.*
O1	6877(2)	10373(1)	3687(1)	24(1)	1
O2	3766(2)	11007(1)	2703(1)	33(1)	1
C1	7793(3)	12553(2)	4879(1)	28(1)	1
C2	5897(3)	12254(2)	4198(1)	25(1)	1
C3	5631(3)	9933(2)	2943(1)	22(1)	1
C4	6760(3)	7991(2)	2455(1)	21(1)	1
C5	8859(3)	6786(2)	2786(1)	23(1)	1
C6	9828(3)	4968(2)	2328(1)	23(1)	1
C7	8693(3)	4365(2)	1542(1)	20(1)	1
C8	6627(3)	5574(2)	1175(1)	20(1)	1
C9	5652(3)	7404(2)	1647(1)	21(1)	1
C10	9442(3)	2446(2)	969(1)	21(1)	1
C11	7572(3)	2592(2)	172(1)	21(1)	1
C12	5891(3)	4619(2)	301(1)	20(1)	1

Table S8. Bond lengths (Å) and angles (°).

O1—C3	1.3373(18)	O2–C3–C4	124.06(14)
O1–C2	1.4529(19)	O1–C3–C4	112.79(12)
O2–C3	1.2137(18)	C9–C4–C5	120.66(14)
C1–C2	1.509(2)	C9–C4–C3	117.67(13)
C3–C4	1.488(2)	C5–C4–C3	121.67(14)
C4–C9	1.395(2)	C6–C5–C4	120.08(14)
C4–C5	1.397(2)	C7–C6–C5	119.34(14)
C5–C6	1.387(2)	C6–C7–C8	121.55(14)
C6–C7	1.386(2)	C6–C7–C10	127.01(13)
C7–C8	1.406(2)	C8–C7–C10	111.43(13)
C7–C10	1.505(2)	C9–C8–C7	118.60(14)
C8–C9	1.402(2)	C9–C8–C12	131.14(13)
C8–C12	1.473(2)	C7–C8–C12	110.25(13)
C10–C11	1.547(2)	C4–C9–C8	119.73(13)
C11–C12	1.528(2)	C7–C10–C11	104.46(12)
C12–C12^{i}	1.358(3)	C12–C11–C10	106.72(11)
C3–O1–C2	116.14(11)	C12^{i}–C12–C8	127.39(16)
O1–C2–C1	106.88(12)	C12^{i}–C12–C11	125.58(16)
O2–C3–O1	123.15(13)	C8–C12–C11	107.02(12)

Symmetry transformations used to generate equivalent atoms:
(i) −x+1, −y+1, −z

Table S9. Anisotropic displacement parameters [Å^2 × 10^3]. The anisotropic displacement factor exponent takes the form: $-2\pi^2[h^2a^{*2}U^{11} + \cdots + 2\,h\,k\,a^*\,b^*\,U^{12}]$.

Atom	U^{11}	U^{22}	U^{33}	U^{23}	U^{13}	U^{12}
O1	26(1)	23(1)	25(1)	−6(1)	−10(1)	0(1)
O2	32(1)	27(1)	38(1)	−9(1)	−17(1)	6(1)
C1	30(1)	26(1)	28(1)	−6(1)	−10(1)	−2(1)
C2	27(1)	20(1)	27(1)	−6(1)	−5(1)	0(1)
C3	21(1)	25(1)	20(1)	1(1)	−6(1)	−4(1)
C4	20(1)	22(1)	21(1)	0(1)	−4(1)	−4(1)
C5	23(1)	25(1)	21(1)	−2(1)	−7(1)	−4(1)
C6	22(1)	23(1)	23(1)	1(1)	−8(1)	0(1)
C7	19(1)	21(1)	19(1)	2(1)	−2(1)	−4(1)
C8	18(1)	22(1)	20(1)	2(1)	−4(1)	−4(1)
C9	20(1)	21(1)	22(1)	1(1)	−6(1)	−2(1)
C10	21(1)	20(1)	22(1)	1(1)	−5(1)	−2(1)
C11	20(1)	19(1)	24(1)	−1(1)	−6(1)	−3(1)
C12	19(1)	19(1)	21(1)	−1(1)	−3(1)	−5(1)

Table S10. Hydrogen coordinates [×10^4] and isotropic displacement parameters [Å^2 × 10^3].

Atom	*x*	*y*	*z*	U_{eq}	*S.o.f.*
H1A	7917	11523	5421	42	1
H1B	7200	13812	5239	42	1
H1C	9494	12518	4418	42	1
H2A	4168	12287	4654	30	1
H2B	5753	13286	3645	30	1
H5	9624	7213	3325	27	1
H6	11256	4145	2551	27	1
H9	4242	8240	1419	25	1
H10A	9229	1369	1485	26	1
H10B	11258	2236	578	26	1
H11A	8553	2394	−577	25	1
H11B	6487	1596	341	25	1

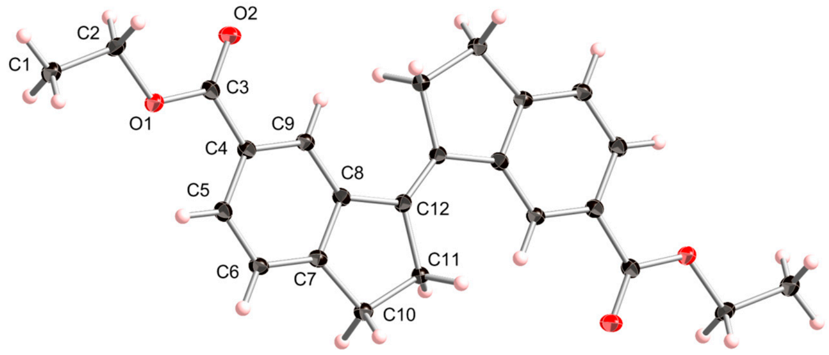

Figure S2. Thermal ellipsoids drawn at the 35% probability level.

3. NMR Spectra

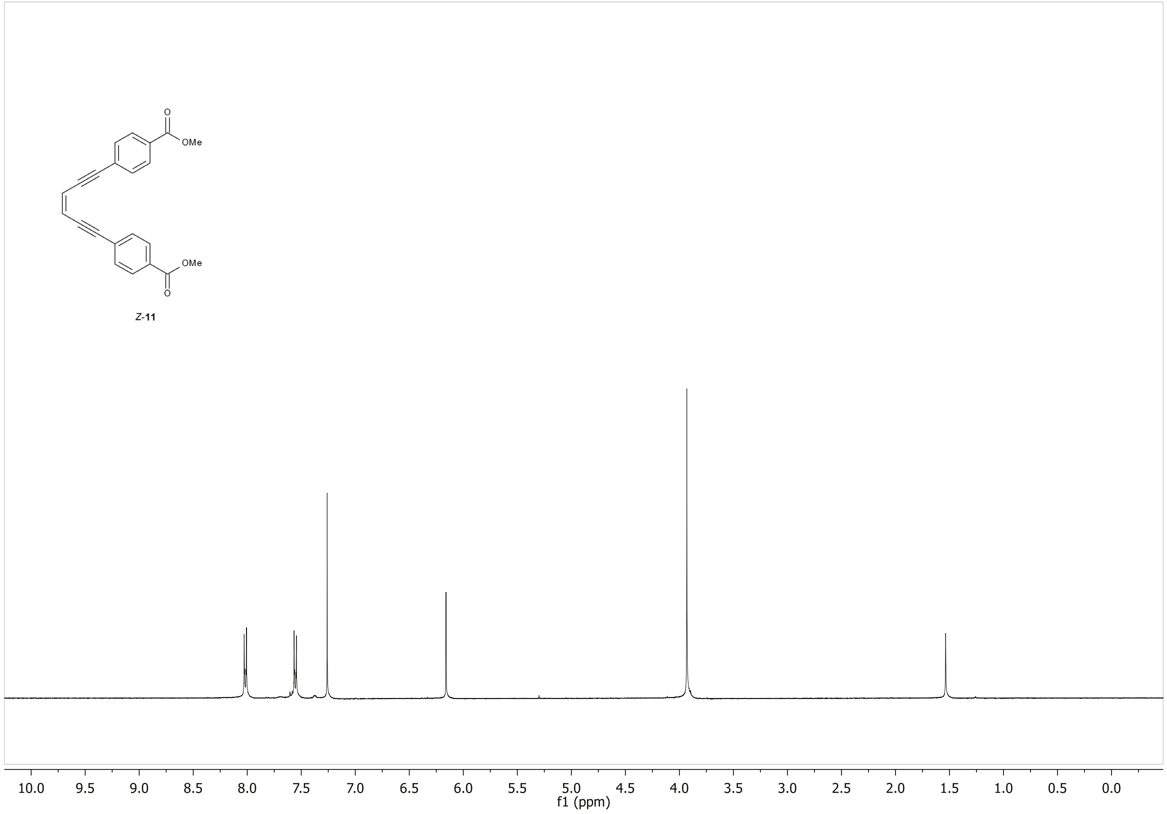

Figure S3. ^{1}H-NMR spectrum of *Z*-**11** (400 MHz, $CDCl_3$ solution).

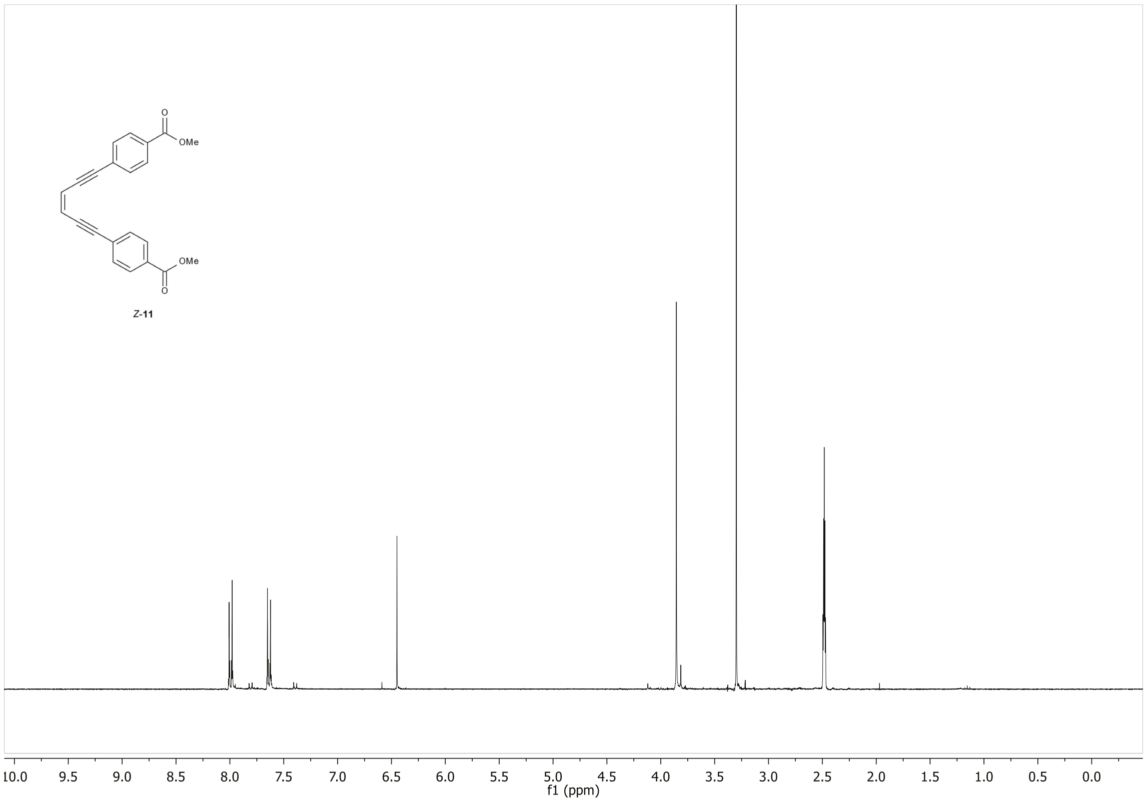

Figure S4. ^{1}H-NMR spectrum of *Z*-**11** (300 MHz, DMSO-d_6 solution).

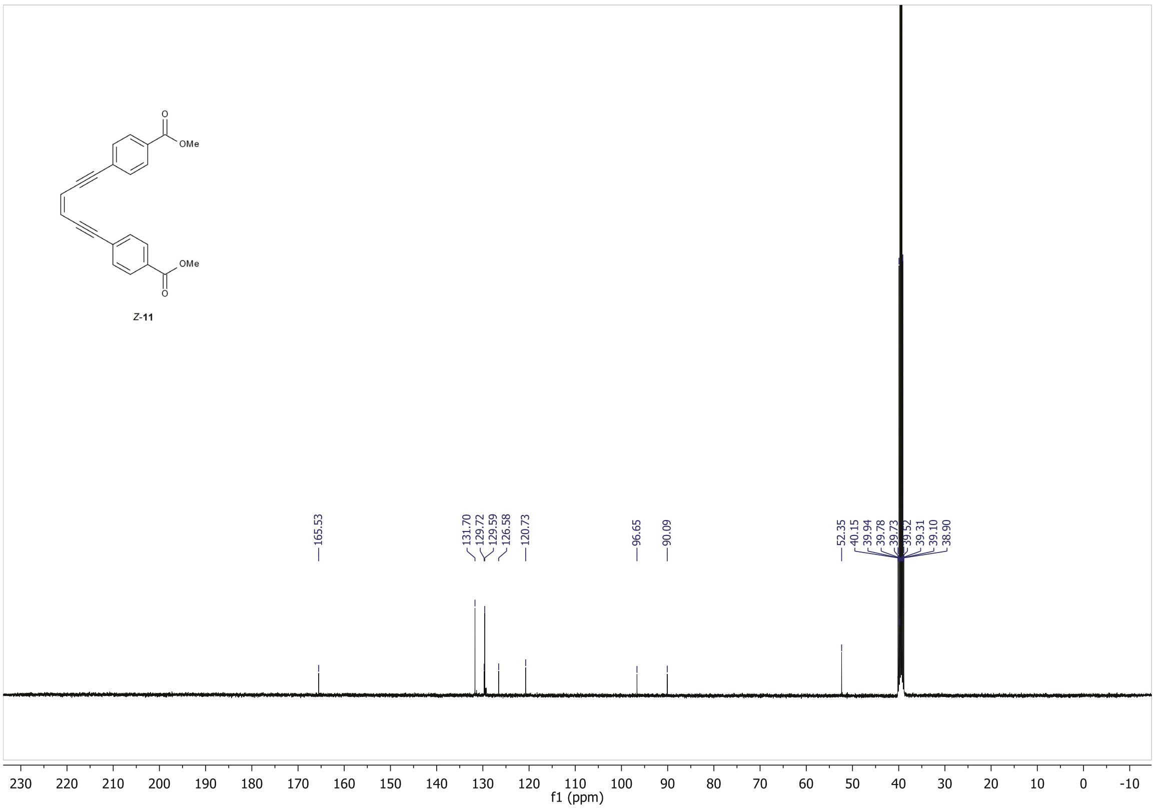

Figure S5. ^{13}C-NMR spectrum of *Z*-**11** (100.6 MHz, DMSO-d$_6$ solution).

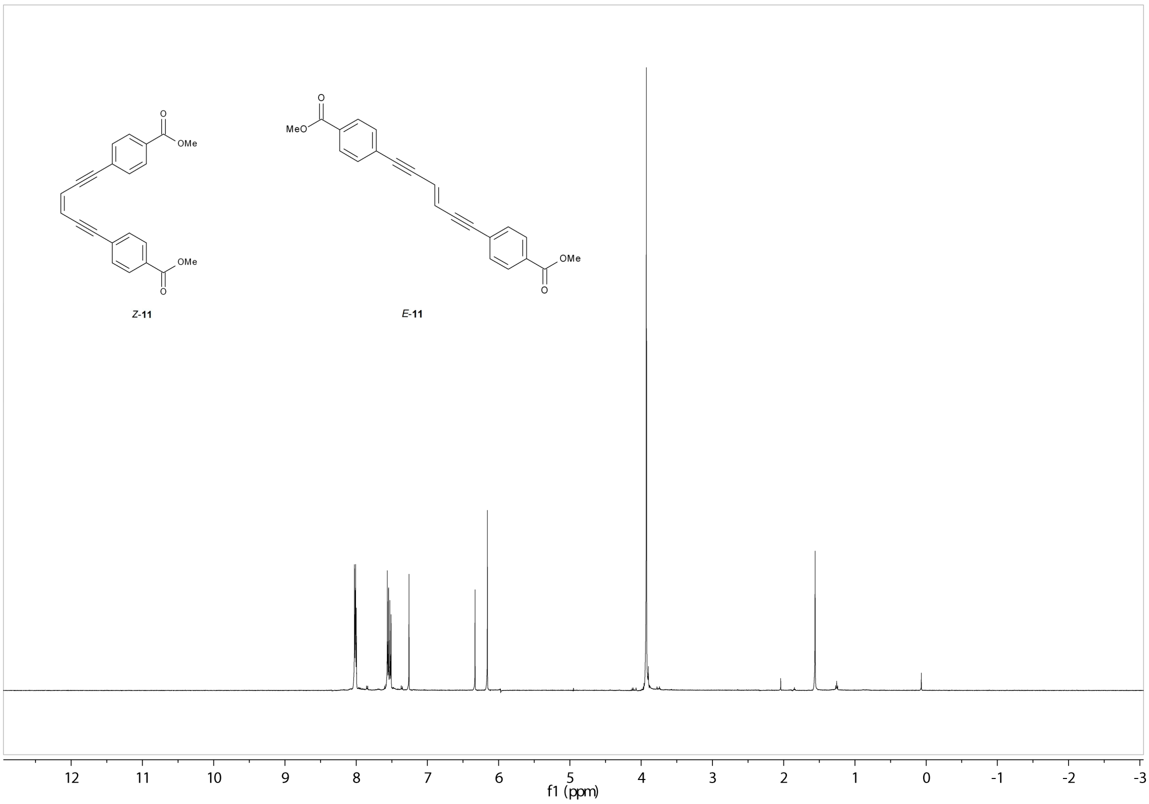

Figure S6. ^{1}H-NMR spectrum of *E*/*Z*-**11** mixture (400 MHz, DMSO-d$_6$ solution).

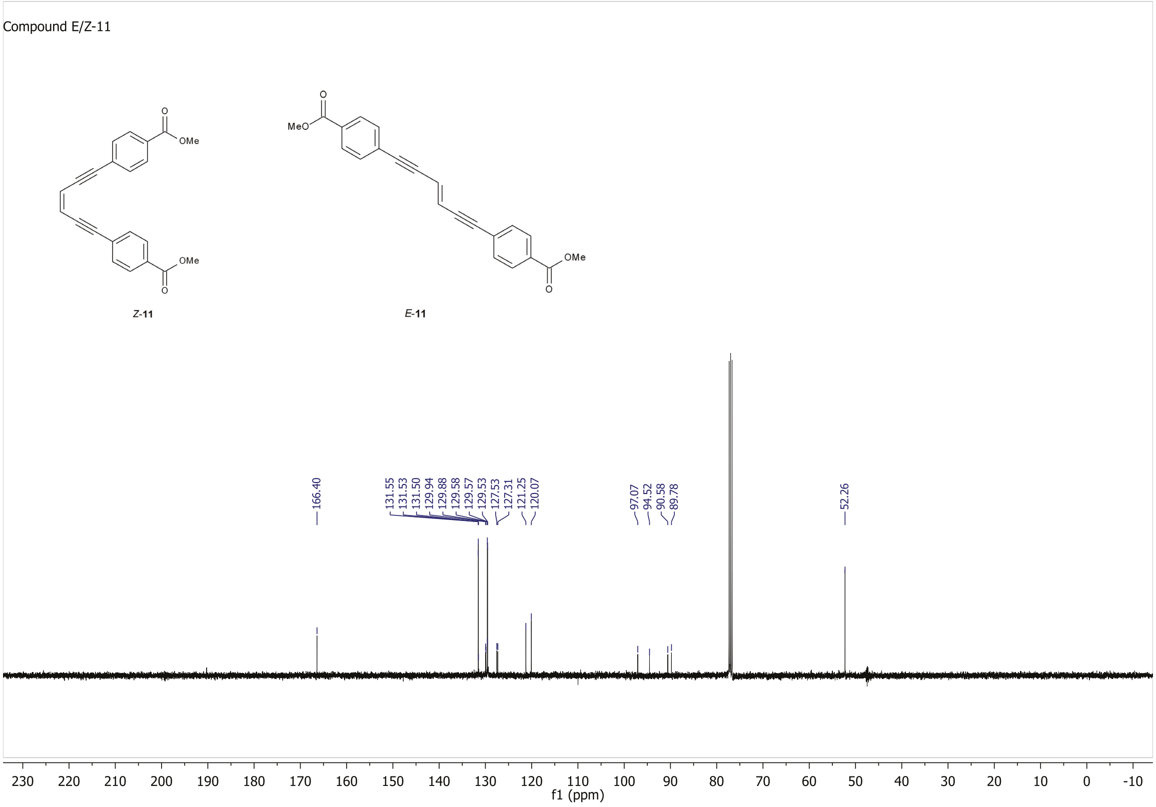

Figure S7. ^{13}C-NMR spectrum of *E*/*Z*-**11** mixture (100.6 MHz, $CDCl_3$ solution).

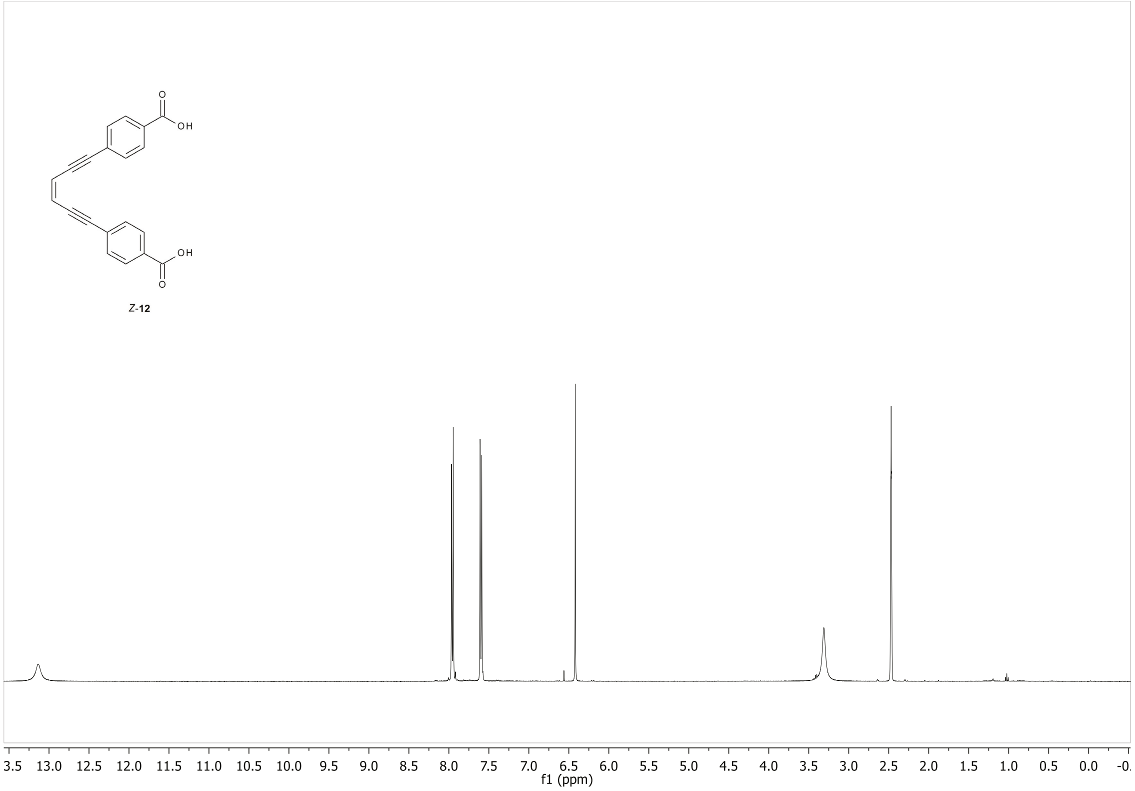

Figure S8. ^{1}H-NMR spectrum of *Z*-**12** (400 MHz, DMSO-d_6 solution).

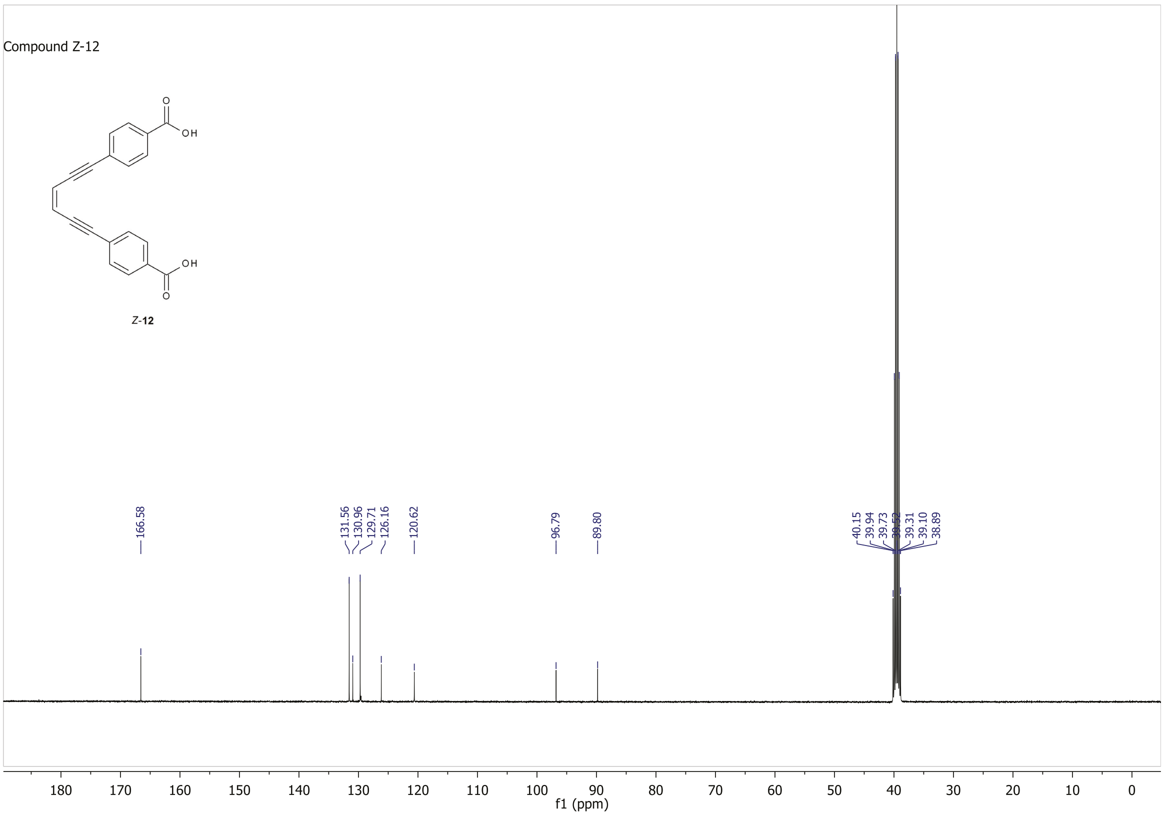

Figure S9. ^{13}C-NMR spectrum of *Z*-**12** (100.6 MHz, DMSO-d_6 solution).

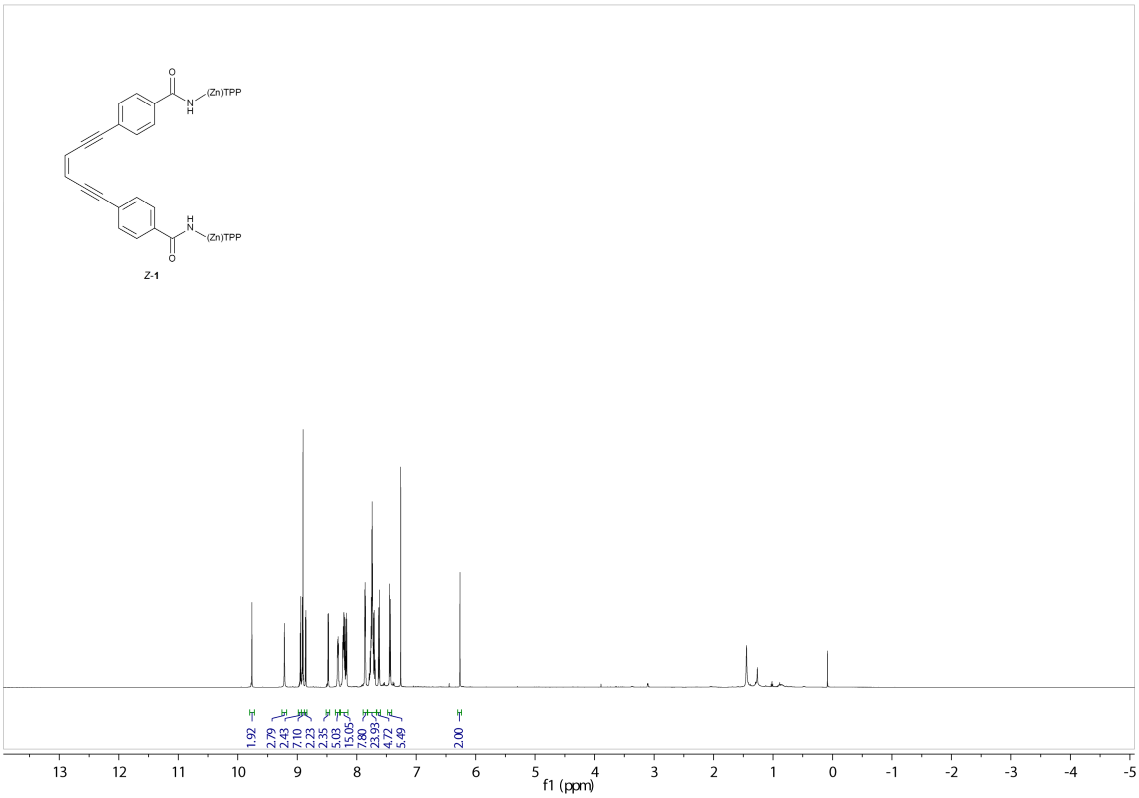

Figure S10. ^{1}H-NMR spectrum of *Z*-**1** (500 MHz, $CDCl_3$ solution).

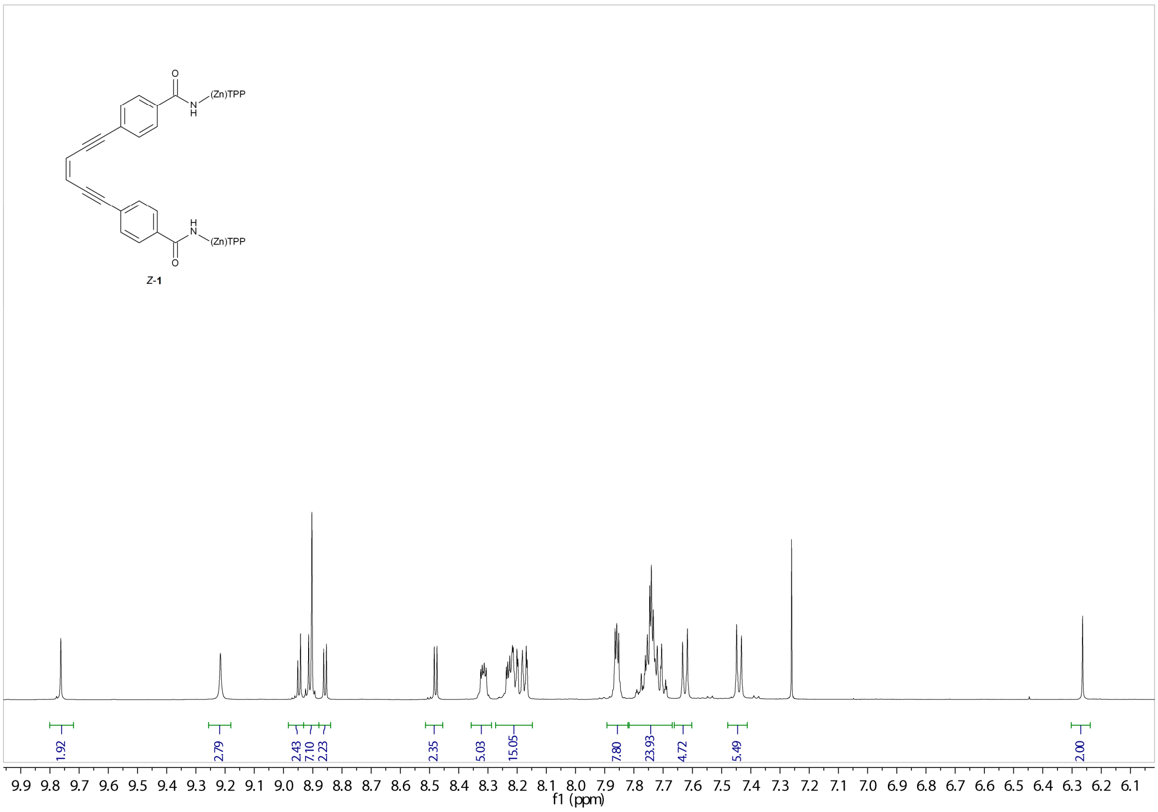

Figure S11. Expansion of ^{1}H-NMR spectrum of *Z*-**1** (500 MHz, $CDCl_3$ solution).

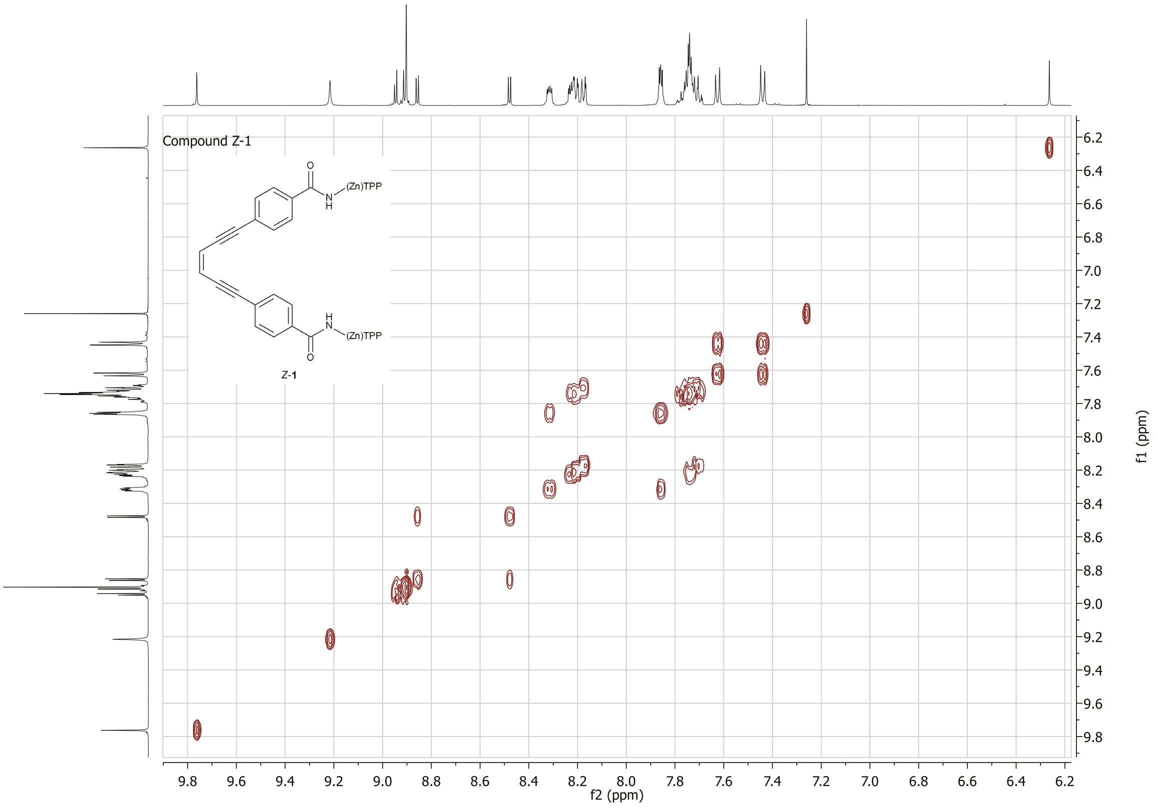

Figure S12. ^{1}H gCOSY spectrum of *Z*-**1** (500 MHz, $CDCl_3$ solution).

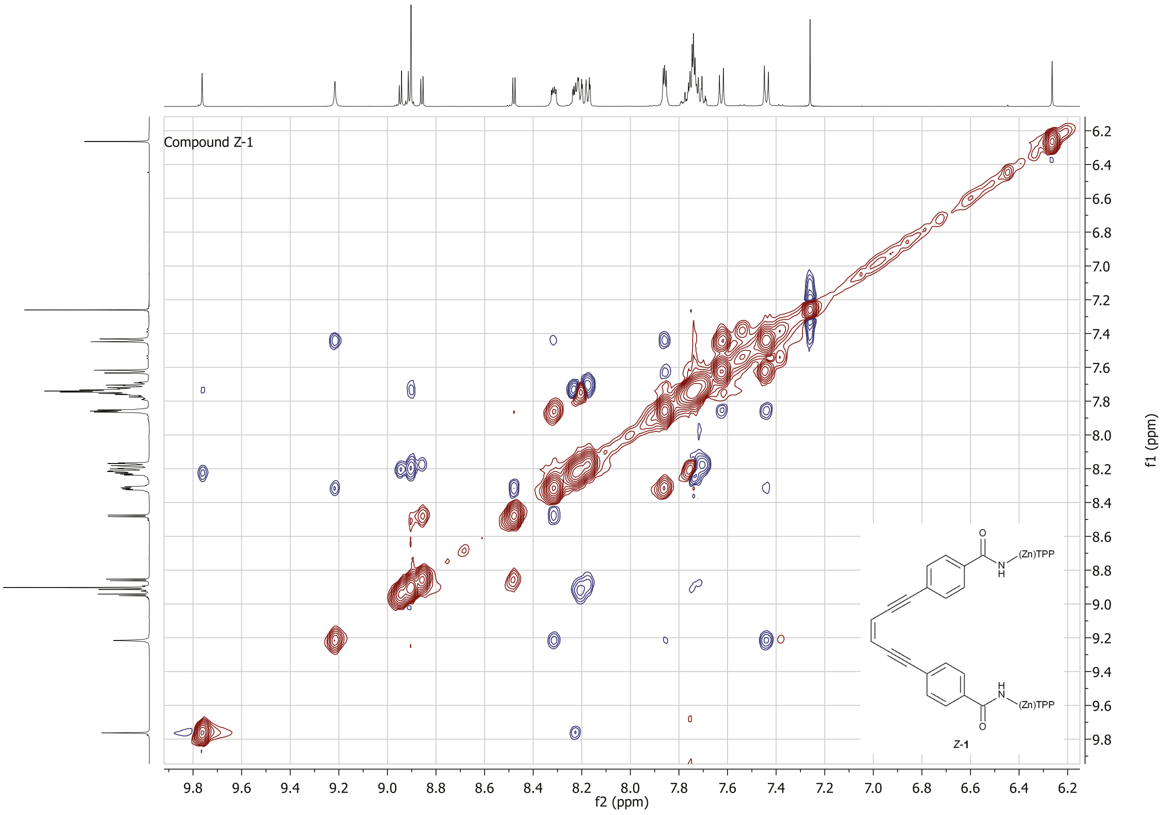

Figure S13. ^{1}H NOESY spectrum of *Z*-**1** (500 MHz, $CDCl_3$ solution).

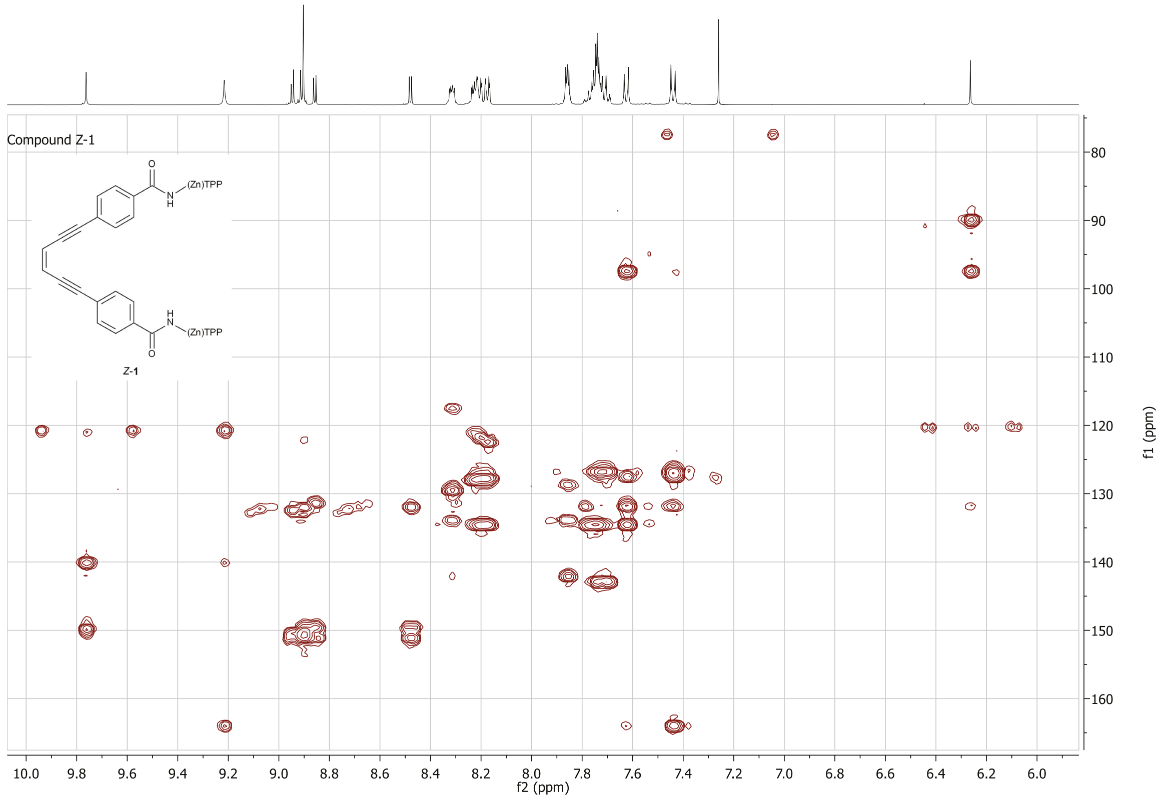

Figure S14. ^{1}H-^{13}C gHMBC spectrum of *Z*-**1** (500 MHz, $CDCl_3$ solution).

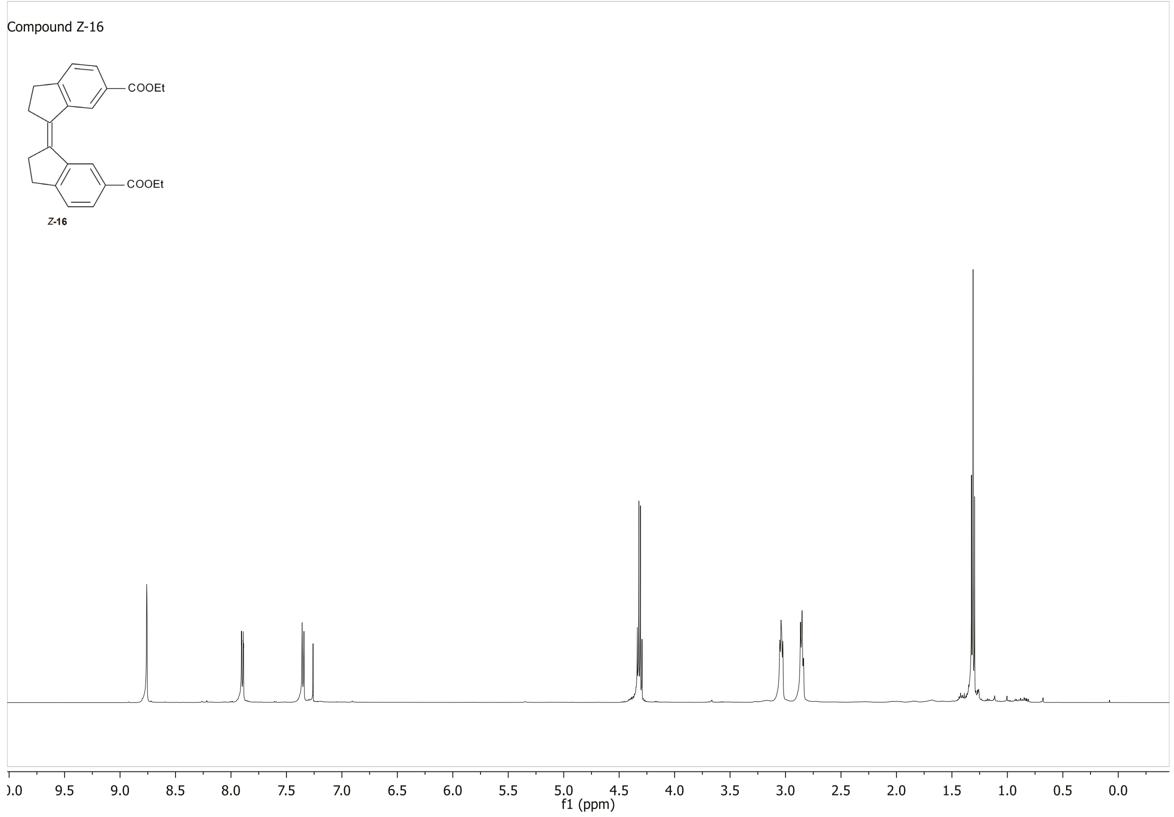

Figure S15. ^{1}H-NMR spectrum of *Z*-**16** (500 MHz, $CDCl_3$ solution).

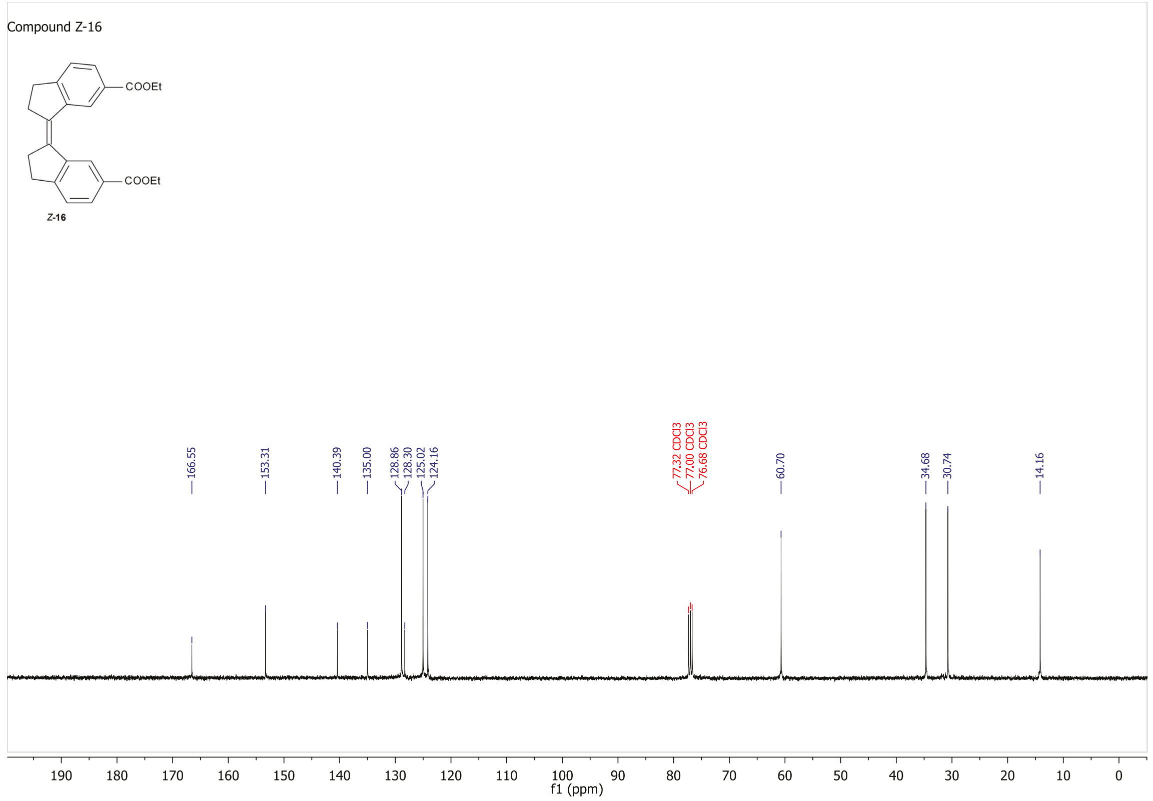

Figure S16. ^{13}C-NMR spectrum of *Z*-**16** (100.6 MHz, $CDCl_3$ solution).

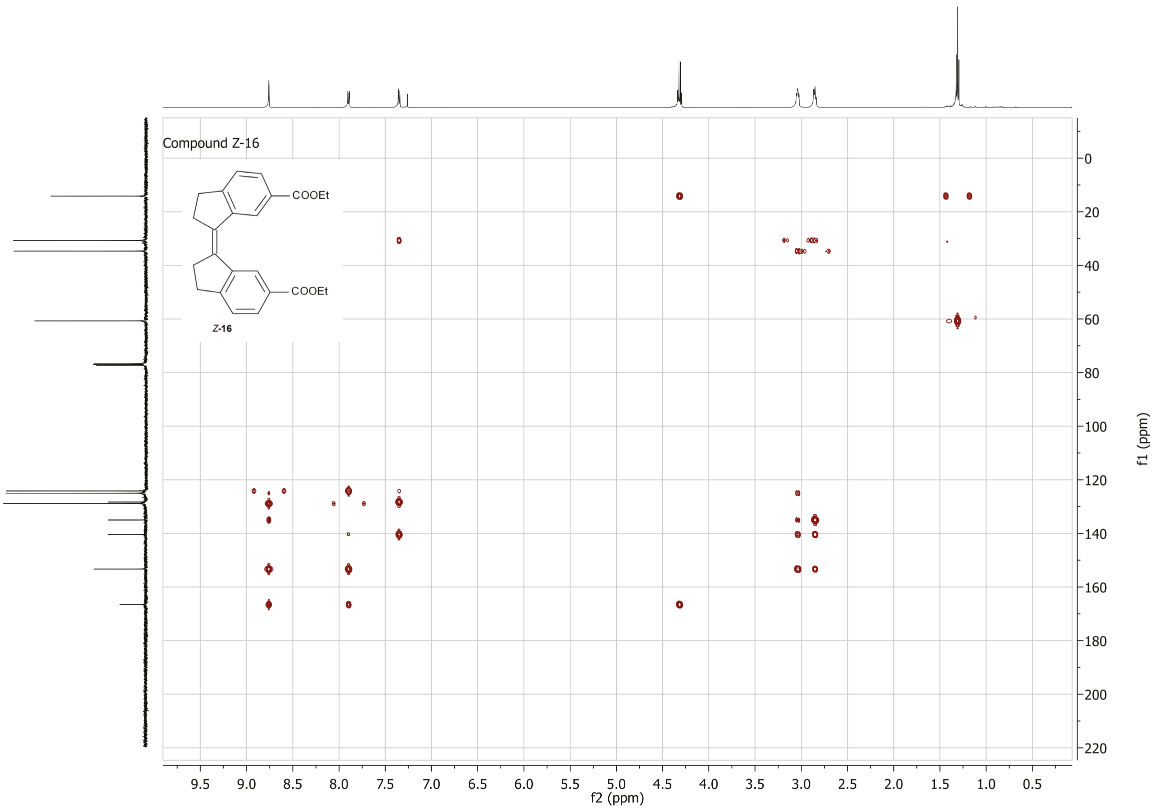

Figure S17. ^{1}H-^{13}C gHMBC spectrum of *Z*-**16** (500 MHz, $CDCl_3$ solution).

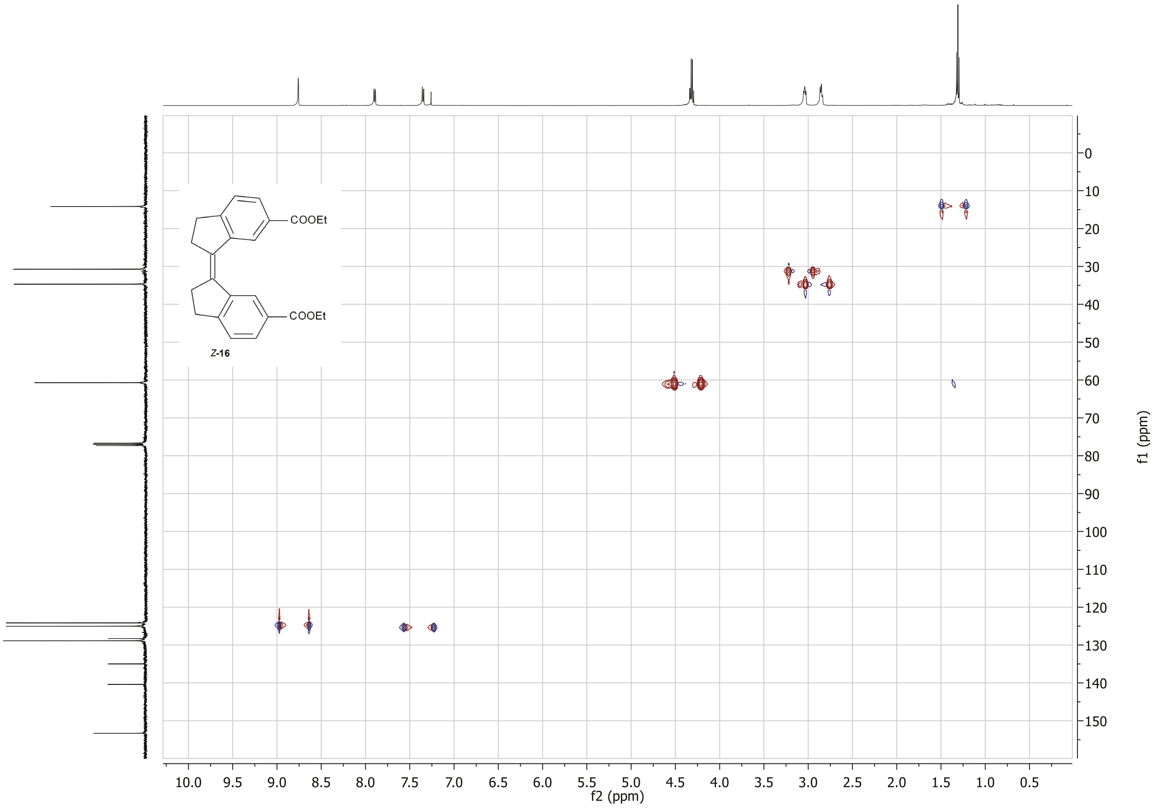

Figure S18. ^{1}H-^{13}C gHSQC spectrum of *Z*-**16** (500 MHz, $CDCl_3$ solution).

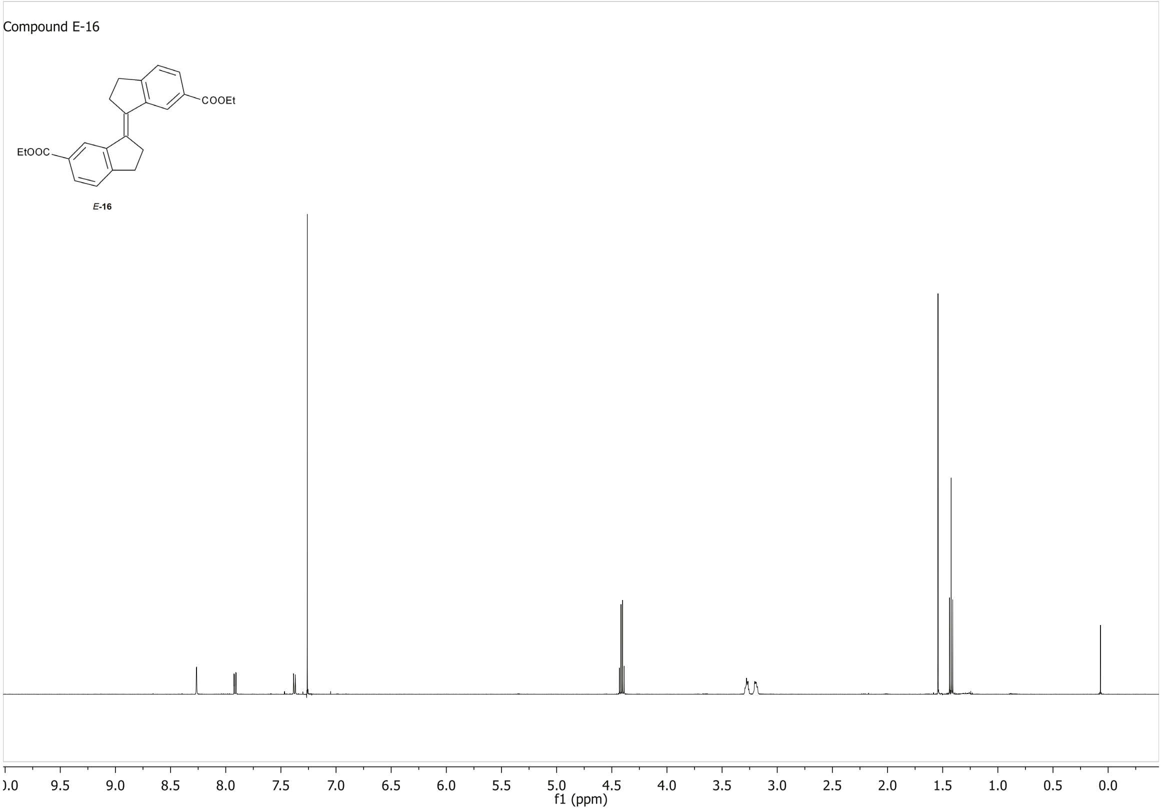

Figure S19. ^{1}H-NMR spectrum of *E*-**16** (500 MHz, $CDCl_3$ solution).

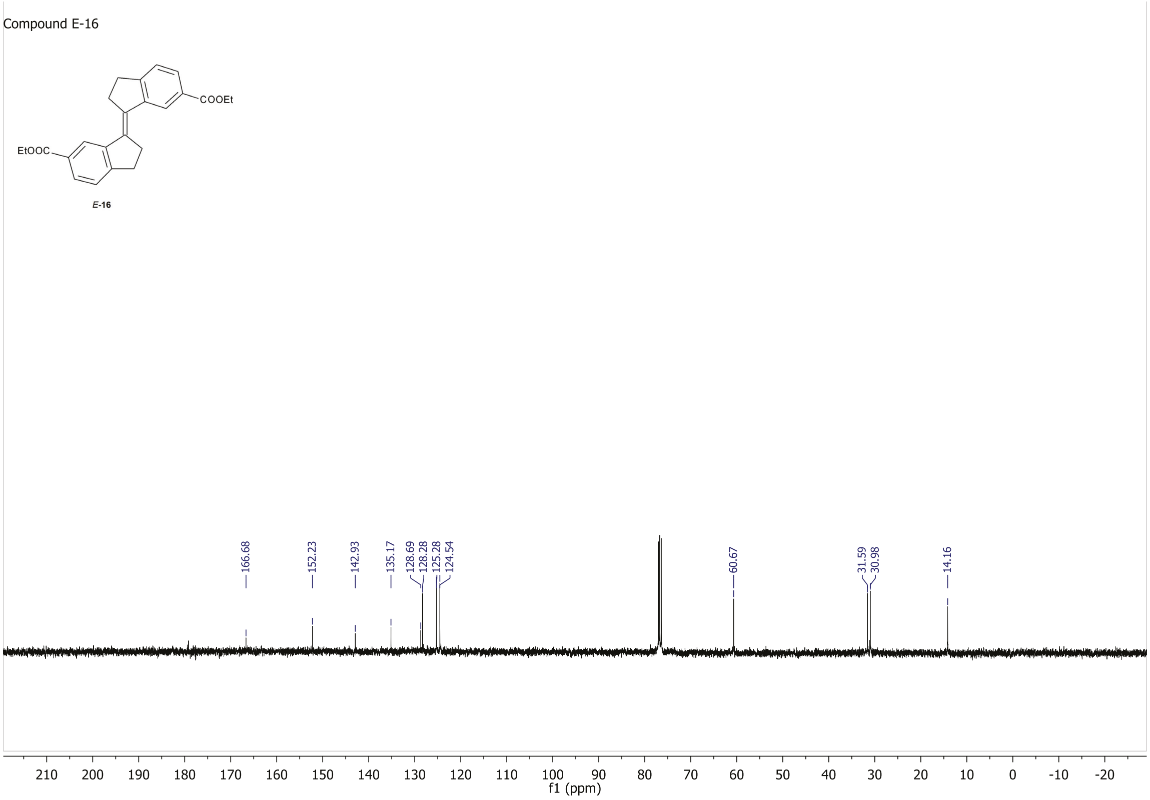

Figure S20. ^{13}C-NMR spectrum of *E*-**16** (100.6 MHz, $CDCl_3$ solution).

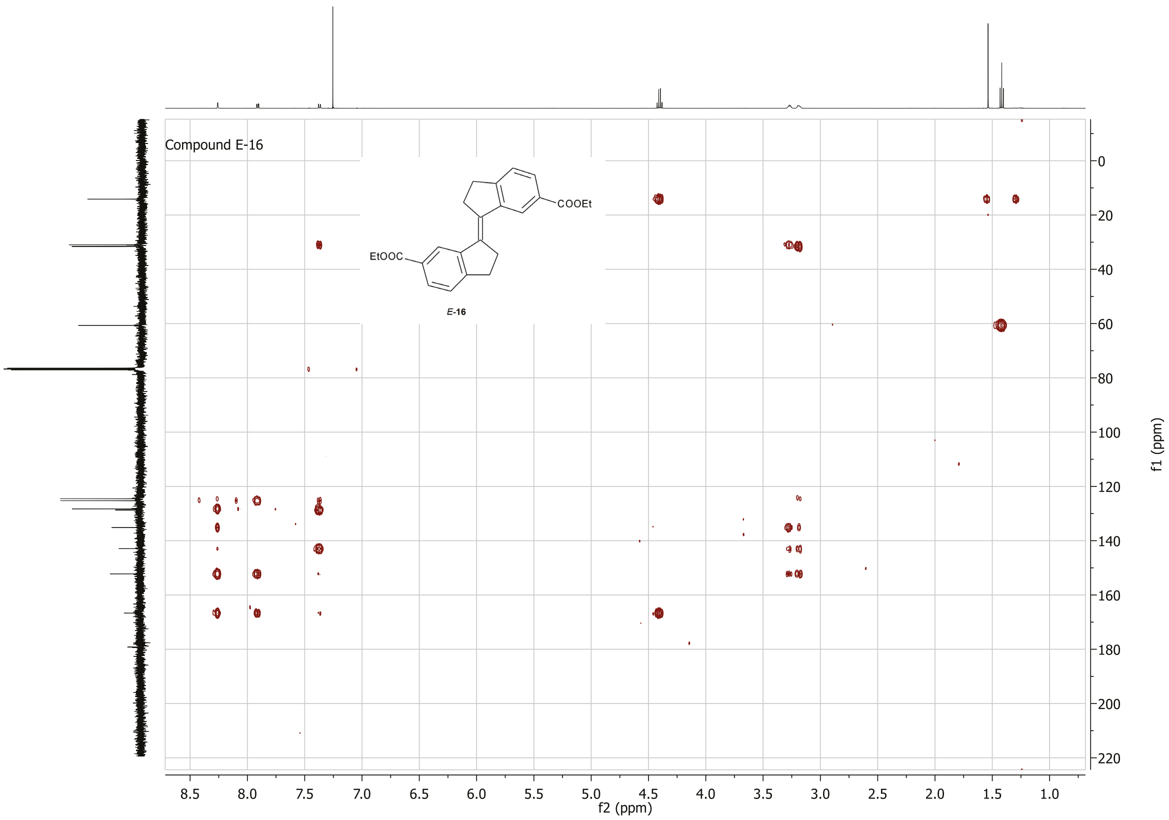

Figure S21. ^{1}H-^{13}C gHMBC spectrum of *E*-**16** (500 MHz, $CDCl_3$ solution).

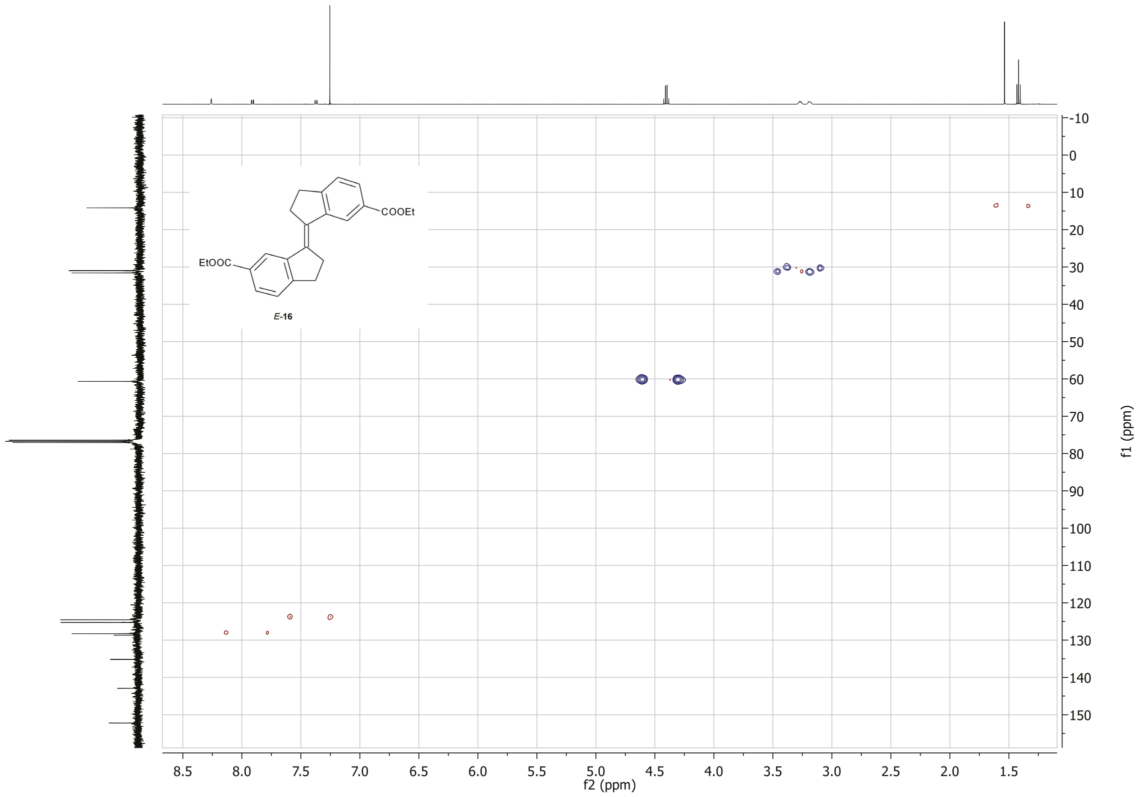

Figure S22. ^{1}H-^{13}C gHSQC spectrum of *E*-**16** (500 MHz, $CDCl_3$ solution).

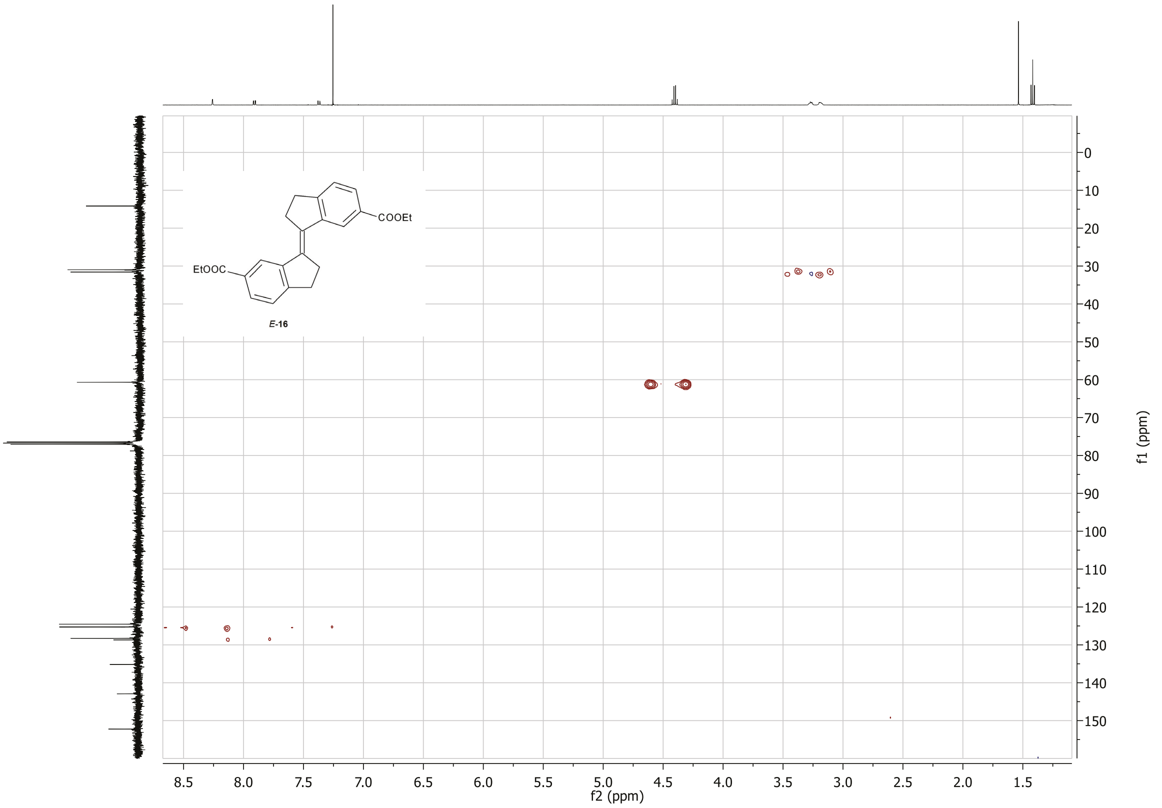

Figure S23. ^{1}H-^{13}C gHSQC spectrum of *E*-**16** (500 MHz, $CDCl_3$ solution).

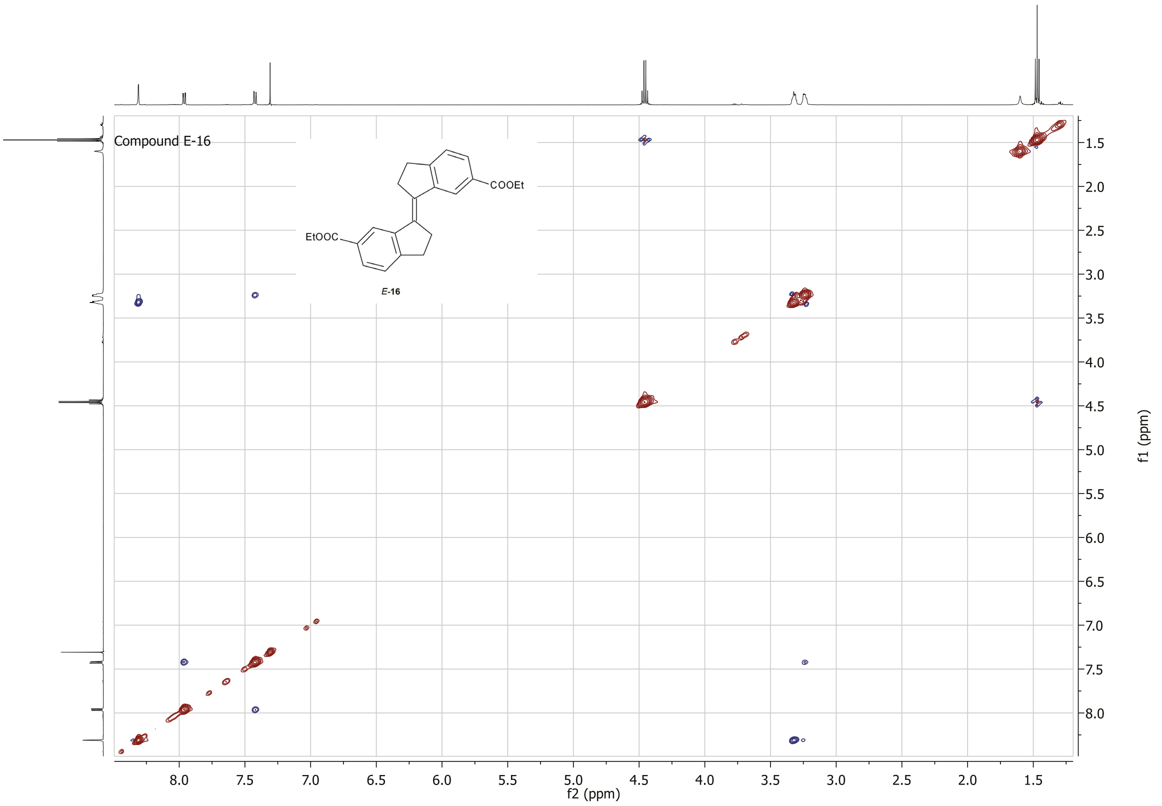

Figure S24. ^{1}H NOESY spectrum of *E*-**16** (500 MHz, $CDCl_3$ solution).

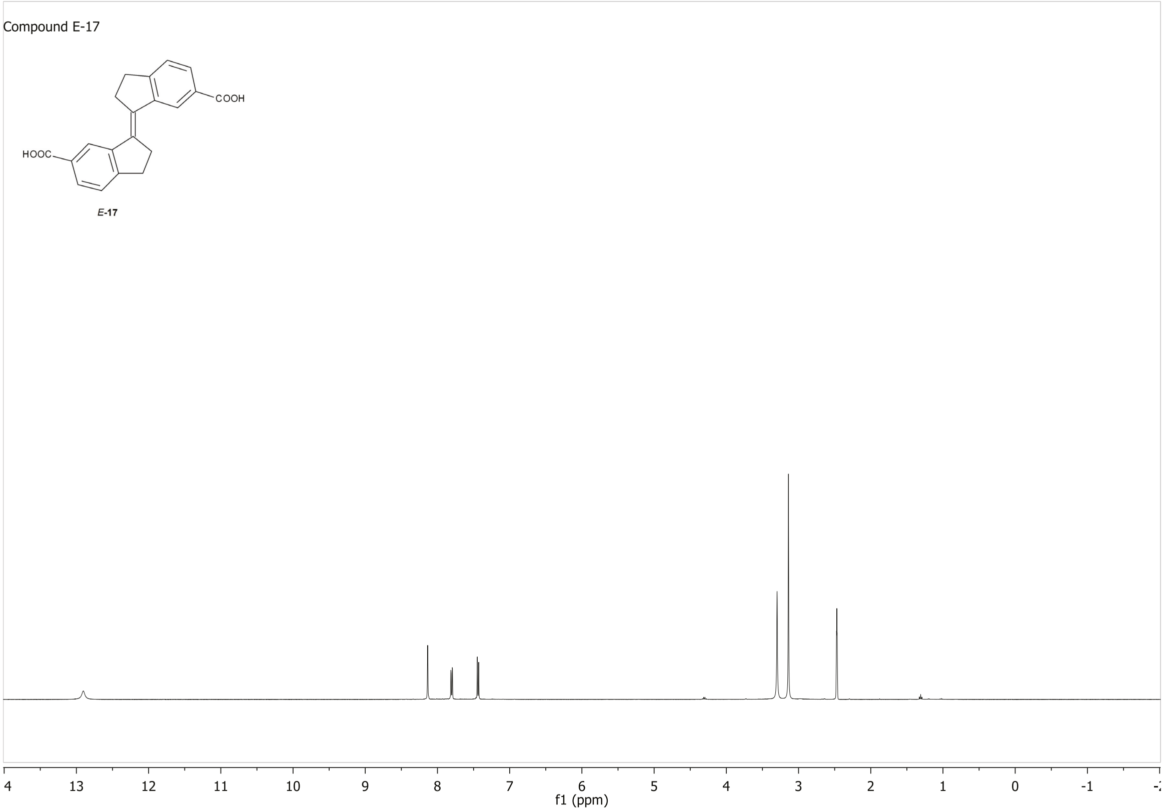

Figure S25. ^{1}H-NMR spectrum of *E*-**17** (400 MHz, DMSO-d$_6$ solution).

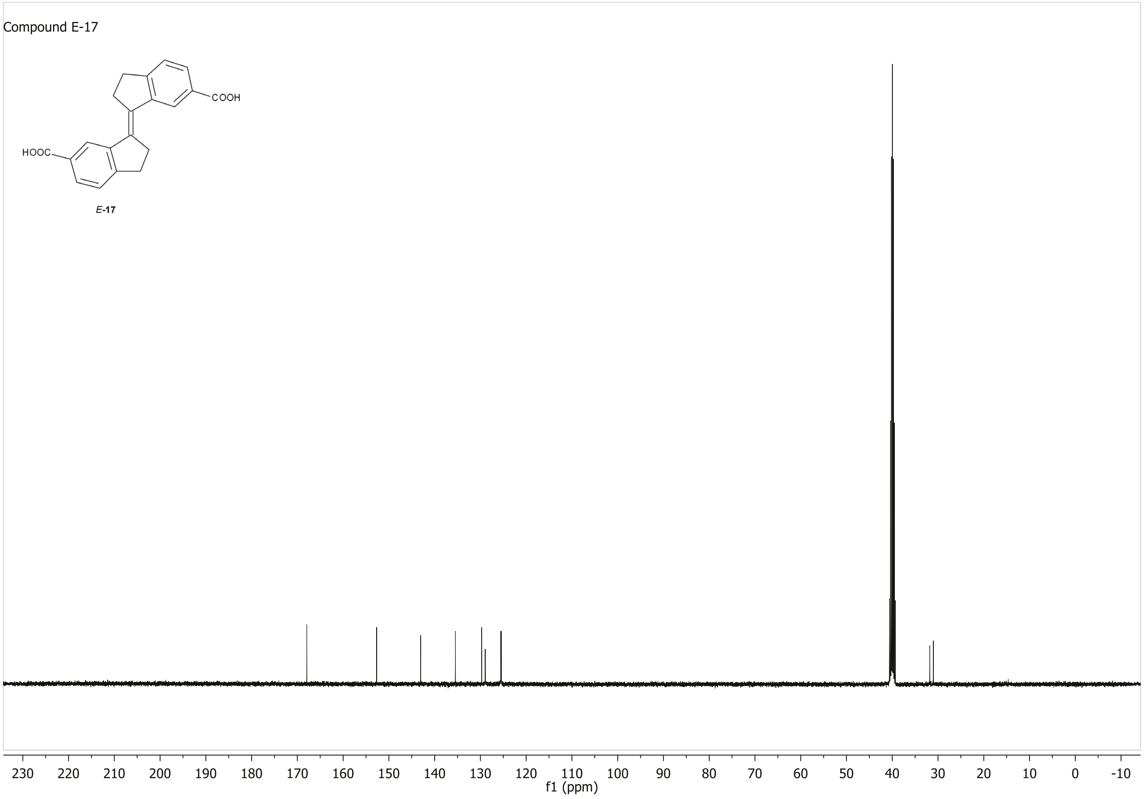

Figure S26. ^{13}C-NMR spectrum of *E*-**17** (100.6 MHz, DMSO-d$_6$ solution).

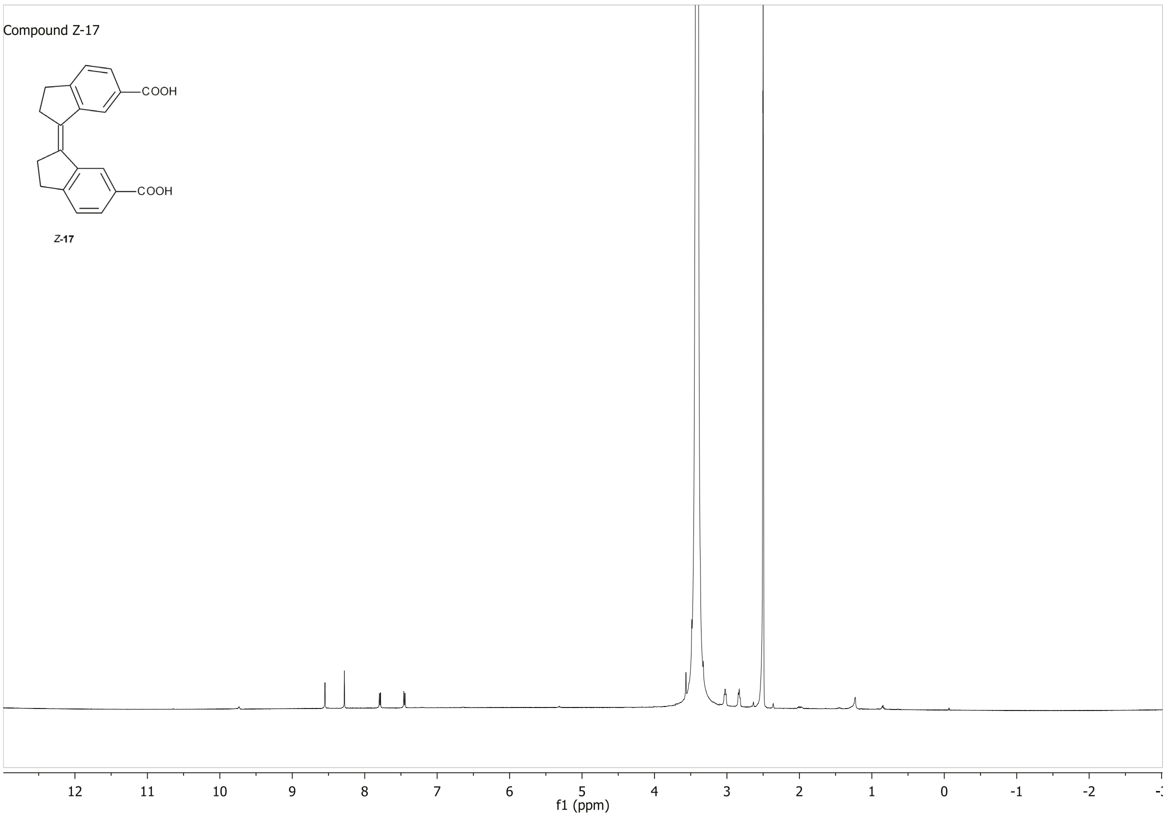

Figure S27. ^{1}H-NMR spectrum of *Z*-**17** (500 MHz, DMSO-d6 solution).

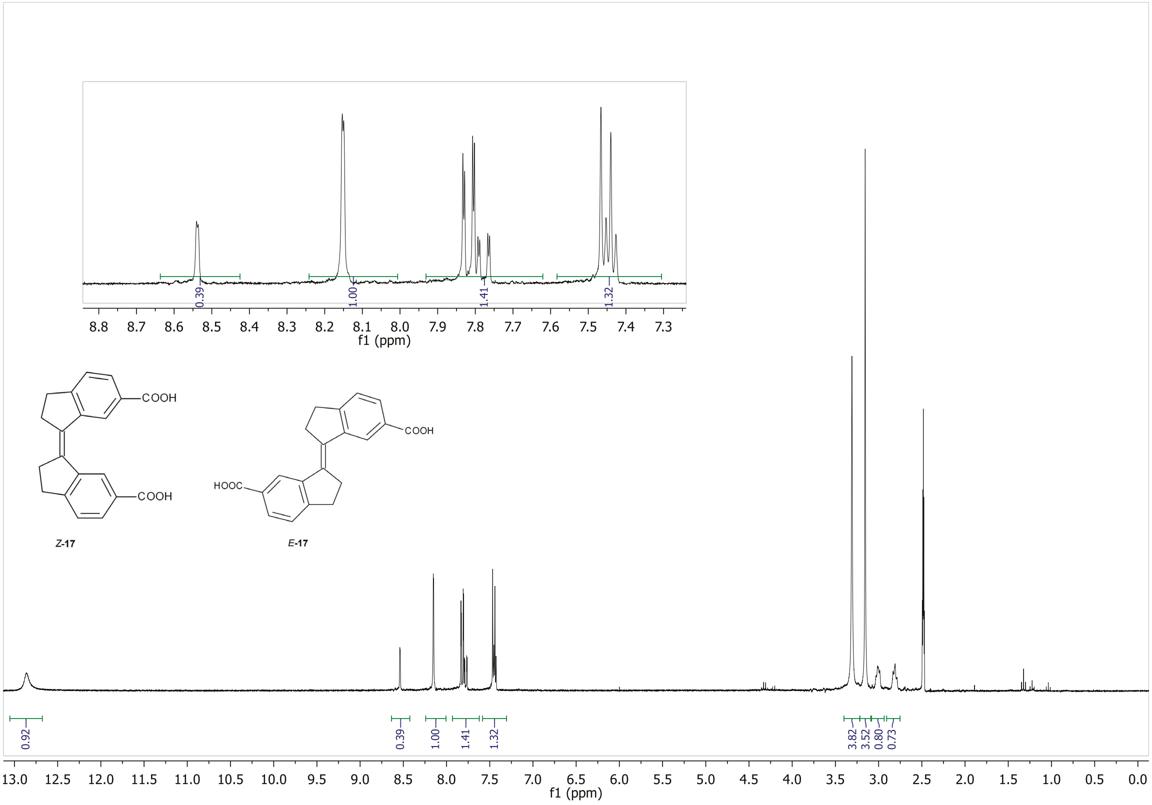

Figure S28. ^{1}H-NMR spectrum of *E*/*Z*-**17** photolysis product mixture (300 MHz, DMSO-d6 solution).

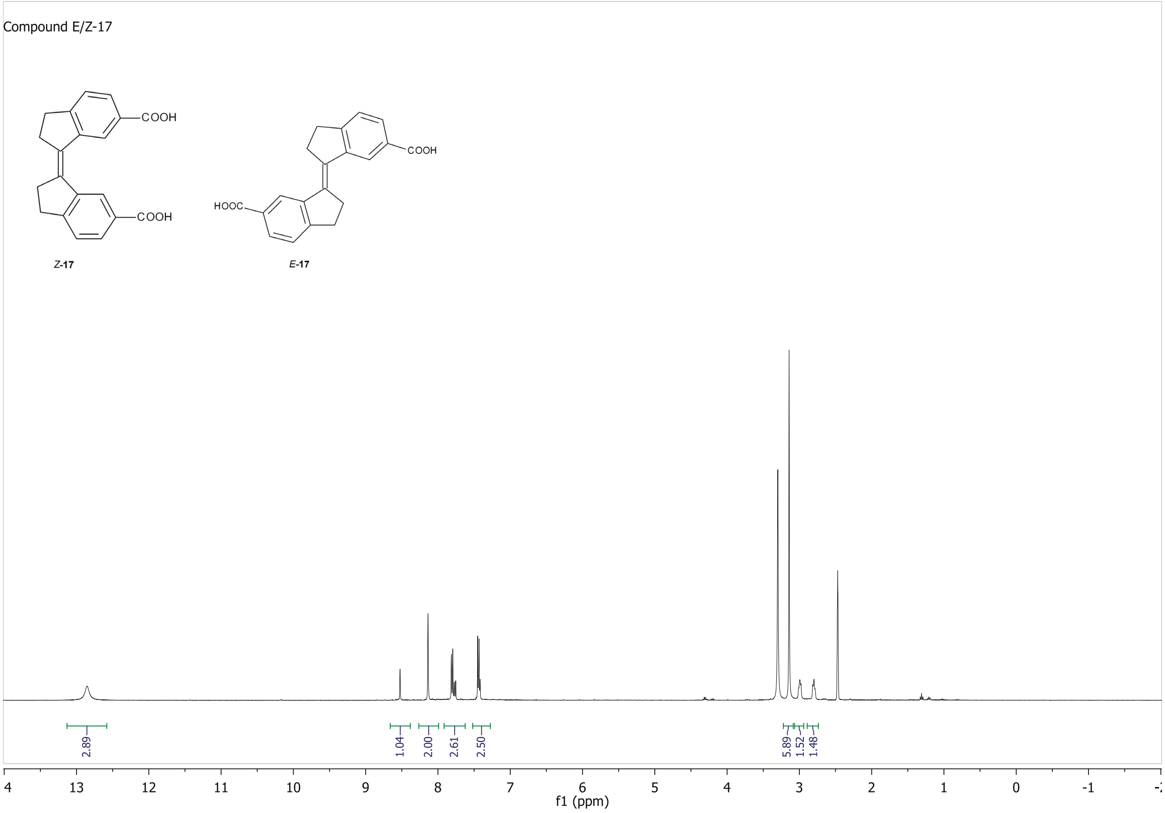

Figure S29. ^{1}H-NMR spectrum of *E*/*Z*-**17** photoisomerization product mixture (400 MHz, DMSO-d$_6$ solution).

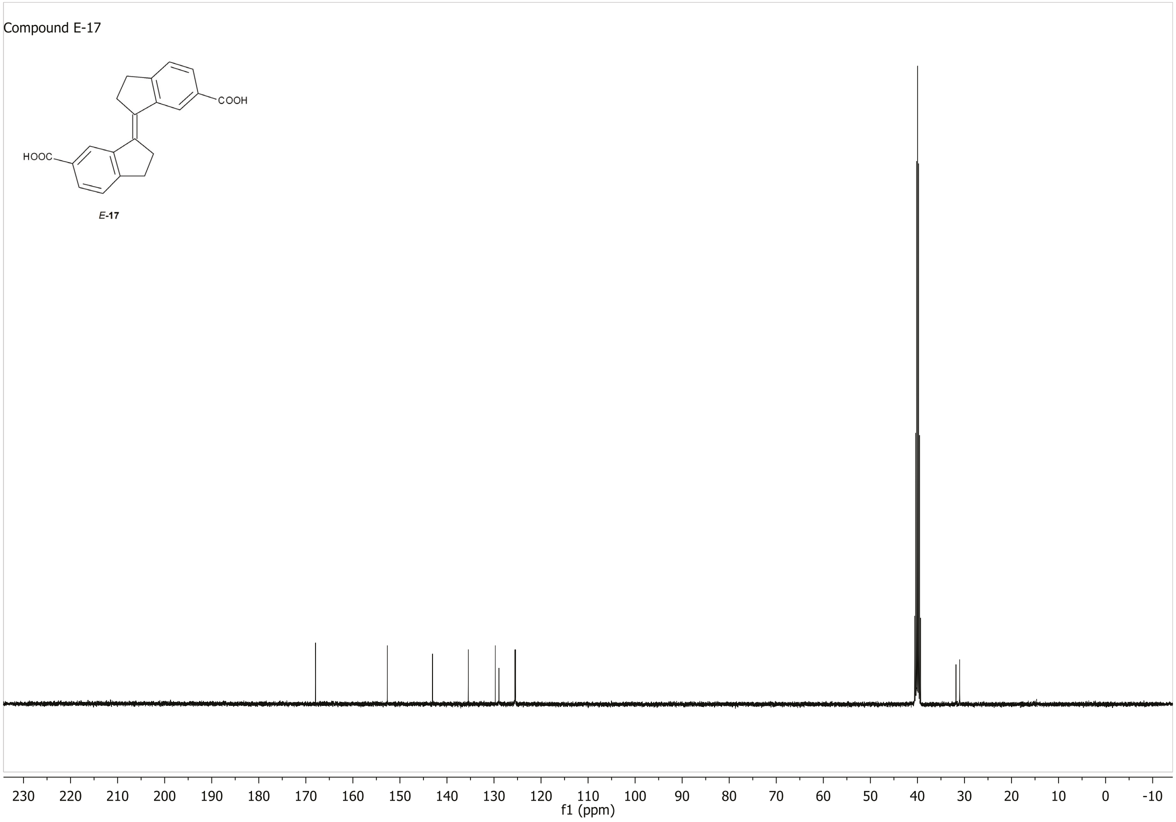

Figure S30. ^{13}C-NMR spectrum of *E*-**17** (100.6 MHz, DMSO-d$_6$ solution).

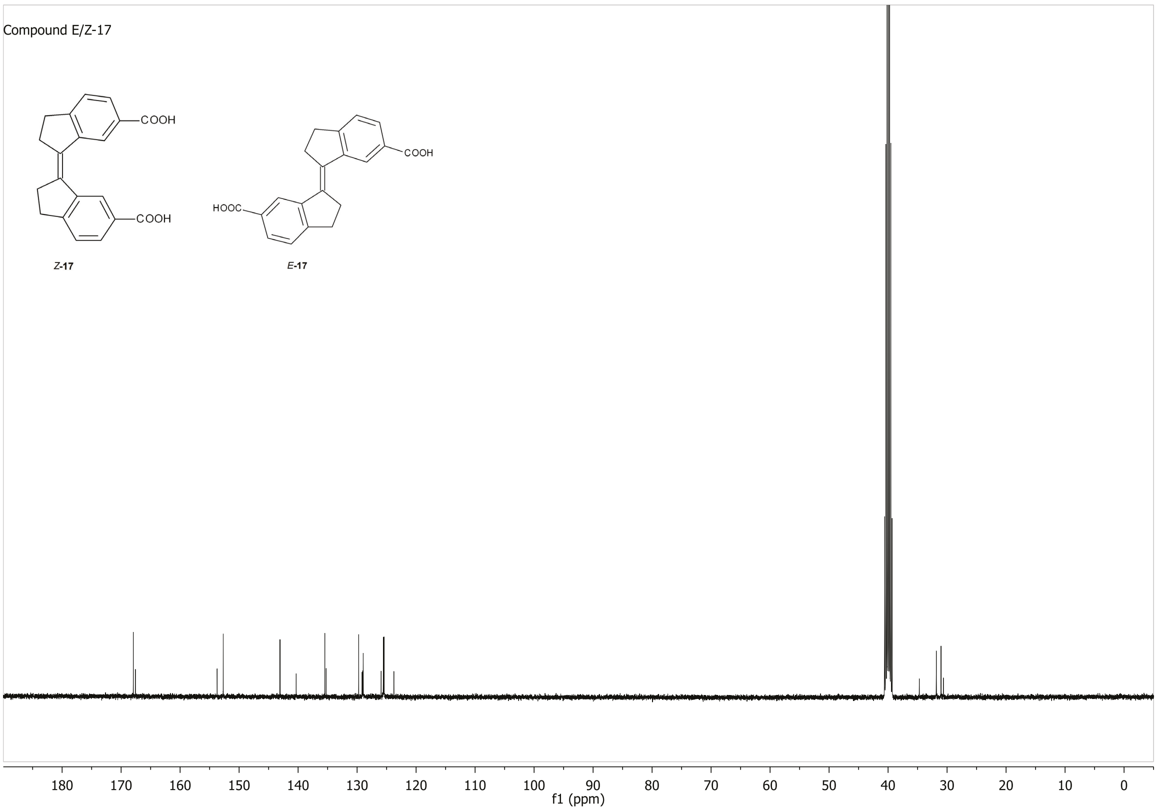

Figure S31. ^{13}C-NMR spectrum of *E*/*Z*-**17** photoisomerization product mixture (100.6 MHz, DMSO-d_6 solution).

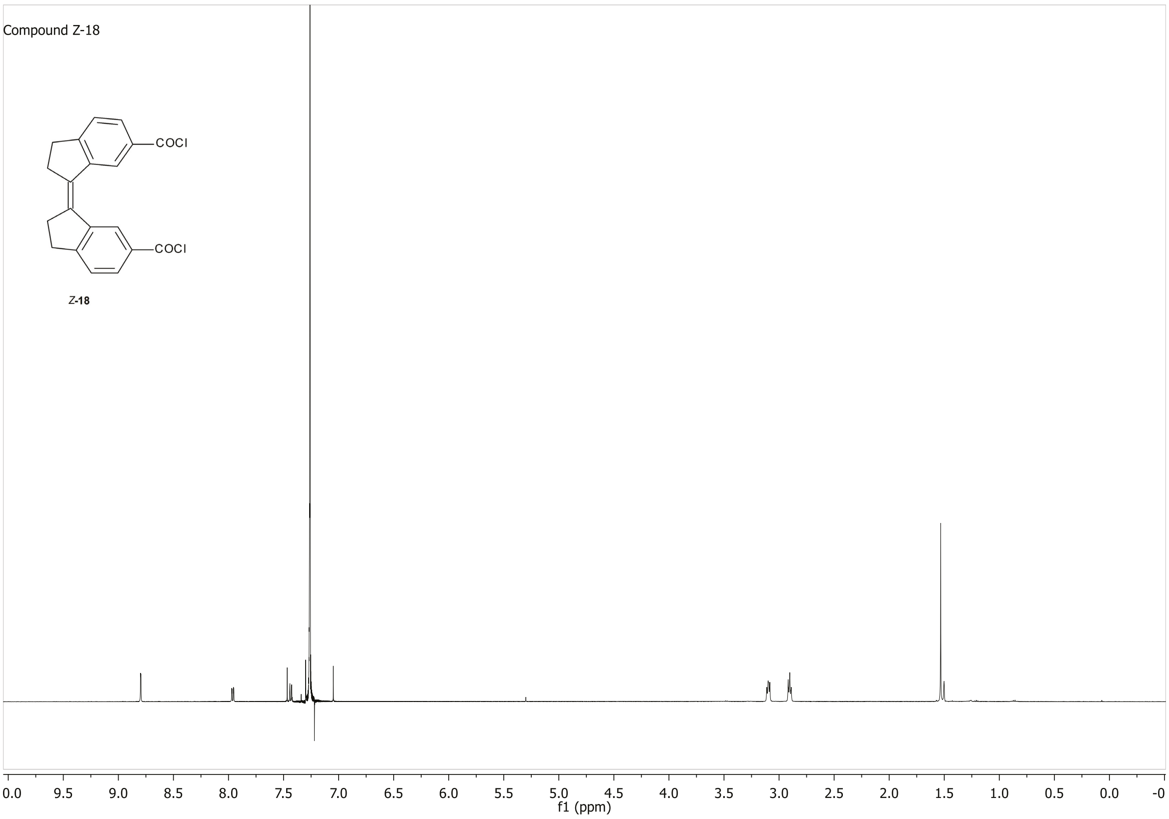

Figure S32. ^{1}H-NMR spectrum of *Z*-**18** (500 MHz, $CDCl_3$ solution).

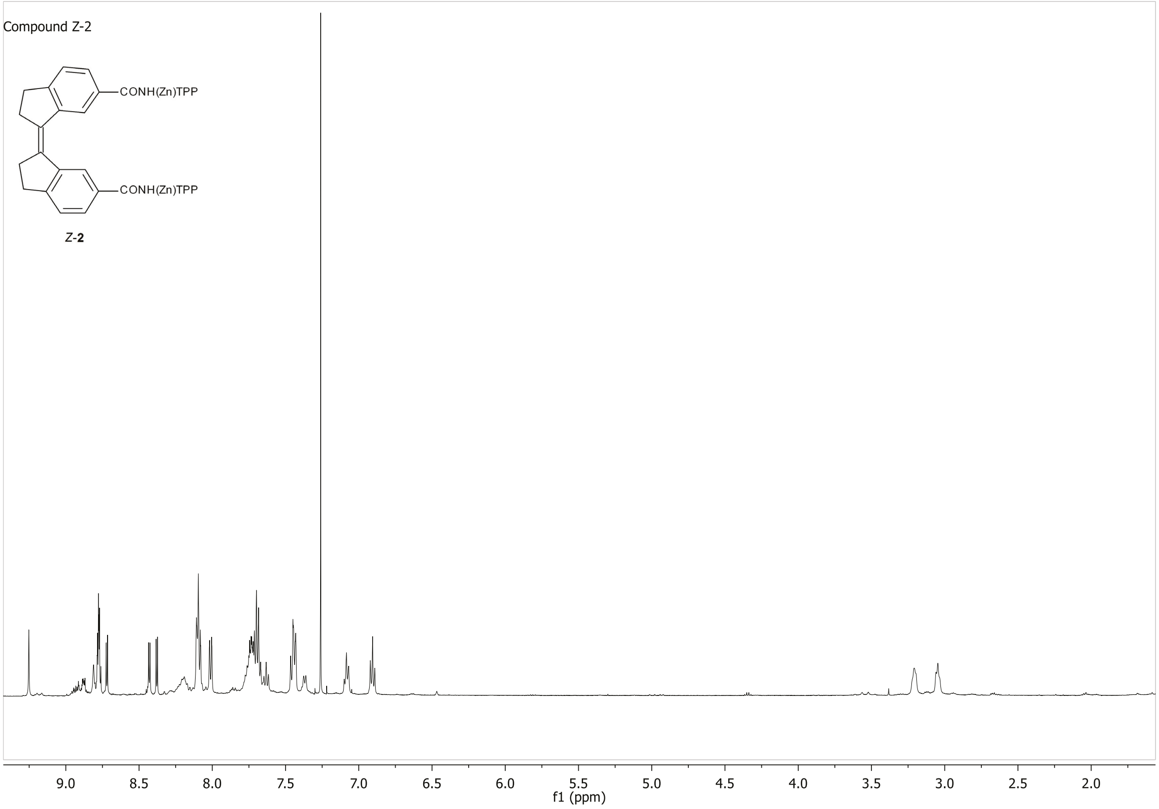

Figure S33. ^{1}H-NMR spectrum of *Z*-**2** (500 MHz, $CDCl_3$ solution).

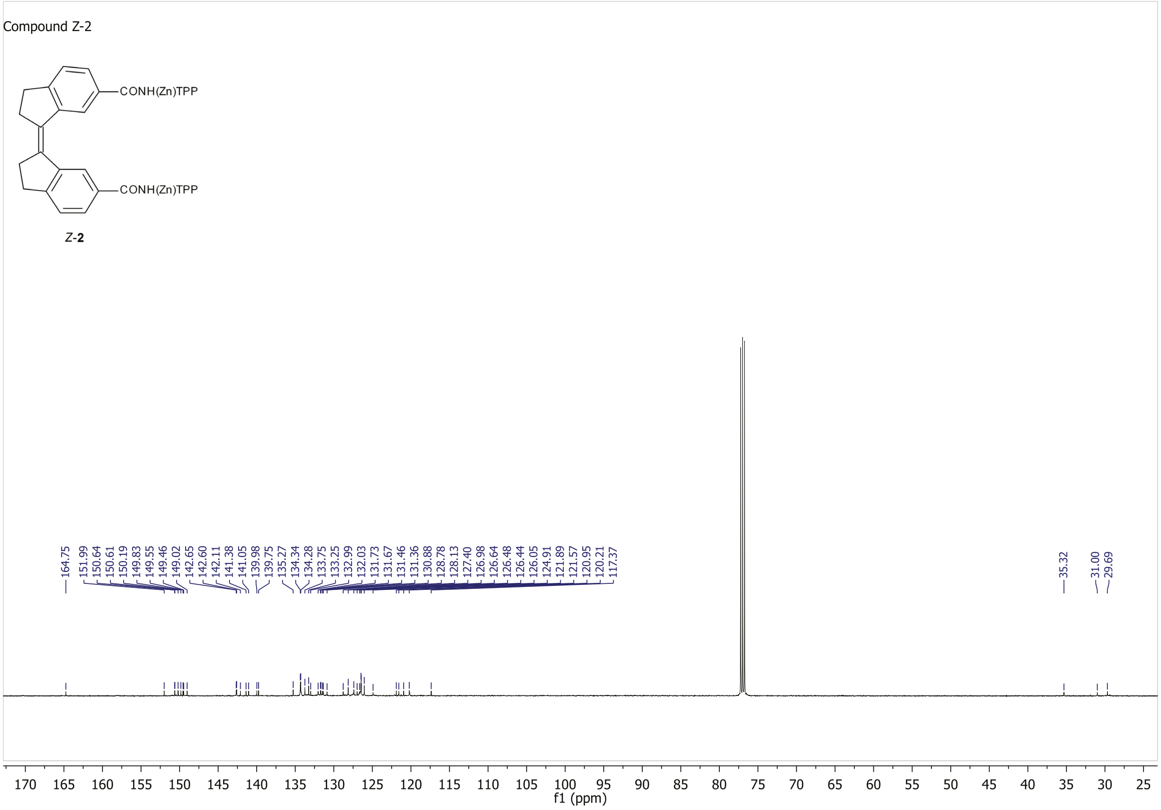

Figure S34. ^{13}C-NMR spectrum of *Z*-**2** (125.7 MHz, $CDCl_3$ solution).

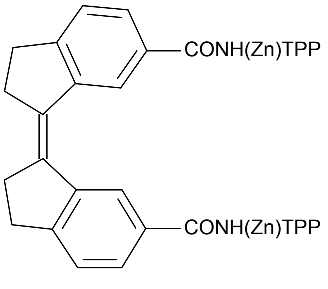

Z-**2**

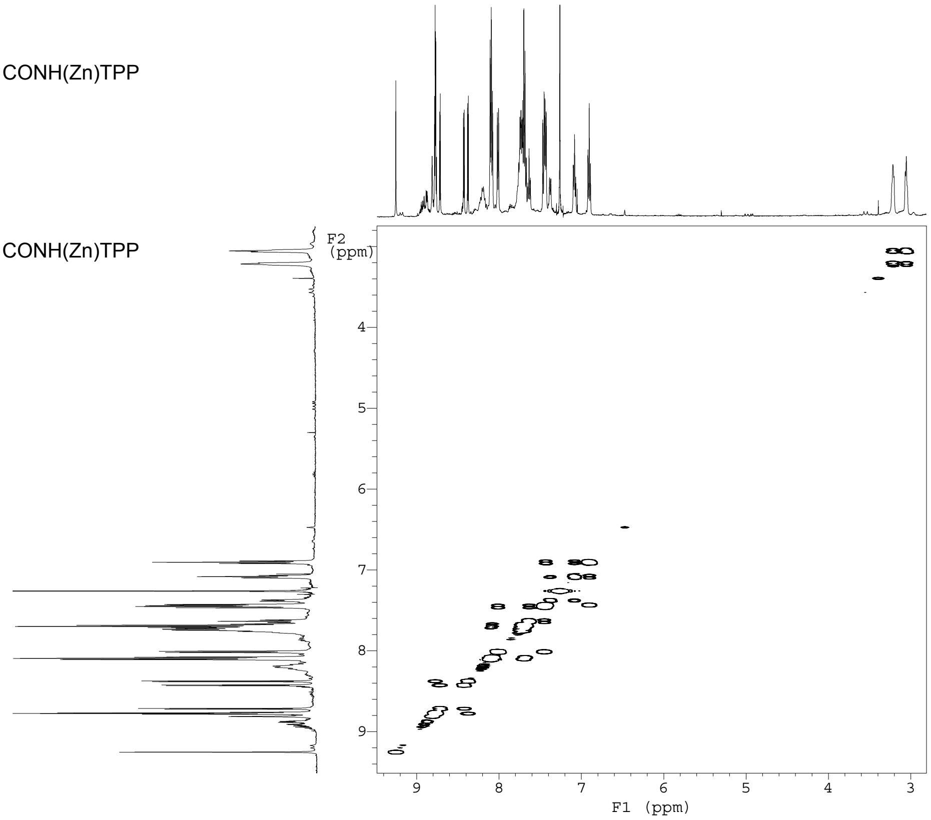

Figure S35. ^{1}H gCOSY spectrum of compound *Z*-**2** (500 MHz, $CDCl_3$).

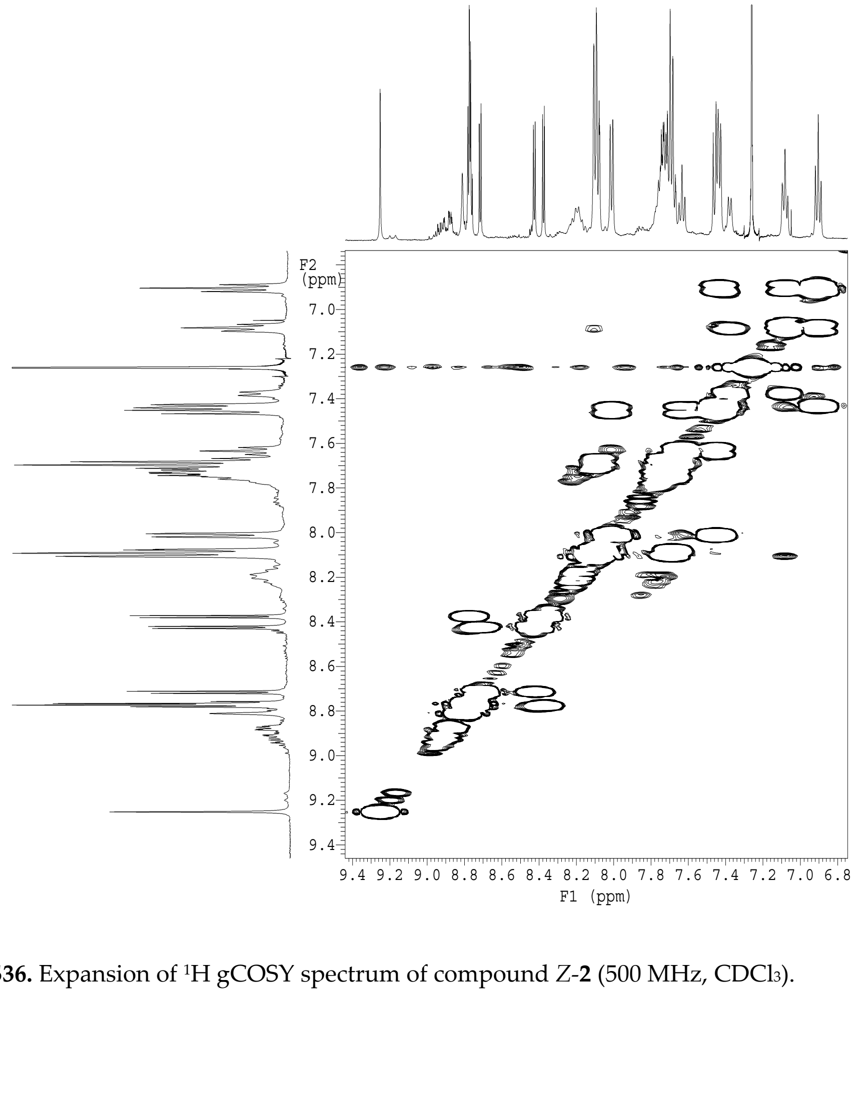

Figure S36. Expansion of ^{1}H gCOSY spectrum of compound *Z*-**2** (500 MHz, $CDCl_3$).

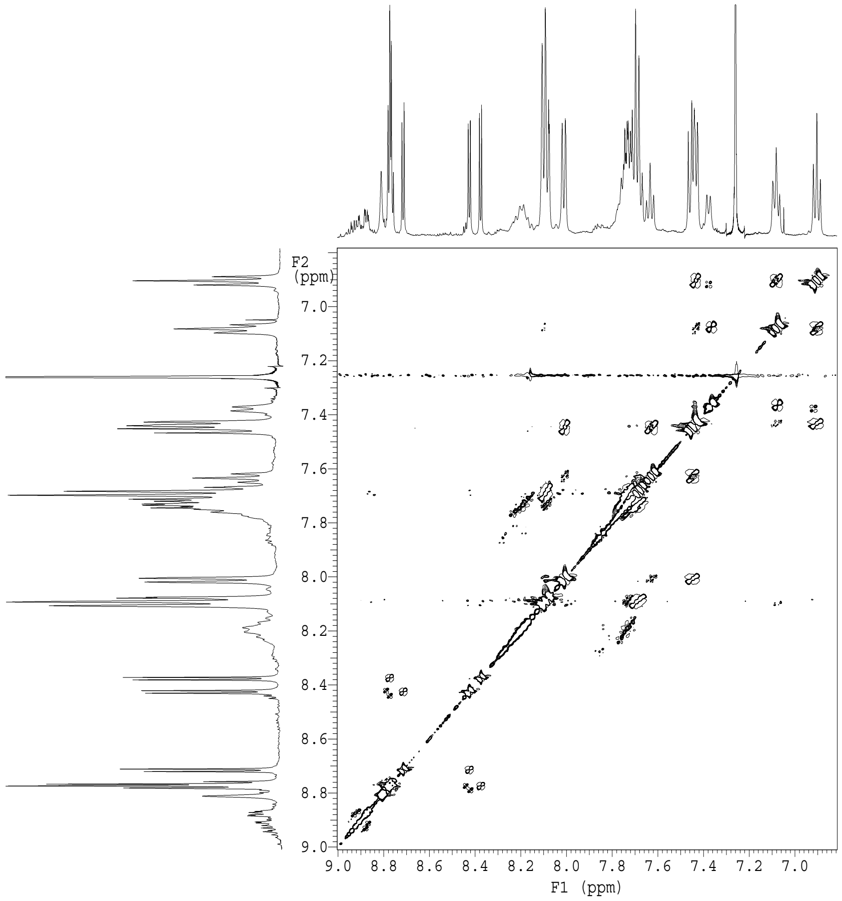

Figure S37. Expansion of P.E. COSY spectrum of compound *Z*-**2** (500 MHz, $CDCl_3$).

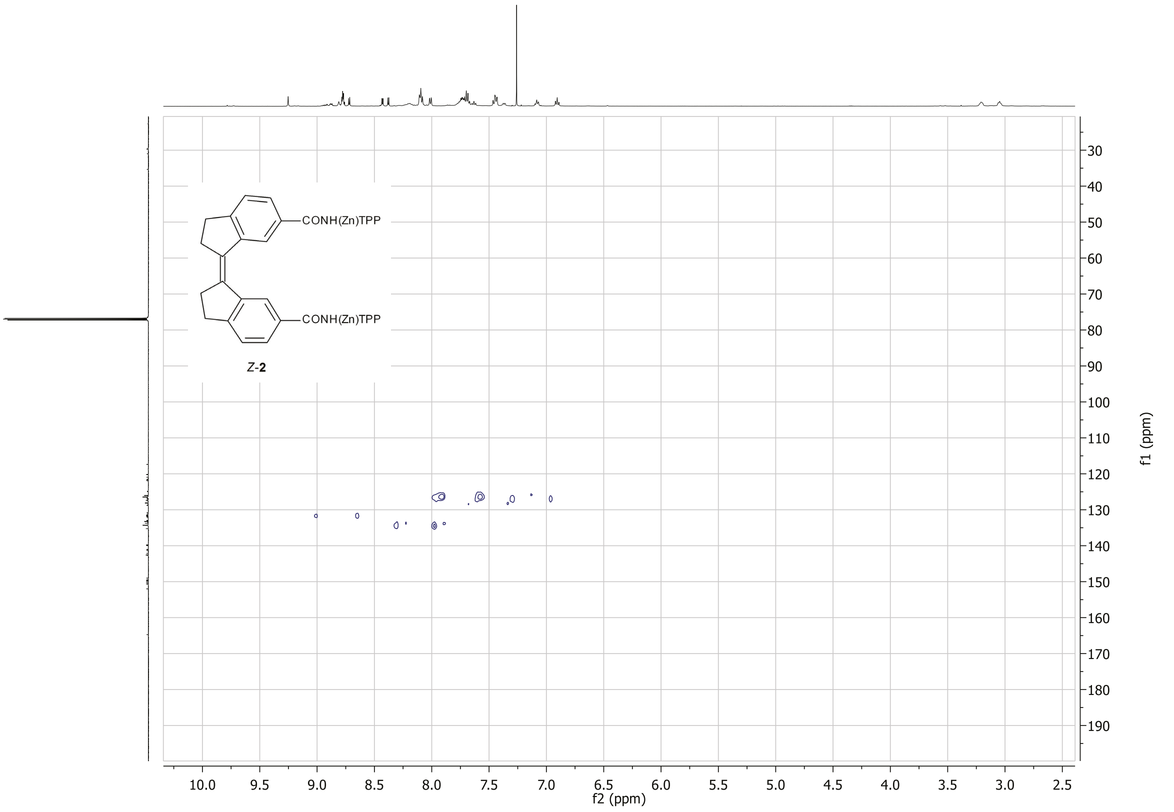

Figure S38. ^1H-^{13}C gHSQC spectrum of *Z*-**2** (500 MHz, $CDCl_3$ solution).

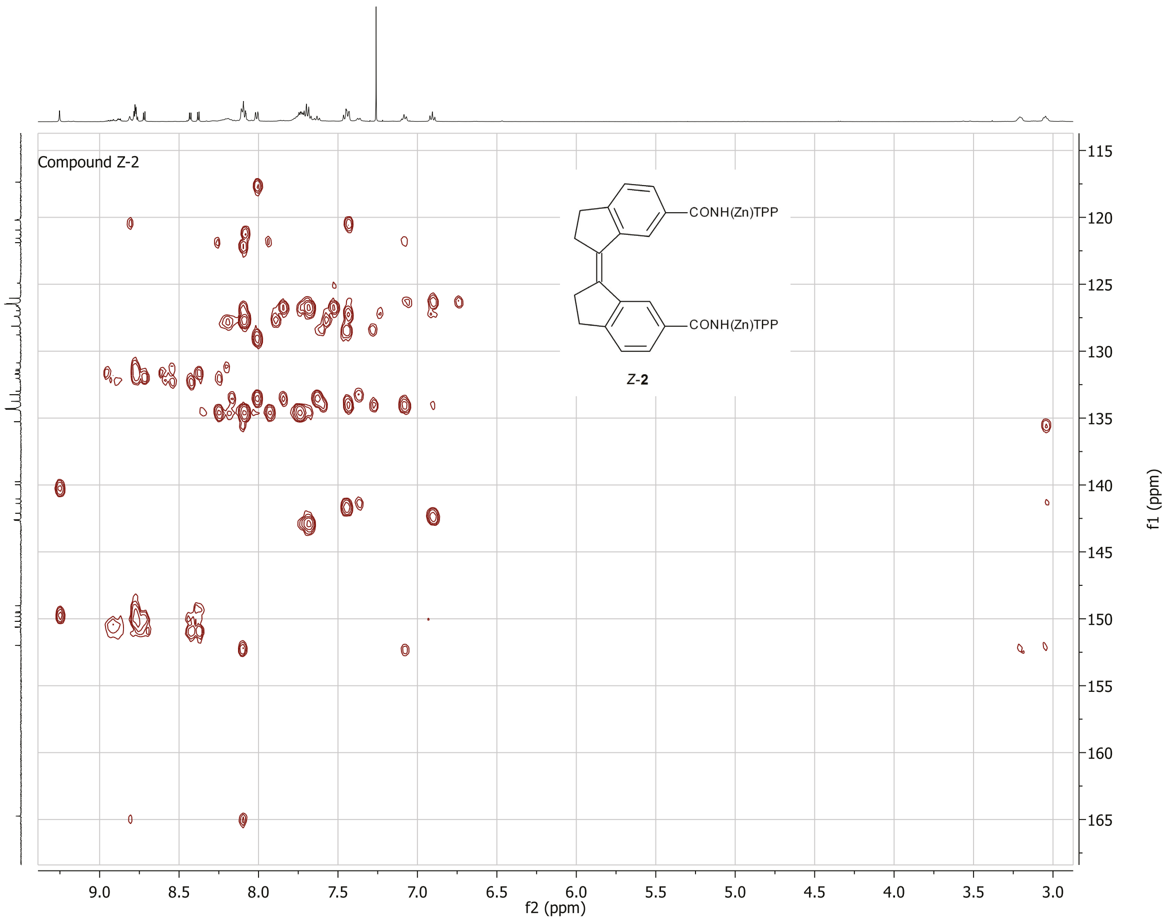

Figure S39. ^{1}H-^{13}C gHMBC spectrum of *Z*-**2** (500 MHz, $CDCl_3$ solution).

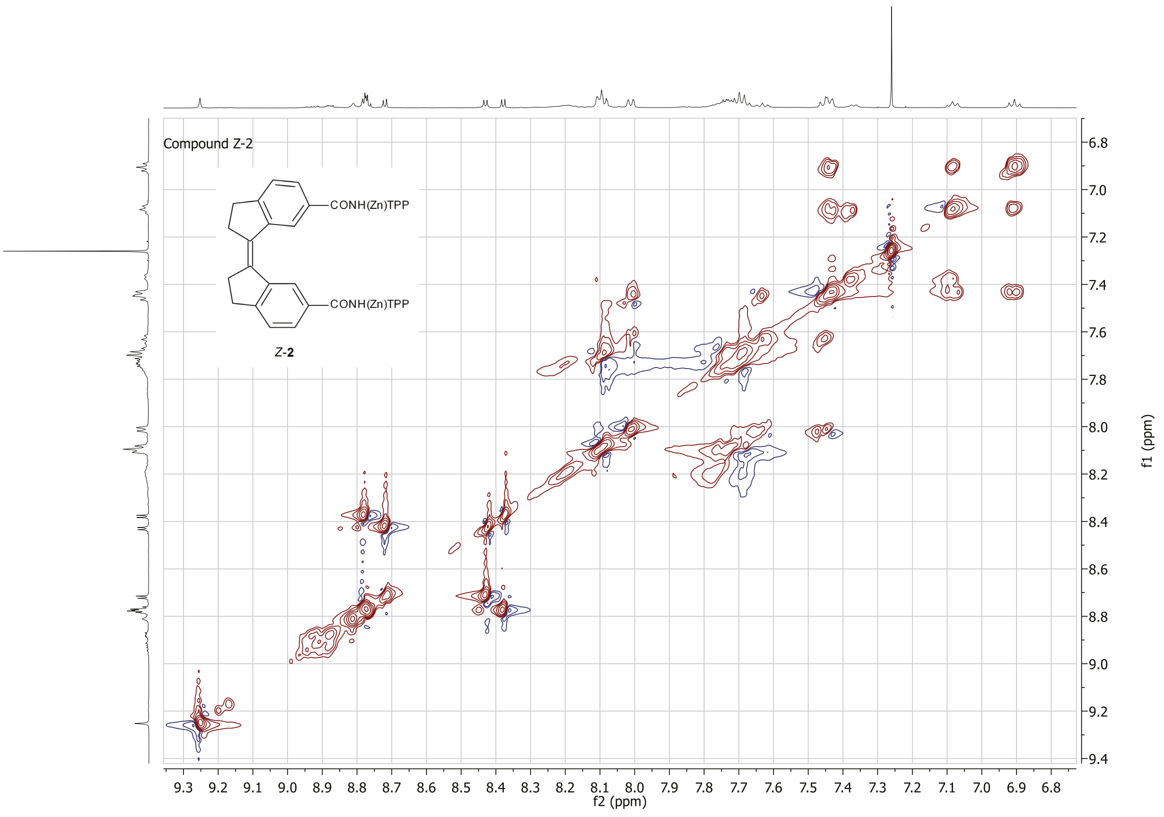

Figure S40. ^{1}H-^{13}C TOCSY spectrum of *Z*-**2** (500 MHz, $CDCl_3$ solution).

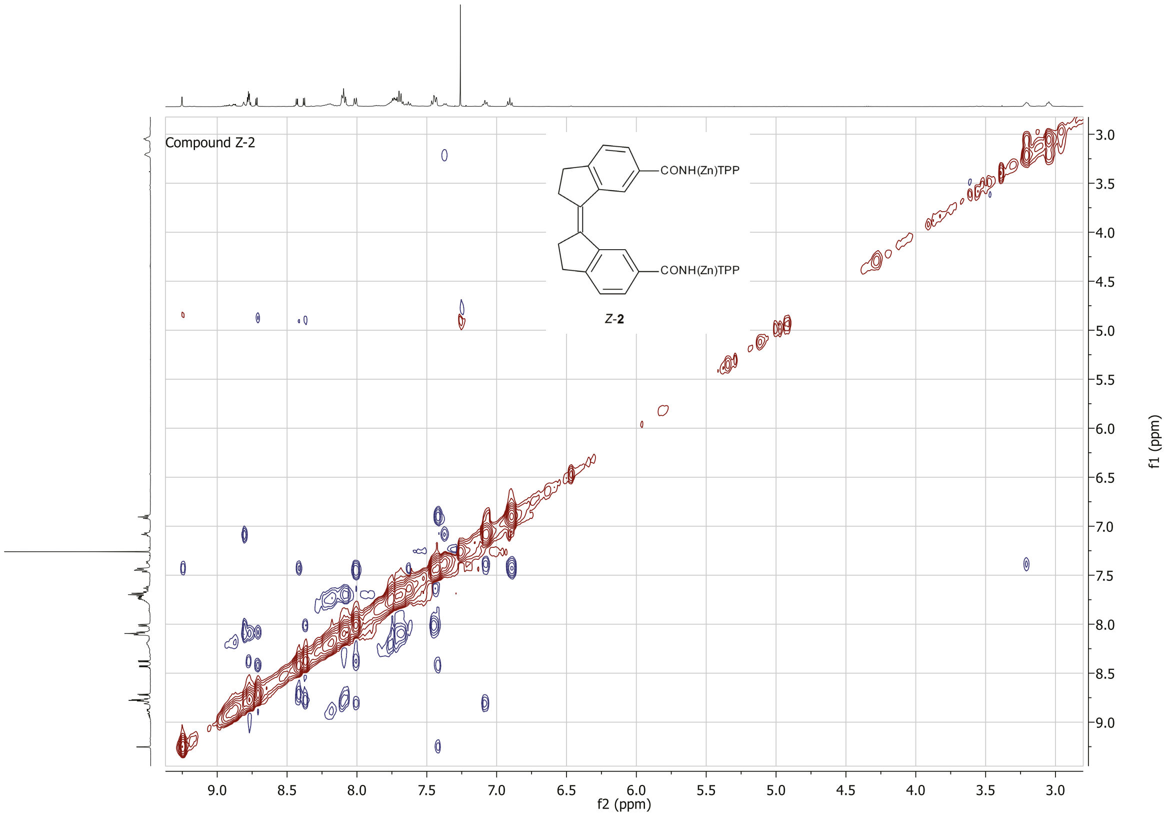

Figure S41. ^{1}H ROESY spectrum of *Z*-**2** (500 MHz, $CDCl_3$ solution).

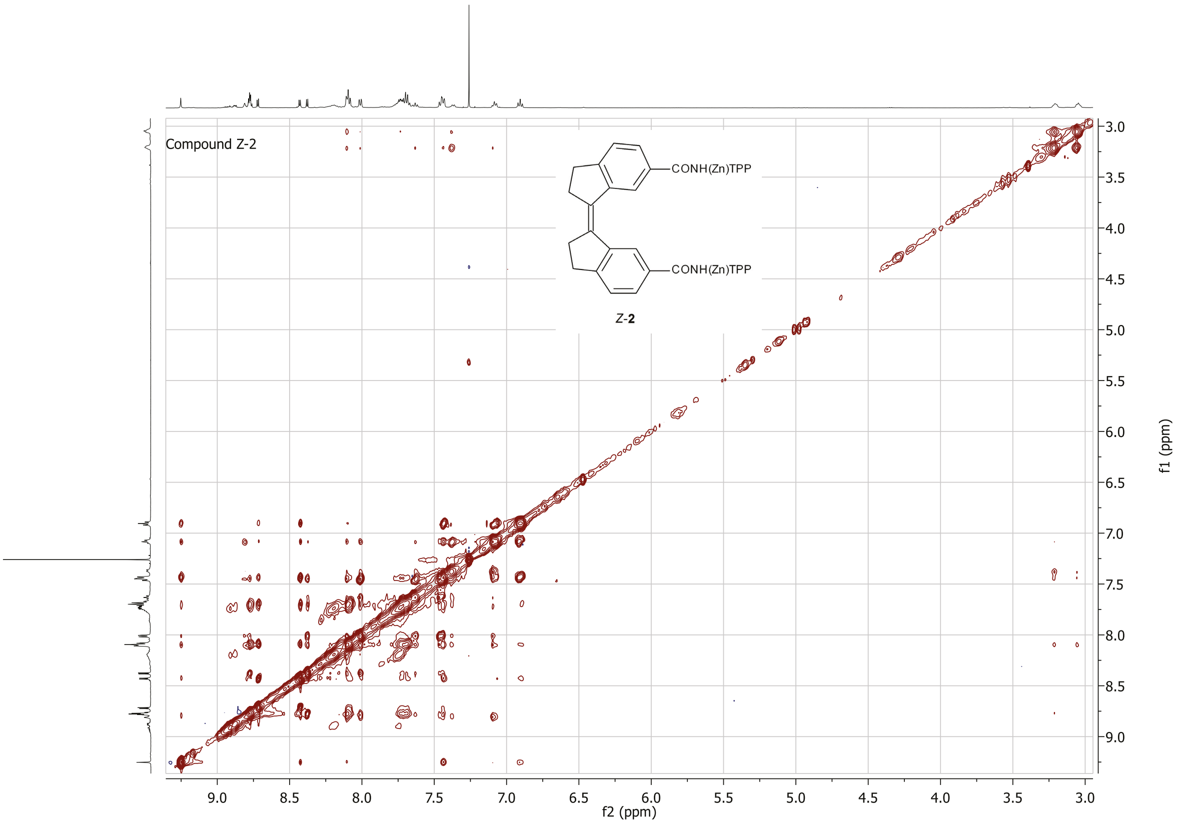

Figure S42. ^{1}H NOESY spectrum of *Z*-**2** (500 MHz, $CDCl_3$ solution).

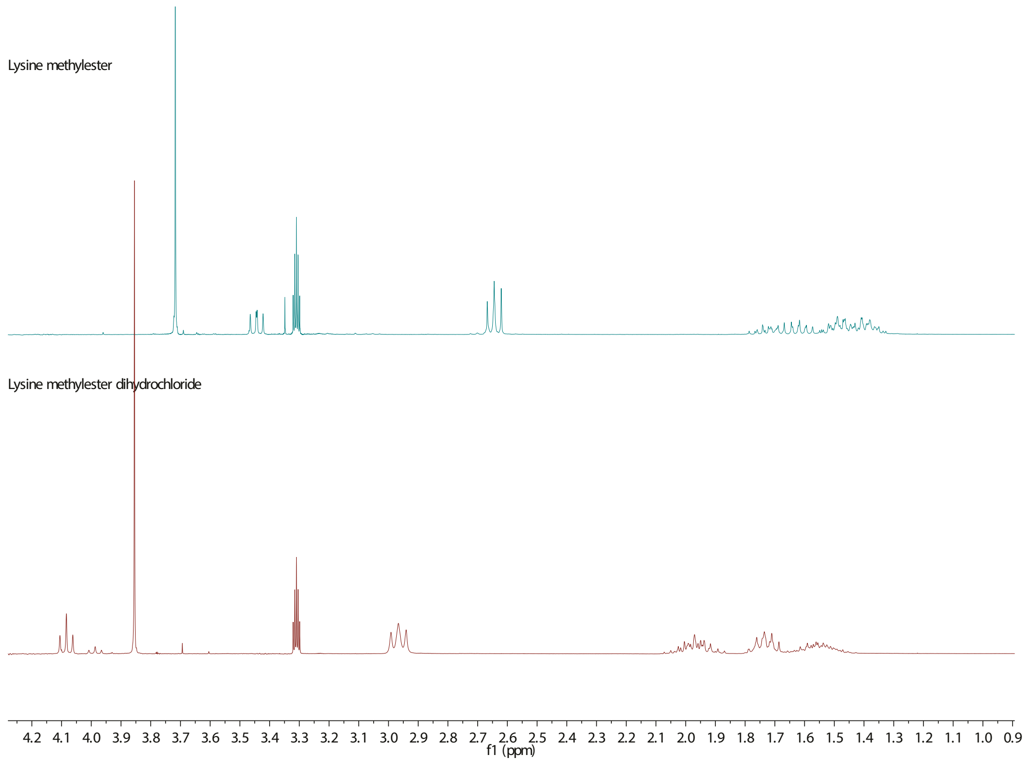

Figure S43. ^{1}H-NMR spectrum of *L*-lysine methyl ester **23** (top) and its hydrochloride (bottom) (300 MHz, CD_3OD solution).

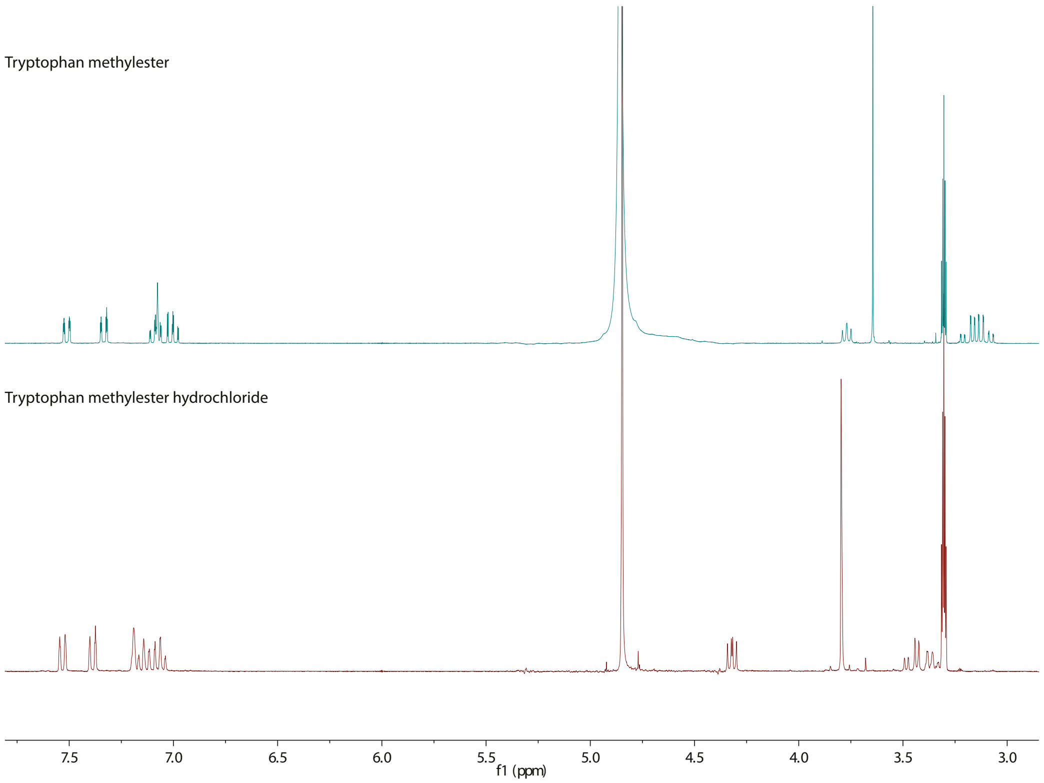

Figure S44. ^{1}H-NMR spectrum of *L*-tryptophan methyl ester **24** (top) and its hydrochloride (bottom) (300 MHz, CD_3OD solution).

4. Binding Studies with Aliphatic Diamine Guests

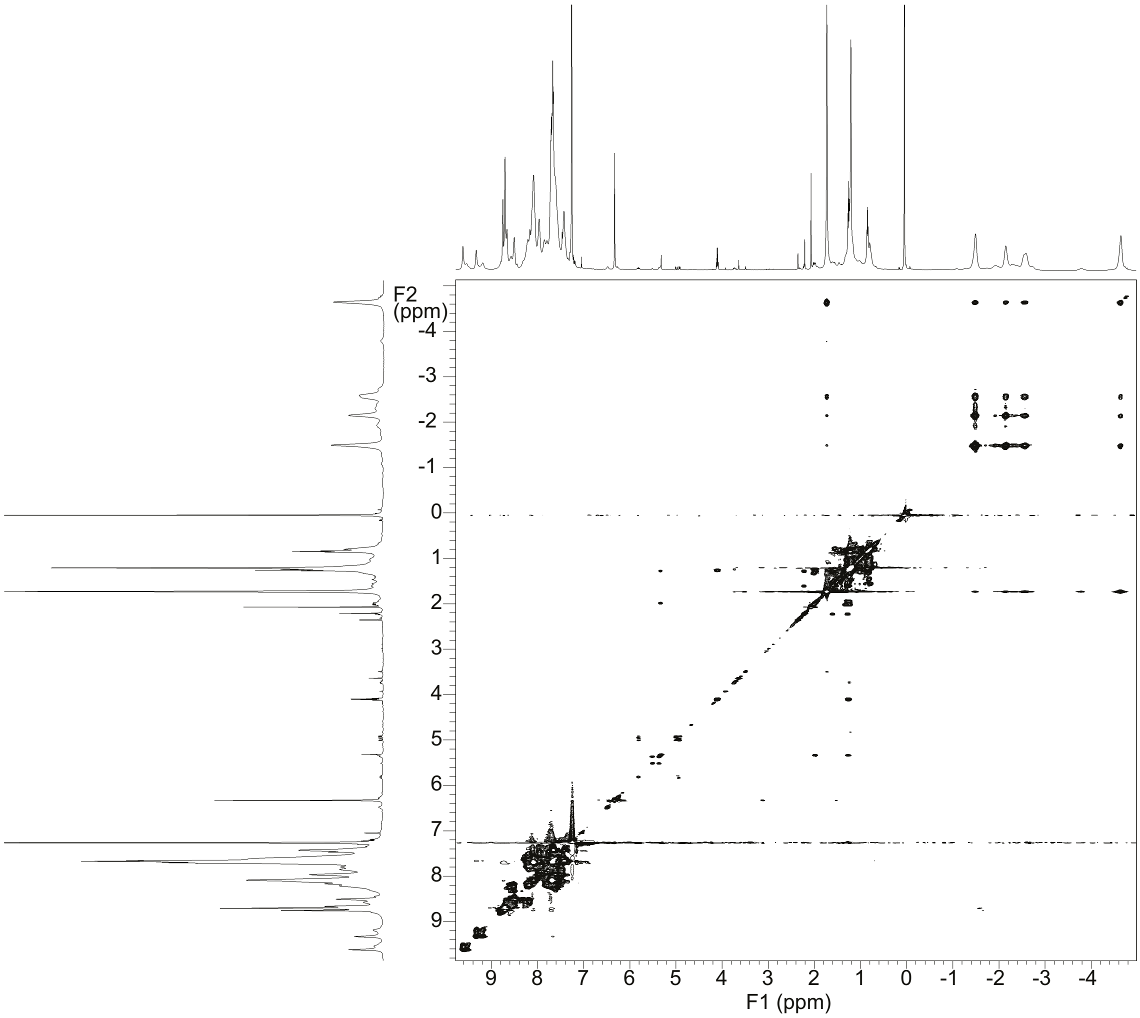

Figure S45. TOCSY spectrum of a ≈1:1 complex of enediyne tweezer *Z*-**1** and 1,6-diaminohexane **21** (500 MHz, $CDCl_3$. solution, −40 °C).

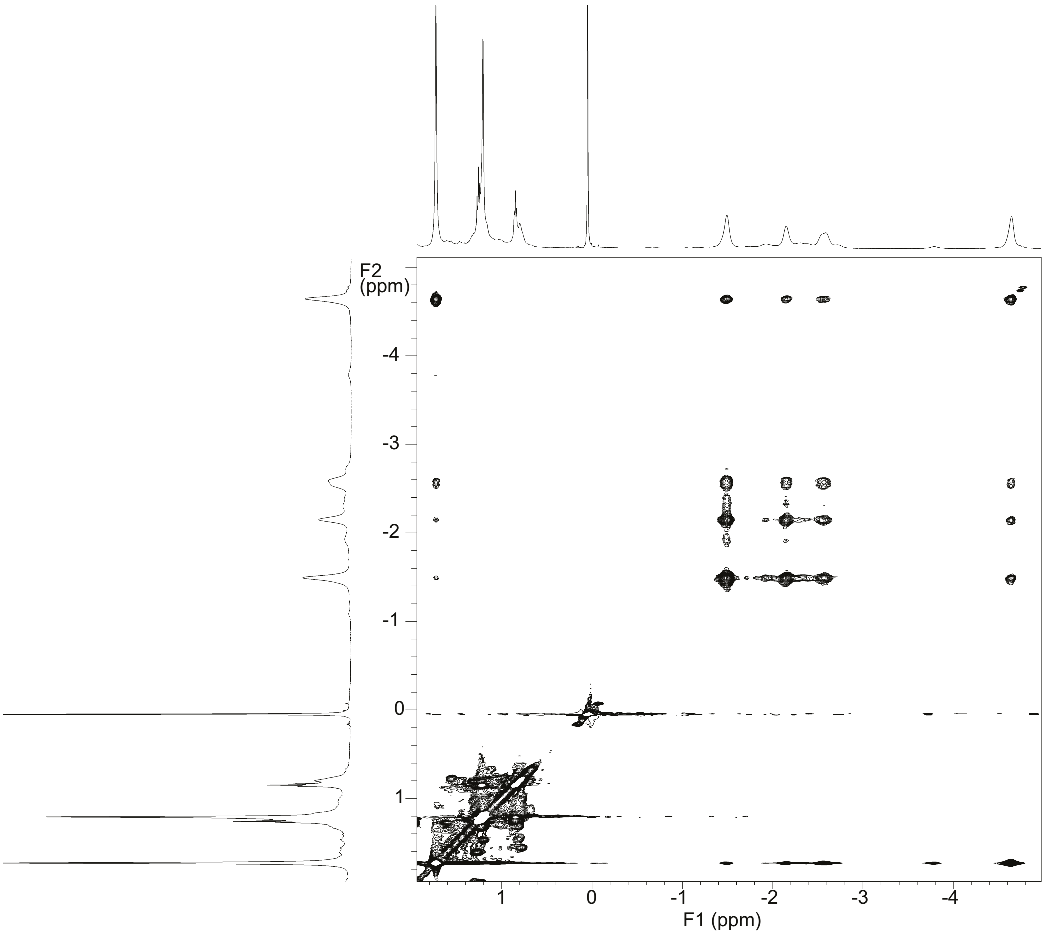

Figure S46. Expansion from TOCSY spectrum of a ≈1:1 complex of enediyne tweezer *Z*-**1** and 1,6-diaminohexane **21** (500 MHz, $CDCl_3$. solution, −40 °C).

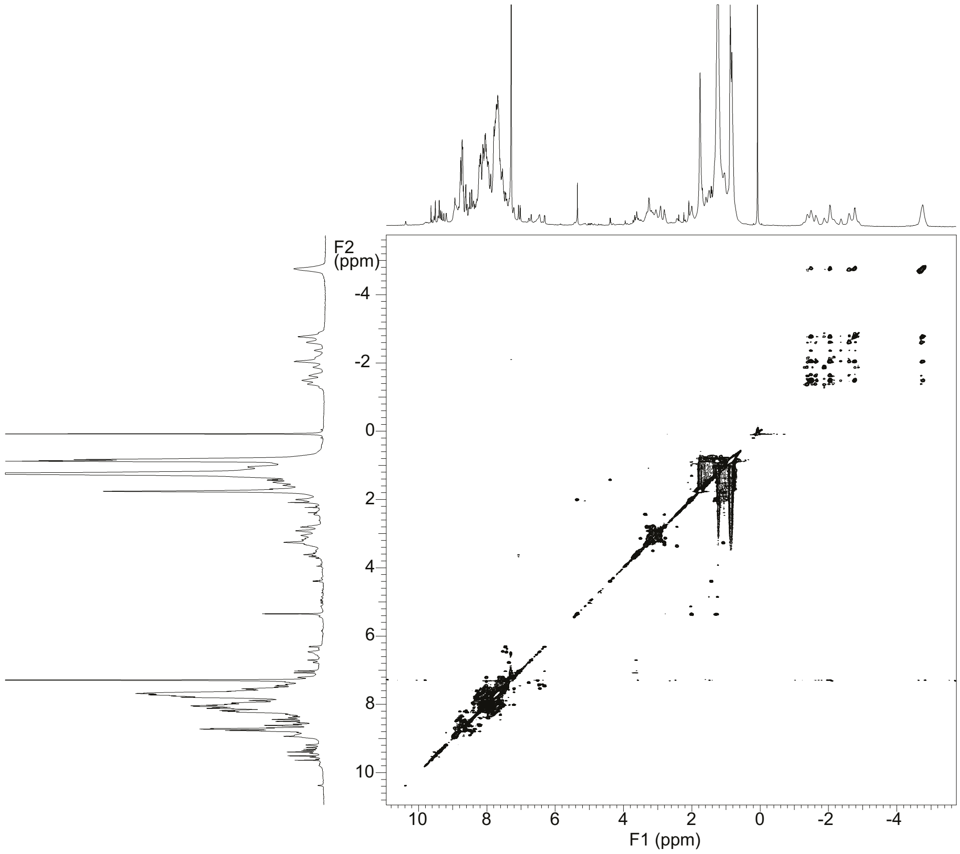

Figure S47. TOCSY spectrum of a ≈1:1 complex of tweezer *Z*-**2** and 1,6-diaminohexane **21** (500 MHz, $CDCl_3$. solution, −40 °C).

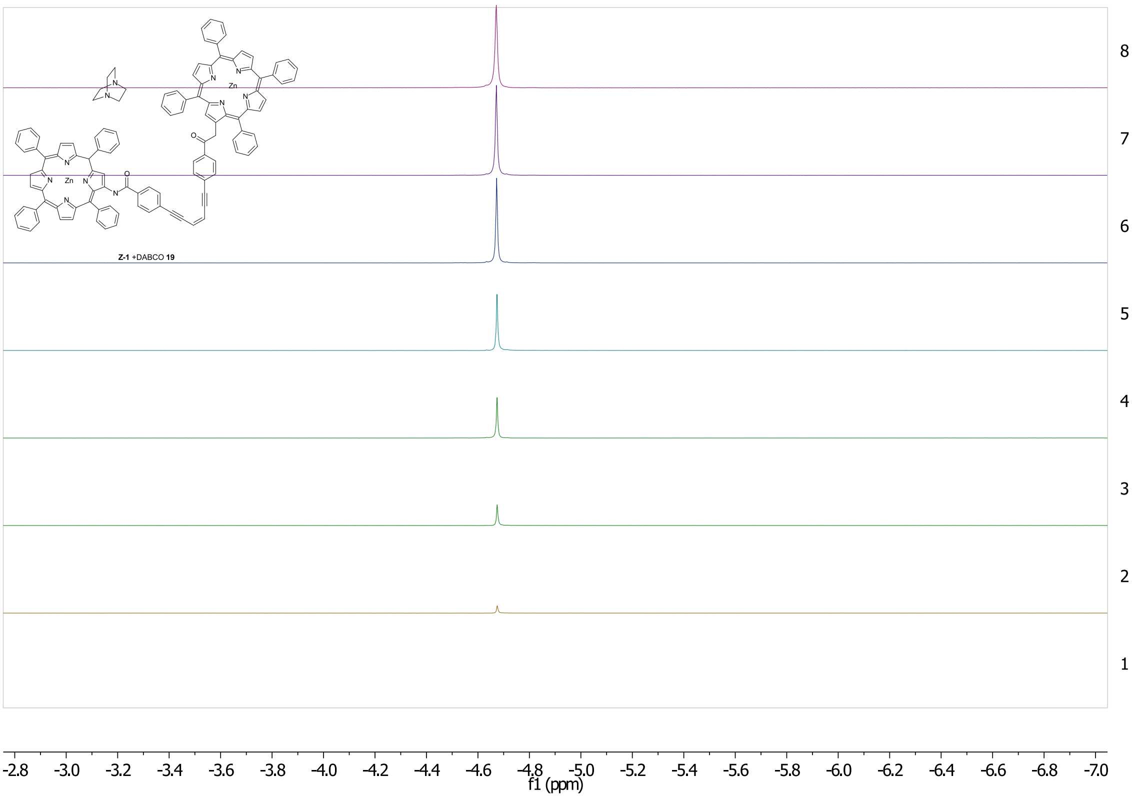

Figure S48. Expansion from ^{1}H-NMR spectra of of *Z*-**1** and increasing amount of DABCO **19** up to a ≈1:1 ratio, showing only one DABCO signal, thus indicating a symmetric DABCO environment.

5. UV-vis Spectra

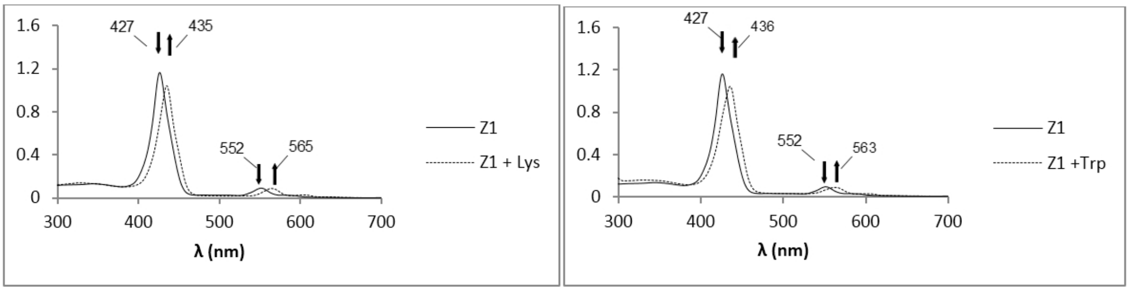

Figure S49. UV-vis spectra of bisporphyrin tweezer **Z-1** before and after complexation with L-lysine-methyl ester (left) and L-tryptophan methyl ester (right).

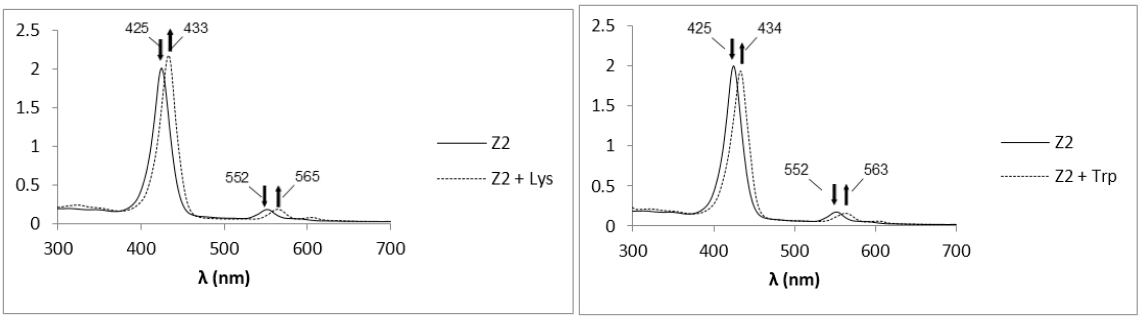

Figure S50. UV-vis spectra of bisporphyrin tweezer **Z-2** before and after complexation with L-lysine methyl ester **23** (left) and L-tryptophan methyl ester **24** (right).

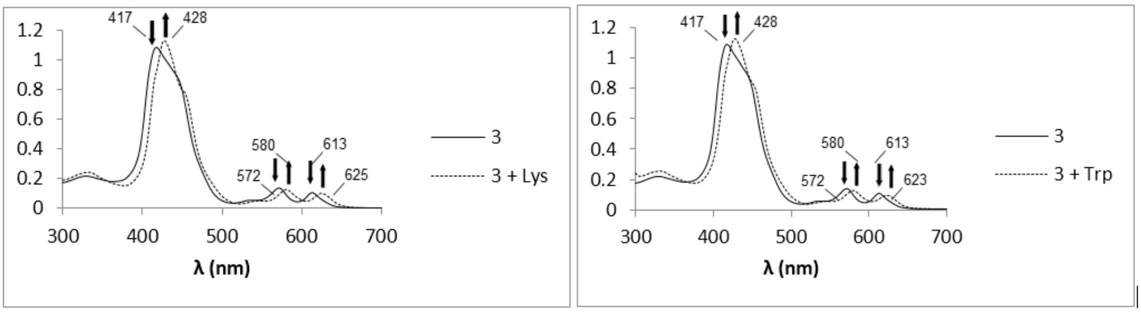

Figure S51. UV-vis spectra of bisporphyrin tweezer **3** before and after complexation with L-lysine methyl ester **23** (left) and L-tryptophan methyl ester **24** (right).

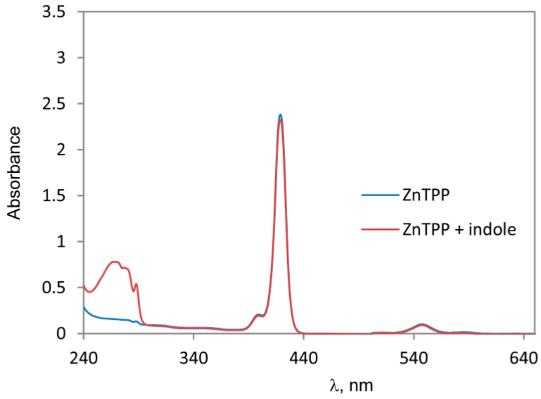

Figure S52. UV-vis spectra of Zn-TPP (-) and indole + Zn-TPP (-).

6. CD Spectra

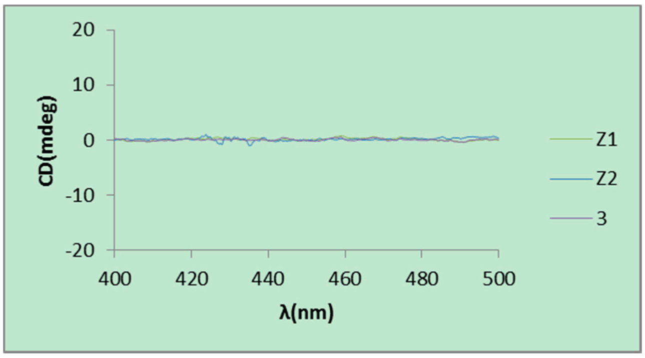

Figure S53. CD spectra of tweezers **1**, **2** and **3**.

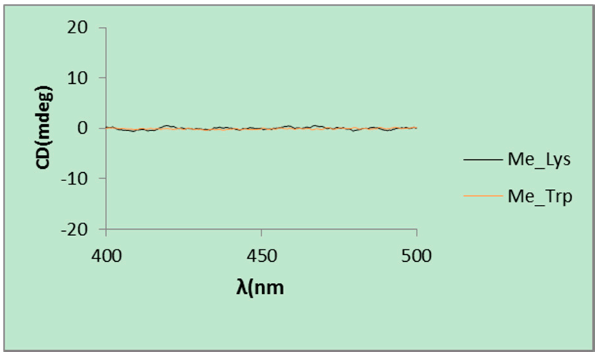

Figure S54. CD spectra of L-lysine and L-tryptophan methyl esters (**23** and **24**).

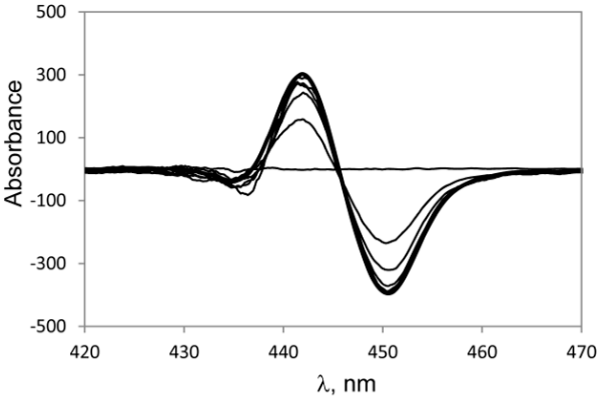

Figure S55. CD-spectra of titrations with **Z-2** (3.3×10^{-5}) in DCM with *L*-tryptophan methyl ester **24** as the host guest molar ratio increases from 0–1, 2, 4, 8,12, 20 equivalents, respectively.

7. Calculated Tweezer Structures

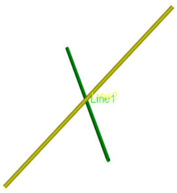

Tweezer **1** + LysMe **23**

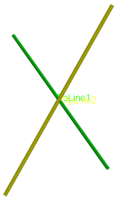

Tweezer **1** + TrpMe **24**

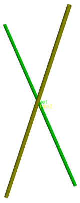

Tweezer **2** + LysMe **23**

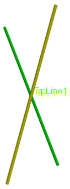

Tweezer **2** + TrpMe **24**

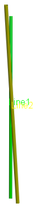

Tweezer **3** + LysMe **23**

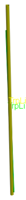

Tweezer **3** + TrpMe **24**

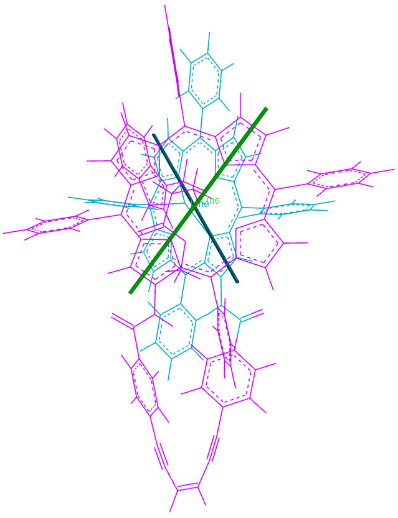

Tweezer **1** + TrpMe **24**

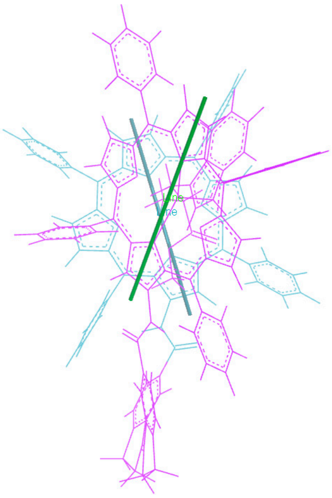

Tweezer **2** + TrpMe **24**

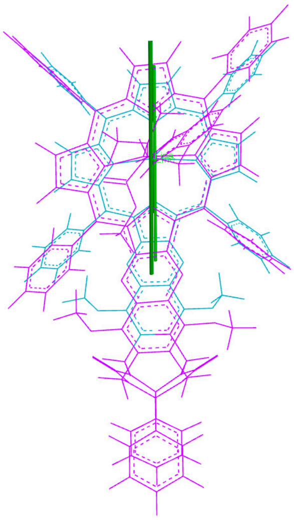

Tweezer **3** + TrpMe **24**

Scheme S1. Alignment of porphyrin axes in the complexes with chiral guests *L*-lysine methyl ester **23** and *L*-tryptophan methyl ester **24**. Line positions are indicated for **23** and **24** with tweezers **1**, **2** and **3**, respectively. Ditopic binding was assumed for **24**.

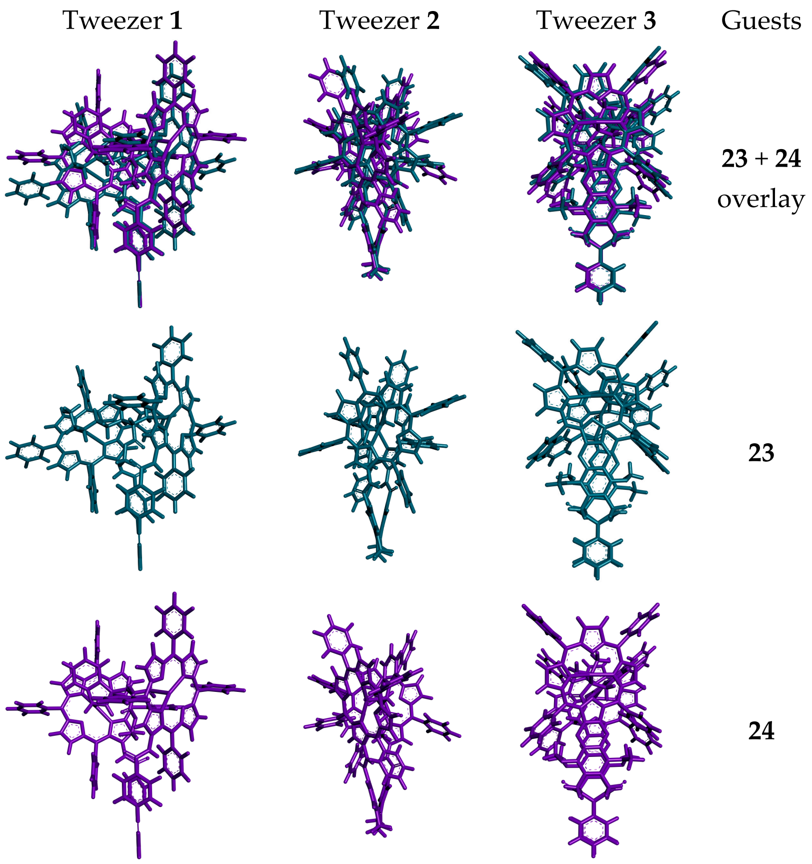

Scheme S2. Side views of complexes of tweezers **1**–**3** with L-lysine methyl ester **23** (green color) and L-tryptophan methyl ester **24** (magenta color). Shown are the structures corresponding to the global minimum obtained in conformational search with the OPLS 2005 force field. Ditopic binding was assumed for **24**.

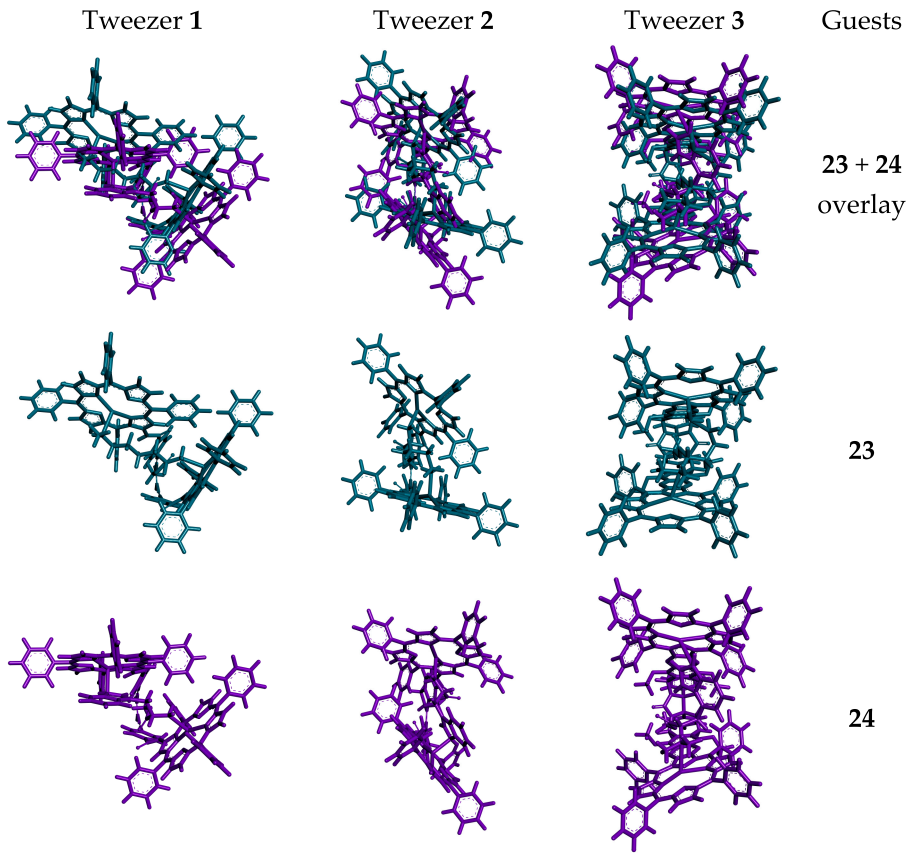

Scheme S3. Top views of complexes of tweezers **1**–**3** with L-lysine methyl ester **23** (green color) and L-tryptophan methyl ester **24** (magenta color). Shown are the structures corresponding to the global minimum obtained in conformational search with the OPLS 2005 force field. Ditopic binding was assumed for **24**.

References

1. Promarak, V.; Burn, P.L. A new synthetic approach to porphyrin-α-diones and a-2,3,12,13-tetraone: Building blocks for laterally conjugated porphyrin arrays. *J. Chem. Soc. Perkin Trans. I* **2001**, 14-20.
2. Giraudeau, A.; Callot, H. J.; Jordan, J.; Ezhar, I.; Gross, M. Substituent effects in the electroreduction of porphyrins and metalloporphyrins. *J. Am. Chem. Soc.* **1979**, *101*, 3857–3862.
3. Lee, J.C.; Song, I.-G.; Park, J.Y. Microwave promoted facile synthesis of methyl and ethyl carboxylates. *Synth. Commun.* **2002**, *32*, 2209–2213.
4. Erdélyi, M.; Gogoll, A. Rapid homogeneous-phase sonogashira coupling reactions using controlled microwave heating. *J. Org. Chem.* **2001**, *66*, 4165–4169.
5. Li, Q.; Rukavishnikov, A.; Petukhov, P.A.; Zaikova, T.O.; Jin, C.; Keana, J.F.W. Nanoscale tripodal 1,3,5,7-tetrasubstituted adamantanes for afm applications. *J. Org. Chem.* **2003**, *68*, 4862–4869.
6. Takeuchi, R.; Yasue, H. Rhodium complex-catalyzed desilylative cyclocarbonylation of 1-aryl-2-(trimethylsilyl)acetylenes: a new route to 2,3-dihydro-1H-inden-1-ones. *J. Org. Chem.* **1993**, *58*, 5386–5392.
7. Sankar, A.U.R.; Kumar, B.S.; Reddy, M.V.N.; Haribabu, B.; Raju, C.N. Synthesis and antimicrobial activity of novel (3a,S)-1-(aminoacid ester)-3a,4-dihydro-3H-1λ5-[1,3,2] oxazaphospholo[3,4-a]indol-1-oxides. *ARKIVOC* **2007**, 300–308.
8. Li, J.; Sha, Y. A convenient synthesis of amino acid methyl esters. *Molecules* **2008**, *13*, 1111–1119.
9. Norrehed, S.; Polavarapu, P.; Yang, W.; Gogoll, A.; Grennberg, H. Conformational restriction of flexible molecules in solution by a semirigid bis-porphyrin molecular tweezer. *Tetrahedron* **2013**, *69*, 7131–7138
10. Norrehed, S.; Johansson, H.; Grennberg, H.; Gogoll, A. Improved stereochemical analysis of conformationally flexible diamines by binding to a bisporphyrin molecular clip. *Chem. Eur. J.* **2013**, *19*, 14631–14638.
